# Ortho-phosphite $\left({\mathrm{PO}}_{3}^{3-}\right)$ : Mechanochemical Synthesis of a Missing Oxoanion and Precursor to Value-Added Organophosphorus Compounds

**DOI:** 10.1021/acscentsci.5c01595

**Published:** 2025-11-26

**Authors:** Pawel Löwe, Rachid Taakili, Tiansi Xin, Hritwik Haldar, Antonia Herzog, Yang Shao-Horn, Christopher C. Cummins

**Affiliations:** † Department of Chemistry, 2167 Massachusetts Institute of Technology, Cambridge, Massachusetts 02139, United States; ‡ Research Laboratory of Electronics, Massachusetts Institute of Technology, Cambridge, Massachusetts 02139, United States

## Abstract

Despite the ubiquity of the well-known phosphorus polyanion phosphite $\left({\mathrm{HPO}}_{3}^{2-}\right)$ , it appears to be the case that no simple salt of the corresponding conjugate base $({\mathrm{PO}}_{3}^{3-}$ , “ortho-phosphite”) has ever been reported. We report the synthesis and characterization of this elusive species as a major component of a mixture obtained upon mechanochemical reduction of condensed phosphates, as evidenced by solid-state ^31^P NMR and Raman spectroscopy as well as subsequent reactivity studies. To corroborate the ^31^P NMR spectroscopic assignment, we independently generated Na_3_PO_3_ and K_3_PO_3_ by deprotonation of Na_2_HPO_3_ with NaCH_2_SiMe_3_ and of K_2_HPO_3_ with KCH_2_Ph, respectively, providing an orthogonal route to ${\mathrm{PO}}_{3}^{3-}$ salts whose spectroscopic signatures match those observed in the mixture obtained by mechanochemical reduction. We further found that ortho-phosphite can act as a precursor for various phosphorus chemicals, such as P­(OSiMe_3_)_3_ (46%), which is already well established as a precursor to a plethora of useful organophosphorus compounds. Therefore, our results not only establish the first formal pathway from P­(V) phosphate starting materials to P­(OSiMe_3_)_3_ without the intermediacy of white phosphorus, but also open the door to a broad range of downstream transformations based on this sustainable pathway. Additionally, BaHPO_3_·H_2_O (66%), OP­(OMe)_2_Me (DMMP), and OP­(OBn)_2_Bn (DBBP) have been generated directly from ortho-phosphite, all traditionally synthesized from white phosphorus.

## Graphical abstract

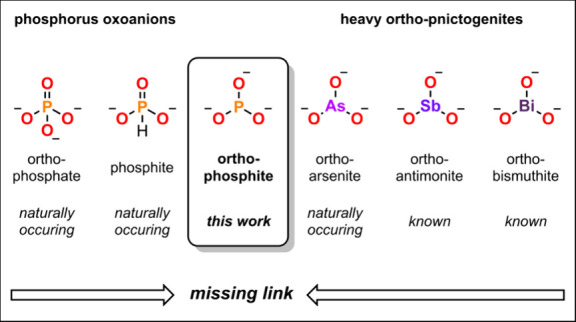

## Introduction

Phosphorus is an indispensable element for life, playing a key role in all living organisms.^1,2^ The most important forms of phosphorus in nature are phosphorus oxoanions, especially phosphates: They are essential components of DNA and RNA, and also play a crucial role in cell membranes, providing structural support and facilitating important cellular processes like signaling and transport.^3^ Additionally, adenosine triphosphate (ATP) has a key role in providing energy for biochemical reactions within cells.^4,5^

Despite the ubiquity, biological importance, and economic relevance of phosphorus oxoanions such as ${\mathrm{PO}}_{4}^{3-}$ (ortho-phosphate) and ${\mathrm{HPO}}_{3}^{2-}$ (IUPAC name: phosphonate, commonly called “phosphite”),^6,7^ the literature lacks documentation of ${\mathrm{PO}}_{3}^{3-}$ (herein referred to as “ortho-phosphite”). Strikingly, despite never being reported before, ${\mathrm{PO}}_{3}^{3-}$ is a classical example used in chemistry textbooks and general chemistry exams to teach nomenclature, chemical bonding, and Lewis structures.^8–12^ To the best of our knowledge, characterization of the ${\mathrm{PO}}_{3}^{3-}$ fragment is hitherto limited to polyoxometalate systems, that are templated around a central P^III^O_3_ moiety.^13,14^ The absence of “free” ortho-phosphite in the literature stands in stark contrast to the situation for corresponding heavier ${\mathrm{EO}}_{3}^{3-}$ (E = pnictogen element) trioxoanions such as ortho-arsenite $\left({\mathrm{AsO}}_{3}^{3-}\right)$ , ortho-antimonite $\left({\mathrm{SbO}}_{3}^{3-}\right)$ and ortho-bismuthite $\left({\mathrm{BiO}}_{3}^{3-}\right)$ , which are well-documented,^15–18^ with some examples even occurring in nature (e.g., Reinerite (Zn_3_(AsO_3_)_2_)).^19^ Despite any direct evidence of its existence, ${\mathrm{PO}}_{3}^{3-}$ is suspected to have played a role in prebiotic chemistry: Pasek and co-workers proposed that corrosion of meteoritic schreibersite ((Fe,Ni)_3_P) generates ${\mathrm{PO}}_{3}^{2-}$ radicals which subsequently disproportionate to metaphosphate $\left({\mathrm{PO}}_{3}^{-}\right)$ and ortho-phosphite $\left({\mathrm{PO}}_{3}^{3-}\right)$ , establishing the relevance of the latter in the formation of biogenic compounds.^20^ Its protonated form ${\mathrm{HPO}}_{3}^{2-}$ , stable in water, can undergo mild oxidation to give condensed phosphates (e.g., pyrophosphate, triphosphate), which are phosphorylating agents implicated in the prebiotic formation of polyphosphorylated organic molecules.^6,20–22^

Ortho-phosphite can therefore be construed as a “missing” oxoanion, both regarding the family of phosphorus oxoanions and the heavier ${\mathrm{EO}}_{3}^{3-}$ analogues. This absence is particularly striking given the ubiquity of other phosphorus oxoanions.

Another key relevance of phosphorus oxoanions is their importance in many industrial fields, ranging from fertilizers to flame retardants.^23,24^ All phosphorus compounds we use originate from industrially mined phosphate salts.^25^ However, the legacy process of turning phosphates into reduced phosphorus compounds relies on the production of white phosphorus: a process, which is both energy intensive and environmentally hazardous.^26,27^ Recently, we have turned our attention toward finding synthetic pathways to omit white phosphorus as an intermediate in phosphorus processing.^28–33^ For example, we reported on the synthesis of tetrabutylammonium bis­(trichlorosilyl)­phosphide ([TBA]­[P­(SiCl_3_)_2_]), which we directly prepared from various phosphate sources and trichlorosilane.^28–30^ Studies reported by Quan and co-workers as well as us demonstrate that this phosphide salt can be used as a precursor of a multitude of phosphorus compounds traditionally made from white phosphorus.^28–30,34–37^ Moreover, Weigand and co-workers demonstrated that treatment of phosphates with triflic anhydride and pyridine affords the phosphorylation reagent [(pyridine)_2_PO_2_]­[OTf], which can be further modified to access organophosphinates via redox-neutral pathways.^38,39^ Recently, we have begun to explore mechanochemical reactions involving condensed phosphates, which can act as metaphosphate ( ${\mathrm{PO}}_{3}^{-}$ ) sources (Figure 1b). Ball-milling these phosphates with hydride sources resulted in the formation of phosphite ( ${\mathrm{HPO}}_{3}^{2-}$ ) in a single step (Figure 1c).^31,32^ Very recently, we have expanded the scope of nucleophiles to acetylides and carbides, which we used to synthesize a range of alkynyl phosphonates.^33^

**Figure 1:**
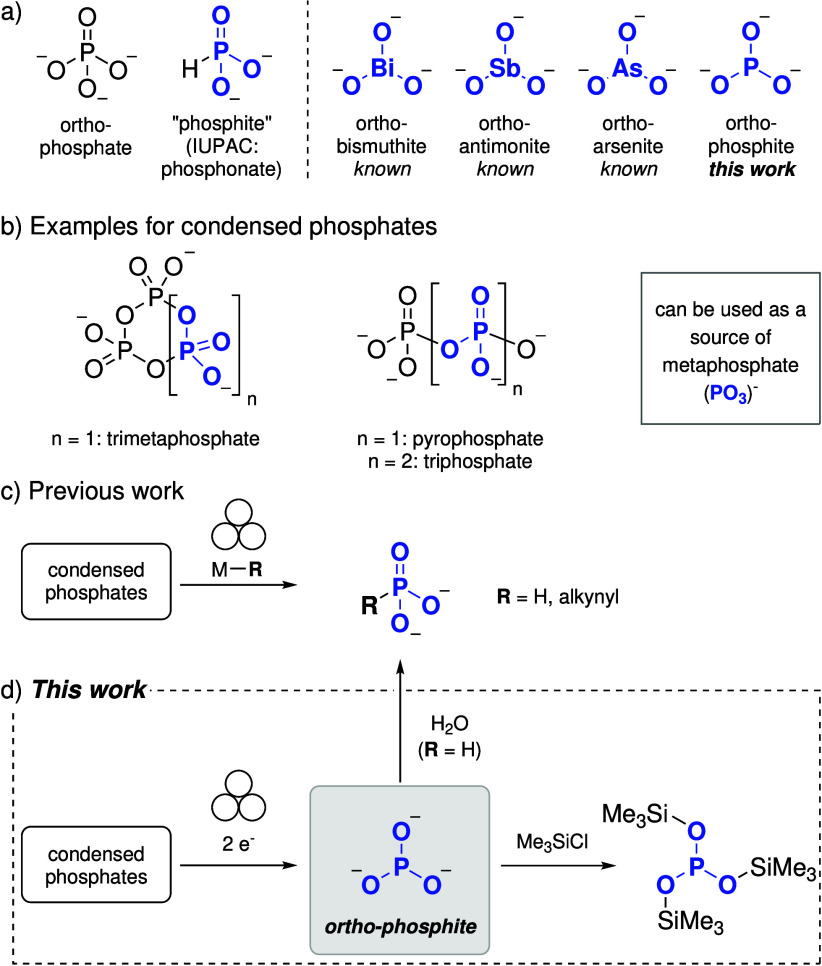
(a) Examples for pnictogen trioxoanions. (b) Examples for condensed phosphates (one of the PO_3_ units highlighted in blue). (c) Previous work on mechanochemical reduction of condensed phosphates. (d) Synthesis of ${\mathrm{PO}}_{3}^{3-}$ containing salts and their reactivity (this work). Counterions are generally omitted for clarity.

These insights motivated us to pursue the synthesis of a ${\mathrm{PO}}_{3}^{3-}$ salt not only from a fundamental standpoint, but also as a potentially useful synthetic intermediate, which could possibly allow for a more atom-economical synthesis of commercially interesting organophosphorus compounds. Targeting to develop a synthetic access to ${\mathrm{PO}}_{3}^{3-}$ , our aim was to reduce condensed phosphates without nucleophilic attack. The envisaged reduction is given in eq 1.

$${{\mathrm{PO}}_{3}}^{-}\overset{{2e}^{-}}{\to }{{\mathrm{PO}}_{3}}^{3-}$$ 1

Herein, we report the first synthesis and characterization of ortho-phosphite $\left({\mathrm{PO}}_{3}^{3-}\right)$ via the mechanochemical reduction of condensed phosphates. Solid-state NMR, Raman, and subsequent reactivity studies confirm its identity and demonstrate its potential as a precursor to industrially relevant organophosphorus compounds. This discovery not only addresses the gap in phosphorus oxoanions but also possibly provides a greener pathway for phosphorus chemistry, circumventing the need for white phosphorus-based processes.

## Results and Discussion

We started by investigating the mechanochemical reaction between condensed phosphates and common reducing agents. In 2018, Jones and co-workers demonstrated that potassium dispersed on potassium iodide (K/KI) is a suitable reductant for the synthesis of magnesium­(I) compounds.^40^ In the following years, the groups of Jones, Evans, and Harder established K/KI as a versatile reducing agent for a range of different low-valent systems.^41–46^ Most notably, Harder and co-workers reported that K/KI can be used as a reductant in mechanochemical synthesis.^44–46^ Therefore, we decided that this might be a suitable reagent for the mechanochemical reduction of condensed phosphates.

To be able to conduct the full synthetic protocol under mechanochemical conditions, we deviated from the standard procedure of K/KI preparation by heating/stirring the mixture under reduced pressure,^40^ and used a ball mill instead (see S.2 for details). A similar approach for the mechanochemical synthesis of Na/NaCl was reported recently by Pearce and co-workers.^47^ Ball-milling potassium chunks with preground KI resulted in formation of a fine, free-flowing deep blue powder, similar to the one previously reported in the literature.^40^ Additionally to the 5% w/w K/KI reported in the literature, we were able to prepare a more concentrated 10% w/w K/KI dispersion as well. Preparation of a 15% w/w K/KI dispersion was attempted, but was unsuccessful due to severe caking of the material.

Next, we focused our attention on choosing a suitable $“{\mathrm{PO}}_{3}^{-}”$ source. Trimetaphosphate $\left(P_{3}O_{9}^{3-}\right)$ is the smallest cyclic phosphate with an O:P ratio of 3. Therefore, it is an attractive starting material, suggesting ideally an atom-economical reduction to ${\mathrm{PO}}_{3}^{3-}$ . In contrast, linear condensed phosphates with the general formula $P_{n}O_{3n+1}^{\left(n+2\right)-}$ have a larger O:P ratio and inherently produce some amount of ${\mathrm{PO}}_{4}^{3-}$ .

Mechanochemical reactions were conducted in a planetary ball mill using 125 mL stainless steel jars and 30 ball bearings (10 mm ϕ). The reaction scope is shown in Table 1. The crude mixtures are completely insoluble in aprotic organic solvents such as tetrahydrofuran, diethyl ether, diglyme, tetramethylethylenediamine or trimethyl phosphate, whereas protic solvents such as methanol or water result in a vigorous reaction and rapid discoloration. Hence, liquid-state NMR analysis of the crude reduction products is limited to the hydrolysis products. ^31^P NMR analysis of aqueous solutions revealed ${\mathrm{PO}}_{4}^{3-}$ and ${\mathrm{HPO}}_{3}^{2-}$ as the major products in most cases, of which the latter is rationalized by hydrolysis of ${\mathrm{PO}}_{3}^{3-}$ (see Figure 2a). Hence, ${\mathrm{PO}}_{3}^{3-}$ yield was determined by quantification of ${\mathrm{HPO}}_{3}^{2-}$ via ^31^P NMR spectroscopy (see S.3.2 in the Supporting Information for more information).

**Figure 2:**
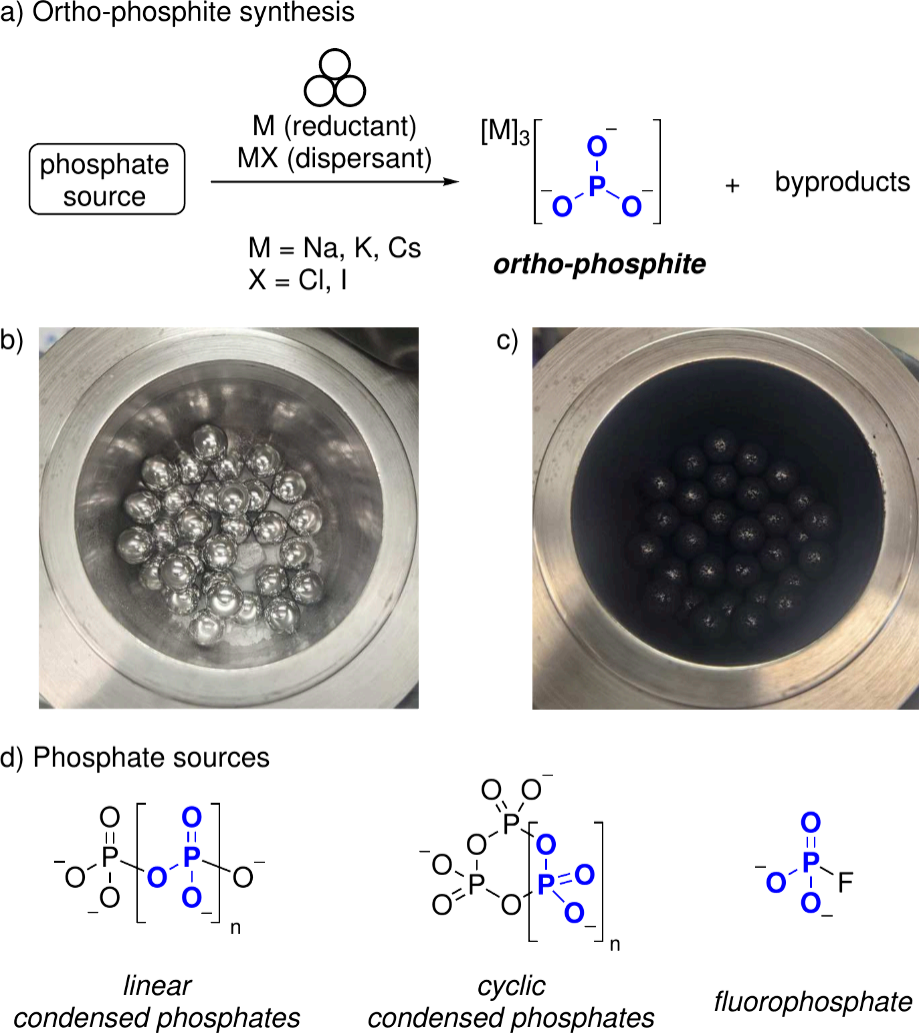
(a) Synthesis of the ${\mathrm{PO}}_{3}^{3-}$ anion. (b) Ball-milling jar charged with Na_3_P_3_O_9_, K and KI before reaction. (c) Ball-milling jar after reaction. (d) Condensed phosphates and fluorophosphate used in this study. Counterions are omitted for clarity.

**Table 1:** Scope of Phosphate Sources, Reductants, Dispersant Loadings, and Reaction Times^a^

phosphate source	red./disp.	red. (equiv)	red./disp. (w/w %)	reaction time (h)	PO_3_^3−^ yield (%)^b^	recovery (%)^b^
Na_3_P_3_O_9_	K/–	6		12	30	87
Na_3_P_3_O_9_	K/KI	6	10	12	32	89
Na_3_P_3_O_9_	K/KI	6	5	12	27	89
Na_5_P_3_O_10_	K/KI	6	10	12	4	94
Na_5_P_3_O_10_	K/KI	6	10	24	13[20]^c^	93
Na_5_P_3_O_10_	K/KI	6	10	36	17[26]^c^	93
(KPO_3_)_n_	K/–	2		24	7	71
(KPO_3_)_n_	K/KI	2	10	24	36	89
Na_2_PO_3_F	K/-	2		12	3	94
Na_2_PO_3_F	K/KI	2	10	12	44	90
Na_3_P_3_O_9_	Na/–	6		12	15	81
Na_3_P_3_O_9_	Na/NaCl	6	10	12	28	83
Na_3_P_3_O_9_	Na/NaCl	6	10	24	27	81
Na_3_P_3_O_9_	Na/NaCl	6	10	36	27	81
Na_3_P_3_O_9_	Cs/–	6		12	5	91
Na_3_P_3_O_9_	Cs/–	6		24	5	90

a Reaction conditions: 450 rpm, 12 h.

b Yields were determined by quantitative ^31^P NMR after hydrolysis; ${\mathrm{PO}}_{3}^{3-}$ yield was determined by amount of generated ${\mathrm{HPO}}_{3}^{2-}$ (see S.3.2 for details).

c The bracketed value of yield is based on the number of reactive PO_3_-units per molecule, accounting for inherent ${\mathrm{PO}}_{4}^{3-}$ generation due to nonideal P:O ratio.

The effect of the dispersant was investigated for the reduction of trisodium trimetaphosphate (Na_3_P_3_O_9_, see Table 1). The K:KI ratio of the K/KI dispersion seems to not significantly affect yield. Moreover, we found that instead of a predispersed K/KI mixture, K and KI can be added directly to the reaction vessel as well, giving similar yield (see Figure 2b for a typical reaction setup). Complete omission of a dispersant gave satisfactory results in many cases, but occasional caking of the potassium metal inside the ball-milling jar led to reproducibility issues. All these reactions, regardless of employing KI as the dispersant or not, gave a black, free-flowing powder (see Figure 2b), which immediately turns white when exposed to air. Other tested dispersants such as silica and graphite did not give any significant amounts of product (see S.4.1). Hence, employment of KI as a dispersant in a 10% w/w K/KI ratio was deemed to be optimal. Additionally, sodium and cesium were investigated as reducing agents, but gave lower yields than potassium, especially in the absence of a dispersant.

Regarding the scope of the condensed phosphates, Na_3_P_3_O_9_ and polymeric metaphosphate (KPO_3_)_n_ gave the best results, as expected because of the ideal O:P ratio of exactly 3 for Na_3_P_3_O_9_ and approximately 3 for (KPO_3_)_n_. The latter species, also called “Kurrol’s salt”, is a high molecular weight form of potassium polyphosphate, which is generally produced by heating KH_2_PO_4_ at 350–500 °C in a rotary kiln.^48,49^ In contrast, the reduction of sodium triphosphate (Na_5_P_3_O_10_) was considerably more sluggish, and gave comparable yield only after 36 h of reaction time. Significant ${\mathrm{PO}}_{4}^{3-}$ generation was observed for all phosphate precursors, even the ones with the ideal O:P ratio. Besides the main species ${\mathrm{PO}}_{3}^{3-}$ and ${\mathrm{PO}}_{4}^{3-}$ , minor amounts of the byproducts [O_3_P–PO_3_]^4–^ (hypophosphate) and [O_3_P–PH_2_]^2–^ were detected and assigned via ^31^P NMR data after hydrolysis.^50,51^ Additionally, PH_3_ was detected after treating the crude mixture with methanol, indicating the presence of phosphide (P^3–^, see S.7 for the PH_3_ detection experiments). The presence of [O_3_P–PO_3_]^4–^ and P^3–^ was further corroborated by silylation of the crude mixture with trimethylsilyl chloride, resulting in the formation of (Me_3_SiO)_3_P–P­(OSiMe_3_)_3_ and P­(SiMe_3_)_3_, which were detected by ^31^P NMR spectroscopy (see S.11.2 for more details). Collectively, these findings indicate the presence of possible competing reduction pathways (see below for a detailed discussion).

After finding the best reaction conditions, the reduction of Na_3_P_3_O_9_ with a 10% w/w K and KI mixture was scaled up to a 500 mL stainless steel jar and 100 ball bearings (ϕ 10 mm), allowing us to produce 18.8 g of the crude mixture in one run (see S.10 for details). The full breakdown of products for this reaction is exemplified in Figure 3.

**Figure 3:**
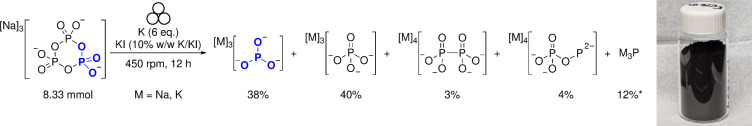
Full product distribution of an exemplary crude mixture. The given example is a scaled up reduction of Na_3_P_3_O_9_ with potassium using KI as a dispersant (10% w/w K/KI). The yield is determined by quantitative solution-state ^31^P NMR analysis of the hydrolysis product. Average oxidation state of phosphorus: + 3.05, P:O atom ratio: 2.97. *Phosphide (M_3_P) yield was calculated as 100% minus the recovered total yield of phosphorus-containing species, as it is converted to gaseous PH_3_ during hydrolysis. Right side: image of the isolated crude mixture in a vial.

In addition to condensed phosphates, Na_2_PO_3_F was investigated as an alternative phosphate precursor and gave a satisfactory result, albeit with significant hypophosphate formation (16% NMR yield). It is also notable that, unlike for Na_3_P_3_O_9_, a dispersant was much more important for the reduction of (KPO_3_)_n_ and Na_2_PO_3_F, which both gave greatly diminished yields when solely milled with potassium.

Spectroscopic evidence of the novel ${\mathrm{PO}}_{3}^{3-}$ species was obtained by solid-state ^31^P NMR analysis of the crude mixtures, showing a resonance at 103 ppm (see Figure 4), which is in the range of organophosphite (P­(OR)_3_) species (for example P­(OPh)_3_: 129 ppm,^52^ P­(OSiMe_3_)_3_: 113 ppm^53^). The signal vanishes when the sample is exposed to ambient air, highlighting the high reactivity of this species (see S.5 for more information). Due to the unknown nature of ${\mathrm{PO}}_{3}^{3-}$ , its ^31^P NMR chemical shift was additionally investigated by computational means. Quantum chemical calculations at the PBE0-D3BJ/def2-TZVP level of theory of a [Na_3_PO_3_]_8_ model cluster predicted a mean ^31^P NMR chemical shift of 105 ppm, which matches our experimental observations (see S.14 for more information).

**Figure 4:**
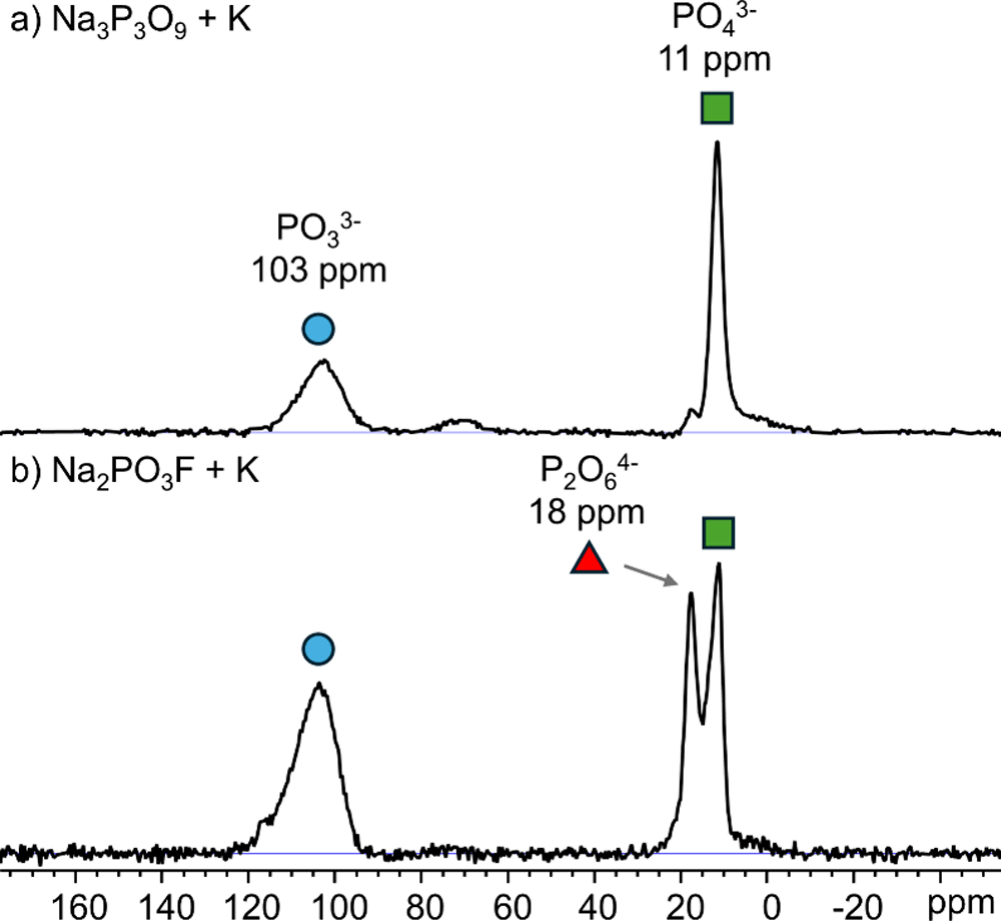
Solid-state ^31^P NMR spectra of exemplary mechanochemical reaction mixtures at 298 K under 20 kHz MAS. (a) Reaction of Na_3_P_3_O_9_ with K. (b) Reaction of Na_2_PO_3_F with K. KI was used as a dispersant in both cases. Note that the particular chemical shifts vary in a range of ± 1 ppm depending on the exact chemical composition of the mixtures. The depicted values for ${\mathrm{PO}}_{3}^{3-}$ and ${\mathrm{PO}}_{4}^{3-}$ are averages. See S.5 for more information about the reaction conditions.

Additionally, to also validate the solid-state ^31^P NMR chemical shift of ${\mathrm{PO}}_{3}^{3-}$ experimentally, an independent generation method of Na_3_PO_3_ and K_3_PO_3_ was developed. Reasoning that ${\mathrm{PO}}_{3}^{3-}$ should also be accessible by deprotonation of ${\mathrm{HPO}}_{3}^{2-}$ , we treated Na_2_HPO_3_ and K_2_HPO_3_ with NaCH_2_SiMe_3_ and KCH_2_Ph, respectively (Figure 5a; see S.4.3 for more information). Solid-state ^31^P NMR analysis of the reaction mixtures showed incomplete conversion of the starting materials, but gratifyingly, the same new resonances at 103 ppm were observed in both cases. Furthermore, after addition of D_2_O to the crude mixtures, ${\mathrm{DPO}}_{3}^{2-}$ was unambiguously detected via solution-state ^31^P NMR analysis as a characteristic 1:1:1 triplet (^1^ *J*_PD_ = 87 Hz) resonating at 2.1 ppm, further validating the successful independent generation of ${\mathrm{PO}}_{3}^{3-}$ . To rule out H/D exchange of ${\mathrm{HPO}}_{3}^{2-}$ as a possible route to ${\mathrm{DPO}}_{3}^{2-}$ , in a separate experiment, Na_2_HPO_3_ was dissolved in D_2_O at pH = 13 and monitored by ^31^P NMR. No deuterium incorporation was observed, confirming that ${\mathrm{DPO}}_{3}^{2-}$ does not arise from simple H/D exchange. Note that preparation of deuterium-labeled phosphite $\left({\mathrm{DPO}}_{3}^{2-}\right)$ via direct H/D exchange of phosphorous acid (H_3_PO_3_) in D_2_O typically requires multiple heating and evaporation cycles (ca. four to six rounds at 65 °C) to reach full deuterium incorporation, as reported in the literature.^54^ Also note that theoretical and thermodynamic analyses indicate that the phosphite dianion $\left({\mathrm{HPO}}_{3}^{2-}\right)$ predominantly adopts the P–H tautomer (**I**, Figure 5b), as the alternative O–H form (**II**) is significantly less stable in aqueous solution.^55^

**Figure 5:**
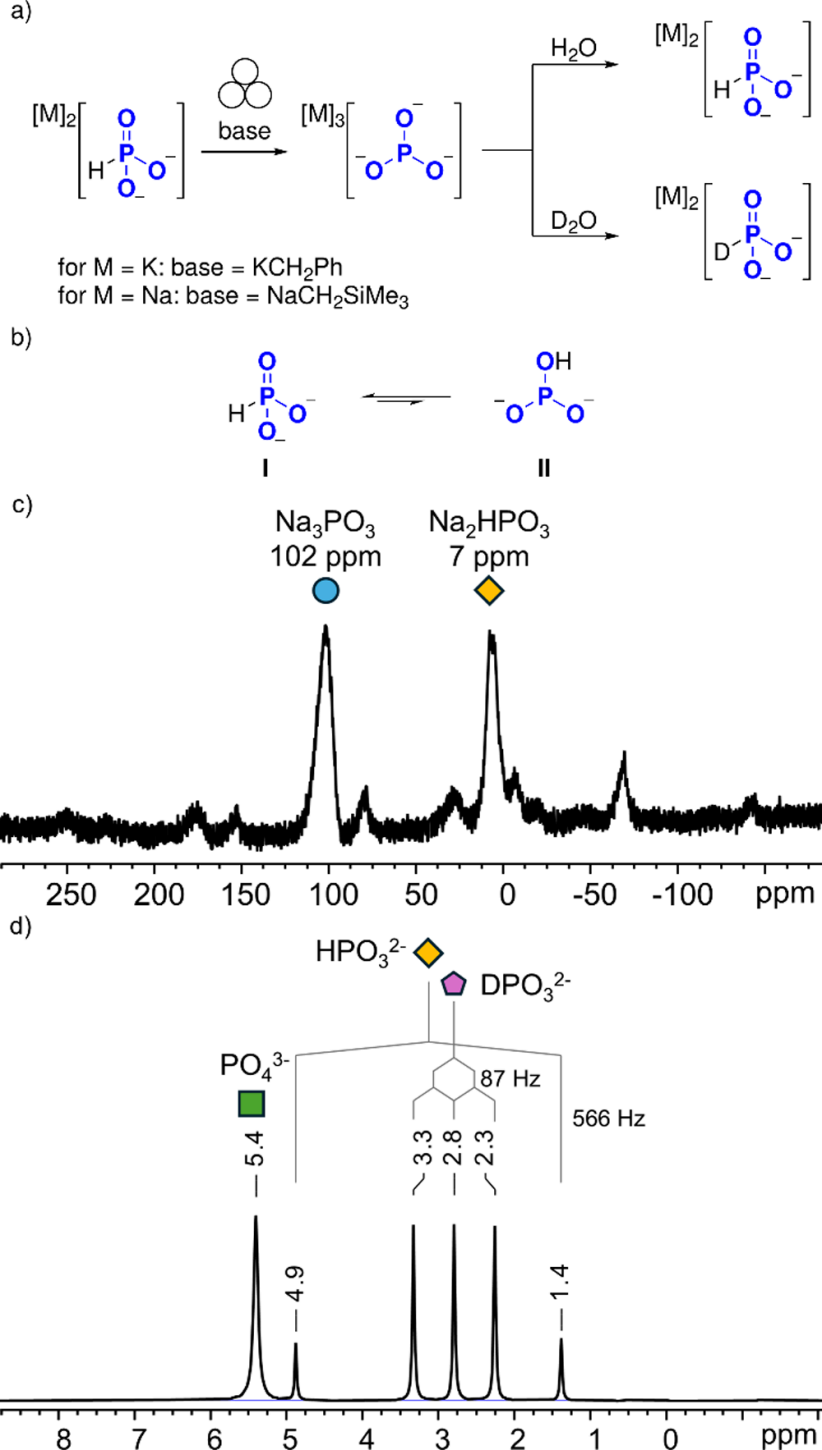
(a) Orthogonal generation of Na_3_PO_3_ and K_3_PO_3_ by deprotonation of Na_2_HPO_3_ with NaCH_2_SiMe_3_ or deprotonation of K_2_HPO_3_ with KCH_2_Ph, respectively. Subsequent treatment of Na_3_PO_3_ and K_3_PO_3_ with H_2_O or D_2_O gives the corresponding (deutero-)­phosphite salt. (b) Tautometic forms **I** and **II** of the phosphite anion. (c) Solid-state ^31^P NMR spectrum related to the orthogonal generation of Na_3_PO_3_ by deprotonation of Na_2_HPO_3_ with NaCH_2_SiMe_3_ after 18 h at 298 K under 20 kHz MAS. (d) Corresponding solution-state ^31^P NMR spectrum after quenching with D_2_O. See S.4.3 for more information.

Further spectroscopic evidence for the presence of ${\mathrm{PO}}_{3}^{3-}$ species was obtained by Raman spectroscopy (Figure 6, see S.6 for more information), which revealed intense peaks at 573 cm^–1^ (shoulder or split mode), 600 cm^–1^ (P–O deformation or asymmetric bending), 887 cm^–1^ (P–O symmetric stretching), and additional peaks at 250 cm^–1^, consistent with the computed spectra of Na_3_PO_3_ and K_3_PO_3_ (see S.13.3). Raman bands characteristic of ${\mathrm{PO}}_{4}^{3-}$ were also detected at 410, 573, 923, and 1030 cm^–1^, in agreement with the reference spectrum for K_3_PO_4_ (Figure S.47a) and Na_3_PO_4_.^56^ Notably, increasing the laser power led to an enhancement of ${\mathrm{PO}}_{4}^{3-}$ peaks and a concurrent loss of ${\mathrm{PO}}_{3}^{3-}$ peaks (Figure S.48), indicating the thermal sensitivity of ${\mathrm{PO}}_{3}^{3-}$ and potential conversion to ${\mathrm{PO}}_{4}^{3-}$ . Peaks at 315 and 493 cm^–1^ may be attributed to additional phosphate species such as $P_{2}O_{6}^{4-}$ .^57^ Additionally, no $P_{3}O_{9}^{3-}$ -related peaks were detected (reference spectrum in Figure S.47b), confirming the complete conversion of the starting material, consistent with NMR data (see Figure S.8).

**Figure 6:**
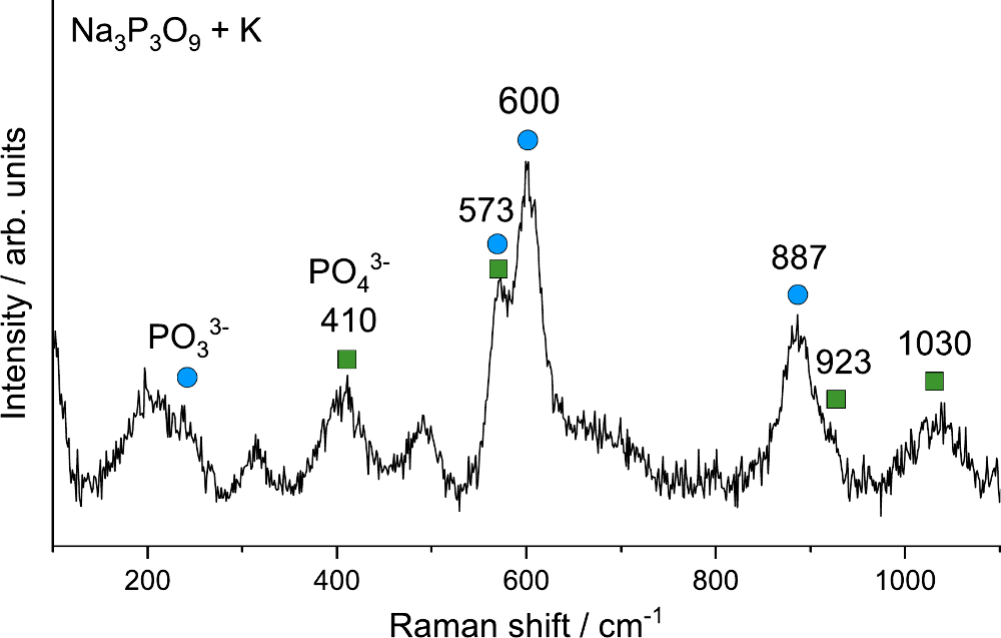
Raman spectrum of an exemplary mechanochemical reaction mixture (Na_3_P_3_O_9_ + K, no dispersant). The laser power was set to 4.3 mW to avoid sample damage (see S.6 for more information). Blue circles indicate peaks assigned to ${\mathrm{PO}}_{3}^{3-}$ -containing salts and green squares indicate peaks assigned to ${\mathrm{PO}}_{4}^{3-}$ -containing salts.

Based on the Raman and ^31^P NMR spectroscopic data of the hydrolysis products, crude solids, and subsequent reaction mixtures, we propose a pathway for the formation of the ${\mathrm{PO}}_{3}^{3-}$ species and its associated byproducts. An overview of the proposed pathways is shown in Figure 7. For simplicity, metaphosphate species are discussed as the monomeric unit ${\mathrm{PO}}_{3}^{-}$ . We suggest a two-step reduction of ${\mathrm{PO}}_{3}^{-}$ to ${\mathrm{PO}}_{3}^{3-}$ , proceeding through an intermediate radical species $\left({\mathrm{PO}}_{3}^{2-}\right)$ . This radical could potentially dimerize via either P–P or P–O bond formation, leading to two distinct isomeric products. The P–P bonded isomer, [O_3_P–PO_3_]^4–^ (hypophosphate), was observed in both solid-state and aqueous solution-phase ^31^P NMR spectra of the crude mixture. This reactivity is further corroborated by literature on the potential relevance of ${\mathrm{PO}}_{3}^{2-}$ radicals in prebiotic chemistry, where ${\mathrm{PO}}_{3}^{2-}$ is postulated as an intermediate in the generation of [O_3_P–PO_3_]^4–^ in the hydrolysis of iron phosphide (FeP) or its mineral equivalent schreibersite ((Fe,Ni)_3_P).^6,58^ While this species could alternatively form through the reaction of ${\mathrm{PO}}_{3}^{3-}$ with ${\mathrm{PO}}_{3}^{-}$ , this pathway appears less likely given the high oxophilicity of phosphorus, which would favor P–O over P–P bond formation. The formation of both ${\mathrm{PO}}_{4}^{3-}$ and highly reduced species such as [O_3_P–O–PH_2_]^2–^ can be rationalized by the disproportionation of intermediate P­(III) species. We propose that this process arises from a cascade of reactions involving mixed-valent phosphate anhydrides (P­(III)/P­(V)). Further disproportionation of these intermediates likely leads to the generation of more reduced phosphide species within the reaction mixture, with the formation of ${\mathrm{PO}}_{4}^{3-}$ acting as a thermodynamic driving force. This hypothesis is supported by the detection of both [O_3_P–O–PH_2_]^2–^ and PH_3_ following hydrolysis/methanolysis and by previously reported data on the disproportionation behavior of species such as H_3_PO_3_, ${\mathrm{HPO}}_{3}^{2-}$ , and $H_{2}{\mathrm{PO}}_{2}^{-}$ .^59–61^

**Figure 7:**
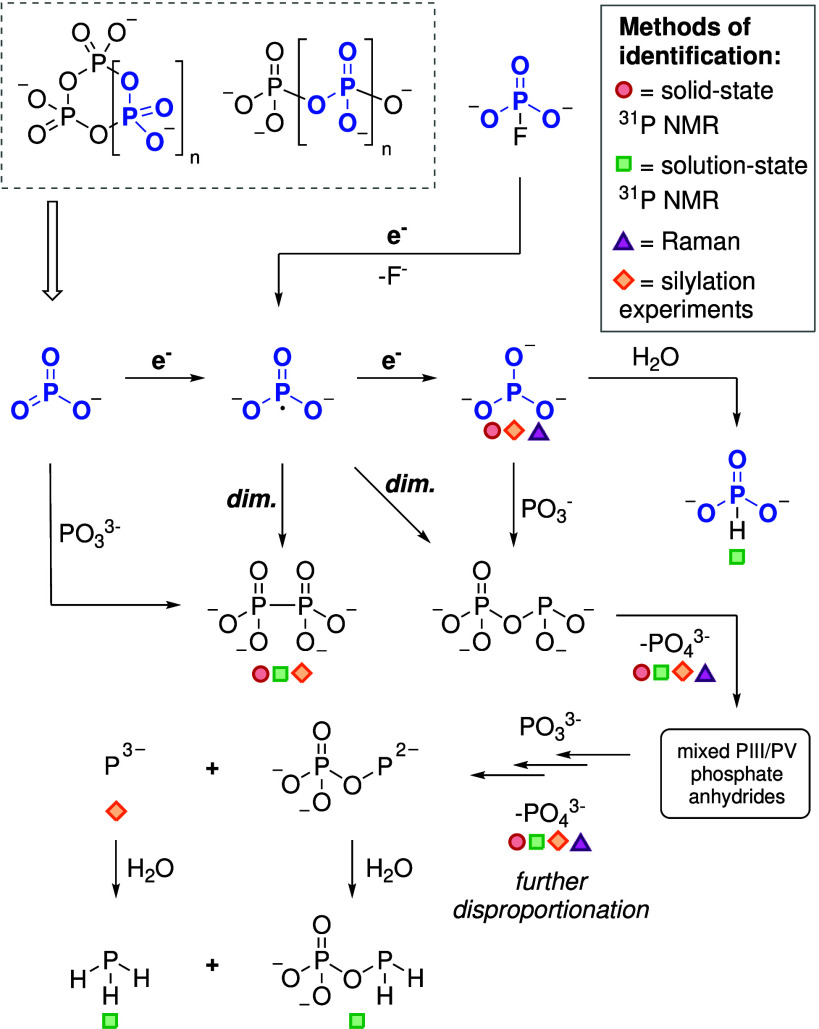
Proposed pathways in the reduction of condensed phosphates and fluorophosphate. For simplicity, condensed phosphates are regarded as metaphosphate $\left({\mathrm{PO}}_{3}^{-}\right)$ units. Spectroscopically detected species are marked accordingly.

To further substantiate the disproportionation mechanism and exclude oxidation by external factors such as oxygen contamination, we performed elemental oxygen accounting via quantitative ^31^P NMR experiments and calculated the average phosphorus oxidation states for representative reactions, taking into account the formation of PH_3_ as a byproduct (see S.9 for more information). The results revealed a consistent P:O ratio of 1:3 and an average phosphorus oxidation state of +3, aligning well with the proposed origin of the observed byproducts. Additionally, ICP-OES analyses of a representative hydrolyzed crude mixture were performed, further corroborating the P:Na:K element ratios, confirming a P-loss of ca. 10%, presumably as PH_3_, as discussed above (see S.8 for more information).

With alkali metal salts of the ${\mathrm{PO}}_{3}^{3-}$ species in hand, we were eager to explore their reactivity, especially as precursors to organophosphorus compounds. Since the solubility properties of the crude ball-milling mixture precluded any purification, we resorted to using the mixture directly in subsequent reactions (see Figure 8). First, we sought to utilize our discovery that hydrolysis of ${\mathrm{PO}}_{3}^{3-}$ seems to quantitatively generate ${\mathrm{HPO}}_{3}^{2-}$ . Traditionally synthesized from PCl_3_,^48^ this compound is used in battery materials^62–64^ and in agriculture as fertilizer and fungicide.^7,65,66^ Therefore, we focused on isolating this phosphite generated in our synthesis. Its separation from phosphates in the crude mixture was accomplished by precipitating ortho-phosphate as struvite (NH_4_MgPO_4_· 6H_2_O), taking advantage of this mineral’s low solubility. This process yielded phosphite in a 66% isolated yield which was recovered as barium phosphite (BaHPO_3_· H_2_O),^67^ as described in detail in S.11.1. We selected the Ba^2+^ salt due to its low aqueous solubility, which facilitates clean isolation from soluble byproducts.

**Figure 8:**
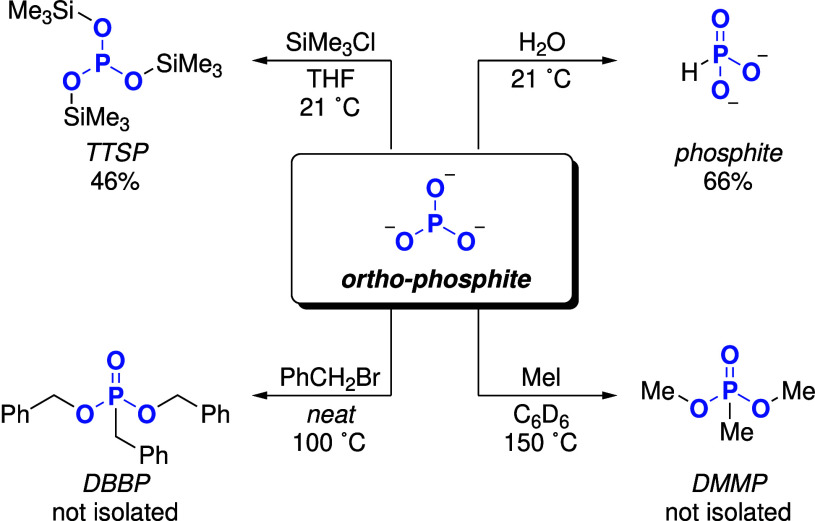
Reactivity of ${\mathrm{PO}}_{3}^{3-}$ containing salts synthesized in this study. Percentage refers to isolated yield. Counterions are omitted for clarity.

Treatment of the crude ${\mathrm{PO}}_{3}^{3-}$ containing material with trimethylsilyl chloride resulted in formation of P­(OSiMe_3_)_3_ (tris­(trimethylsilyl)­phosphite, TTSP), which was isolated by vacuum distillation. TTSP is a precursor used in the synthesis of phosphonates via the Michaelis–Arbuzov reaction,^68^ and thus is an attractive starting material for the synthesis of organophosphorus compounds. Moreover, it is used as a ligand in transition metal catalysis^69^ and as a battery electrolyte.^70^ Additionally, in an attempt to synthesize the heavier analogue P­(OGeMe_3_)_3_, the crude ${\mathrm{PO}}_{3}^{3-}$ containing material was treated with trimethylgermyl chloride and the reaction mixture was analyzed via ^31^P NMR spectroscopy. In contrast to the silylation result, no signal was detected that could be assigned to P­(OGeMe_3_)_3_. Instead, among other resonances, a signal with a chemical shift of 31.2 ppm with a characteristic splitting pattern (*J*_PH_ = 8 Hz) was detected. This signal was tentatively assigned to the corresponding phosphonate OP­(OGeR_3_)_2_(GeR_3_) (see S.69 for more information), which is consistent with the lower oxophilicity of germanium compared to silicon, rendering this isomer more favorable. Note that we could find no literature examples of a compound with the general formula OP­(OGeR_3_)_2_(GeR_3_).^71^ Preliminary studies on the alkylation of the crude ${\mathrm{PO}}_{3}^{3-}$ containing material with alkyl halides indicate the formation of the corresponding alkyl phosphonates: Treatment of the crude ${\mathrm{PO}}_{3}^{3-}$ containing material with methyl iodide and benzyl bromide resulted in the formation of dimethyl methylphosphonate (DMMP) and dibenzyl benzylphosphonate (DBBP), respectively (see S.12 for more information). DMMP (which is conventionally prepared from white phosphorus) is particularly interesting, because of its industrial relevance as a flame retardant.^23^ However, we noticed that the yield of these alkylations was low (NMR-yield: 28% (DMMP) and 32% (DBBP)) and a purification of the compounds was not successful. Hence, they were not isolated. Additionally, treatment of the crude ${\mathrm{PO}}_{3}^{3-}$ containing material with ethyl bromoacetate was attempted, but resulted in a complex reaction mixture after heating at 150 °C for 16 h. No reaction at all was observed after treating the crude ${\mathrm{PO}}_{3}^{3-}$ containing material with phenyl chloride (up to 100 °C for 1 day).

## Conclusion

We found synthetic access to the so-far overlooked phosphorus oxoanion “ortho-phosphite” ${\mathrm{PO}}_{3}^{3-}$ by mechanochemical reduction of condensed phosphates. Evidence for the formation of this species was achieved by solid-state ^31^P NMR spectroscopy, Raman spectroscopy, and its characteristic subsequent reactivity. Ortho-phosphite is not only interesting from a fundamental chemistry perspective, as we found that it can be used for the synthesis of industrially relevant chemicals, such as phosphite salts, TTSP or phosphonates, all traditionally synthesized from white phosphorus. As mechanochemistry is on the rise in industrial chemistry,^72–75^ we look forward to further developing the synthesis of ortho-phosphite as an attractive alternative to the production of white phosphorus en route to valuable organophosphorus compounds. Additionally, further reactivity studies on this novel species are ongoing in our laboratories.

## Supplementary Material



## Notes

Experimental details, characterization data, and photographs of the synthesized materials (PDF)

## §

Deceased on November 17, 2023.
