# Supplementary material for: Ortho-phosphite (PO33−) : Mechanochemical Synthesis of a Missing Oxoanion and Precursor to Value-Added Organophosphorus Compounds
Source: ACS Cent Sci. 2025 Nov 26;12(1):40–8. doi: 10.1021/acscentsci.5c01595 (PMC12856655; doi:10.1021/acscentsci.5c01595)
Supplement: Supplementary file 1 [file oc5c01595_si_001.pdf]

Ortho-phosphite (PO_3^{3-}): Mechanochemical Synthesis of a Missing Oxoanion and Precursor to Value-Added Organophosphorus Compounds

Pawel Löwe,[†,¶] Rachid Taakili,[†,¶] Tiansi Xin,[†,§] Hritwik Haldar,[†] Antonia Herzog,[‡] Yang Shao-Horn,[†,‡] and Christopher C. Cummins[*,†]

†*Department of Chemistry, Massachusetts Institute of Technology, Cambridge, Massachusetts, 02139, United States*

‡*Research Laboratory of Electronics, Massachusetts Institute of Technology, Cambridge, Massachusetts, 02139, United States*

¶*Authors contributed equally*

§*T.X. Deceased on November 17, 2023*

E-mail: ccummins@mit.edu

Table of Contents

Table of Contents **S.2**

S.1 General Information **S.4**

S.2 Preparation of the K/KI Dispersions **S.5**

S.3 General Procedure for the Synthesis of PO_3^{3-} Salts **S.7**
- S.3.1 General Synthetic Procedure . . . S.7
- S.3.2 General Procedure for Determining the Yield . . . S.7
- S.3.3 Example Procedure: Optimized Reaction Conditions . . . S.7

S.4 Reaction Screening **S.8**
- S.4.1 Dispersant Screening . . . S.8
- S.4.2 ^{31}P NMR Spectra of the Mechanochemical Reduction Reactions . . . S.9
  - S.4.2.1 Non-quantitative ^{31}P NMR spectra . . . S.9
  - S.4.2.2 Quantitative ^{31}P NMR spectra . . . S.13
- S.4.3 Orthogonal Synthesis of Ortho-phosphite . . . S.22
  - S.4.3.1 From Na_2HPO_3 and [(Trimethylsilyl)methyl]sodium ($NaCH_2SiMe_3$) S.22
  - S.4.3.2 From Na_2HPO_3 and Benzyl Potassium (BnK) . . . S.27
  - S.4.3.3 From K_2HPO_3 and Benzyl Potassium (BnK) . . . S.30
- S.4.4 Attempted Isotopic Exchange of Hydrogen with Deuterium in Na_2HPO_3 . S.32

S.5 ^{31}P Solid-state NMR Analyses of the Crude Ball-milling Mixtures **S.32**
- S.5.1 Reduction of $Na_3P_3O_9$ with K (Without Dispersant) . . . S.32
- S.5.2 Reduction of $Na_3P_3O_9$ with K and KI . . . S.36
- S.5.3 Exposing PO_3^{3-} Containing Crude Material to Air . . . S.36
- S.5.4 Reduction of Na_2PO_3F with K and KI . . . S.38

S.6 Raman Analyses of the Crude Ball-milling Mixtures **S.39**

S.7 Detection of PH_3 after PO_3^{3-} Containing Crude Material Hydrolysis/Methanolysis **S.42**

S.8 ICP-OES Analysis of Crude Material after Hydrolysis **S.44**

S.9 Assessment of Oxygen Balance and Phosphorus Redox States **S.45**

S.10 Scale-up of the PO_3^{3-} Salt Synthesis **S.46**

S.11 Reactivity of the Crude PO_3^{3-} Salt **S.49**
- S.11.1 Synthesis of Phosphite (HPO_3^{2-}) Salts . . . S.49
- S.11.2 Synthesis of $P(OSiMe_3)_3$ . . . S.51
  - S.11.2.1 In THF . . . S.51
  - S.11.2.2 Without Solvent . . . S.53

S.12 Preliminary Reactivity Studies **S.54**
S.12.1 Generation of Dimethyl Methylphosphonate (DMMP) S.54
S.12.2 Generation of Dibenzyl Benzylphosphonate (DBBP) S.56
S.12.3 Reaction of the ${PO_3}^{3-}$-Containing Crude Mixture with $GeMe_3Cl$ S.58

S.13 Computed Raman Spectra **S.60**
S.13.1 General S.60
S.13.2 Optimization of the Na_3PO_3 and K_3PO_3 Model Structures S.60
S.13.3 Raman Spectra of the Na_3PO_3 and K_3PO_3 Model Structures S.63
S.13.4 XYZ-files S.64

S.14 ^{31}P NMR Shielding Calculations **S.68**
S.14.1 XYZ-files S.69

S.15 References **S.71**

S.1 General Information

Except as otherwise noted, all manipulations were performed in a Vacuum Atmospheres model MO-40M glovebox or a Vacuum Atmospheres GENESIS model under an inert atmosphere of purified N_2. All solvents were obtained anhydrous and oxygen-free by bubble degassing (Ar), purified by passage through columns of alumina using a solvent purification system (Pure Process Technology, Nashua, NH),[1] and stored over 3 Å or 4 Å molecular sieves.[2] Deuterated solvents were degassed and stored over 4 Å molecular sieves for at least 48 h prior to use (except D_2O). Celite® (EM Science), 3 Å molecular sieves and 4 Å molecular sieves were dried by heating above 200 °C under dynamic vacuum for at least 48 h prior to use. All glassware was dried in an oven for at least two hours at temperatures greater than 120 °C.

Sodium trimetaphosphate ($Na_3P_3O_9$, ≥98%, Sigma-Aldrich), sodium fluorophosphate (Na_2PO_3F, 95%, Sigma-Aldrich), sodium triphosphate ($Na_5P_3O_{10}$, ≥98%, Sigma-Aldrich), potassium pyrophosphate ($K_4P_2O_7$, ≥97%, Sigma-Aldrich), potassium metaphosphate ($(KPO_3)_n$, 98%, Strem Chemicals), silica (40–60 μm, 60 Å, VWR) and graphite flakes (natural, −10 mesh, 99.9% metals basis, Thermo Scientific), and sodium chloride were dried under dynamic vacuum at least 200 °C for at least 24 h and stored in the glovebox. Potassium iodide for K/KI was ground using mortar and pestle, and dried at 250 °C under dynamic vacuum overnight. For the optimized procedure without pre-dispersing K/KI, anhydrous potassium iodide (≥99%, Sigma-Aldrich) was used as received. Potassium was obtained as chunks in mineral oil (97%, Thermo Scientific). These chunks were cut into smaller pieces, brought into the glovebox, washed with pentane or hexane, and then stored until use. Liquid reactants were obtained from common commercial sources and dried over 4 Å molecular sieves (except trimethylsilyl chloride) and stored in the glovebox. All other reactants were obtained from common commercial sources and used as received.

Liquid-state NMR spectra were obtained on a Bruker Avance-III HD Nanobay spectrometer operating at 400.09 MHz equipped with a 5 mm liquid-nitrogen cooled Prodigy broad band observe cryoprobe. Solid-state NMR spectra were obtained on a Bruker Avance Neo spectrometer operating at 500.18 MHz equipped with a 3.2 mm HX solids probe. ^{1}H NMR spectra were referenced internally to residual solvent signals.[3] ^{31}P NMR spectra were externally referenced to 85% H_3PO_4 (0 ppm).

Mechanochemical reactions were performed in a 125 mL stainless steel milling jar (Retsch, part number: 01.462.0148) equipped with a safety closure device (Retsch, part number: 22.867.0007) or a 500 mL stainless steel milling jar (Retsch, part number: 01.462.0520) equipped with a safety closure device (Retsch, part number: 22.867.0012). The jars were filled with 10 mm stainless steel balls (125 mL jar: 30 balls; 500 mL jar: 100 balls). Retsch PM 100 planetary ball mill was used for milling (Retsch, part number 20.540.0001).

S.2 Preparation of the K/KI Dispersions

The K/KI was titrated prior to use. Titration procedure: About 300 mg of the solid was weighed accurately and quenched with dry methanol (1 mL). The resulting suspension was brought out of the glovebox and dissolved in water (9 mL). The solution was titrated with HCl solution of known concentration to neutral pH. This procedure was repeated twice and the potassium weight percent was calculated.

10 w/w%: In the glovebox, the 125 mL milling jar was charged with potassium iodide (20 g), potassium metal (2.22 g, freshly cut into small pieces) and thirty 10 mm balls. The jar was sealed properly and brought out. The mixture was ball milled at 200 rpm for 5 h (cooling break for 30 min every 30 min) and brought back in. The jar was opened in the glovebox. Severe aggregation of the solid (aka caking) was observed. The aggregated solid was pulverized with a spatula, and another portion of potassium iodide (18.8 g) was added. The jar was sealed again, brought out and ball milled at 400 rpm for 25 min. The jar was brought in and opened. The aggregated solid was pulverized with a spatula, and another portion of potassium metal (2.00 g, freshly cut into small pieces) was added. The jar was sealed again, brought out and ball milled at 200 rpm for 10 h (cooling break for 1 h every 2 h). The jar was brought in and opened. The aggregated solid was pulverized with a spatula. The jar was sealed again, brought out and ball milled at 200 rpm for 15 min. The jar was brought in and opened. There was finally no aggregated solid. The blue powder was transferred into a vial.

Titration result: 9.8 (±0.1) w/w%.

5 w/w%: In the glovebox, the milling jar was charged with KI (20.3 g), K metal (1.07 g, freshly cut into small pieces) and thirty 10 mm balls. The jar was sealed properly and brought out. The mixture was ball milled at 200 rpm for 10 min then 400 rpm for 10 min and brought back in. The jar was opened in the glovebox. The aggregated solid was pulverized with a spatula, and the jar was sealed again, brought out and ball milled at 200 rpm for 10 min. The jar was brought in and opened. The blue powder was transferred into a vial.

Titration result: 5.0 (±0.1) w/w%.

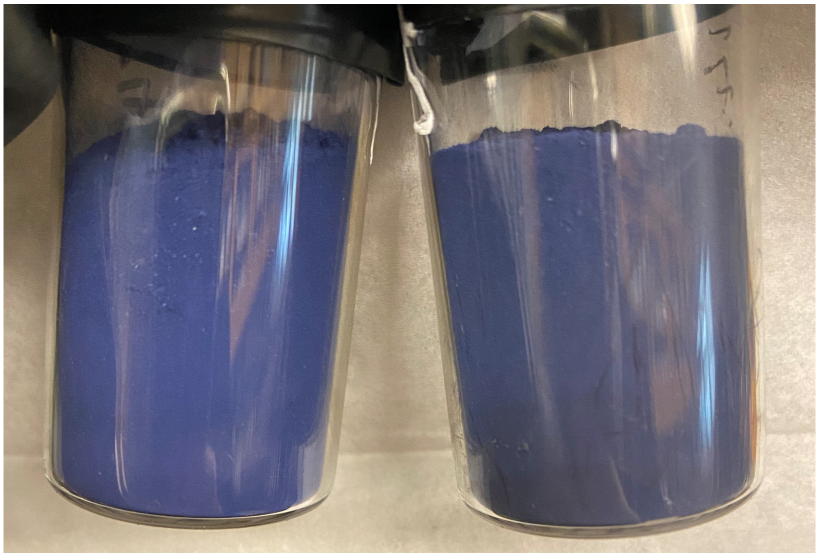

Figure S.1: Isolated K/KI. Left side: 10 w/w%; right side: 5 w/w%.

S.3 General Procedure for the Synthesis of PO_3^{3-} Salts

S.3.1 General Synthetic Procedure

Inside the glovebox, the **condensed phosphate**, **the reducing agent**, the **dispersant** and thirty stainless steel balls (∅ 10 mm) were added into a 125 mL ball-milling jar. The jar was sealed properly and brought out. The mixture was ball milled at the set **speed** and **duration**. Cooling breaks of 30 min were undertaken after each 30 min milling interval. After each interval, the milling direction was reversed. When the desired milling time was reached, the jar was brought into the glovebox and opened. The reaction mixture was isolated in a vial. (*Warning: The obtained crude mixture is pyrophoric. Extreme caution and proper protecting equipment is advised.*)

S.3.2 General Procedure for Determining the Yield

To determine the amount of PO_3^{3-} generated, the crude mixture was hydrolyzed: A portion of the crude mixture was transferred into another vial, brought out and cooled in an ice bath. Deionized water (ca. 5 ml) was carefully added to the material. (*Warning: The reaction may be vigorous. Traces of phosphine gas may form during the hydrolysis. Proper protecting equipment and working inside of a fume hood is strictly required.*) 250 mg $NaHCO_3$ was added to neutralize the solution. Then, a known amount of $OP(OEt)_3$ was added as an internal standard. The solution was analyzed by ^{31}P NMR spectroscopy. For the NMR measurements, a scan delay of 40 s was used for a reliable integration, as determined in a previous publication.[4] The amount of hydrolysis product HPO_3^{2-} was determined to calculate the yield of PO_3^{3-}.

S.3.3 Example Procedure: Optimized Reaction Conditions

Inside the glovebox, $Na_3P_3O_9$ (511 mg, 1.57 mmol, 1.00 eq.), potassium (391 mg, 10.0 mmol, 6.00 eq., freshly cut single chunk), KI (1.56 g), and thirty stainless steel balls (∅ 10 mm) were added into a 125 mL ball-milling jar. The jar was sealed properly and brought out. The mixture was ball milled at 200 rpm for 5 min, then 450 rpm for 12 h (with cooling breaks of 30 min every 1 h and direction change every cycle, total process time 24 h.). When the desired grinding time was reached, the jar was brought into the glovebox and opened. The reaction mixture was isolated in a vial.

S.4 Reaction Screening

S.4.1 Dispersant Screening

For the screening, the general procedure described in subsection S.3 was followed with the following parameters:
Phosphate: $Na_3P_3O_9$ (511 mg, 1.57 mmol, 1.00 eq.)
Reducing agent: potassium (391 mg, 10.0 mmol, 6.00 eq., freshly cut single chunk)
Milling cycles: 200 rpm for 5 min, then 450 rpm for 12 h (with cooling breaks of 30 min every 1 h and direction change every cycle, total process time 24 h.)

Table S.1: Dispersant screening scope.

dispersant	reductant/dispersant (w/w %)	yield PO_3^{3-} (%)
none	–	30
potassium iodide	10	32
potassium iodide	5	27
silica	10	*none*
graphite[a]	10	*traces*

[a]Reductant and dispersant were pre-dispersed in a separate step (200 rpm for 5 min then 450 rpm for 3 h (with cooling breaks of 30 min every 1 h and direction change every cycle, total process time 6 h).

S.4.2 ^{31}P NMR Spectra of the Mechanochemical Reduction Reactions

S.4.2.1 Non-quantitative ^{31}P NMR spectra

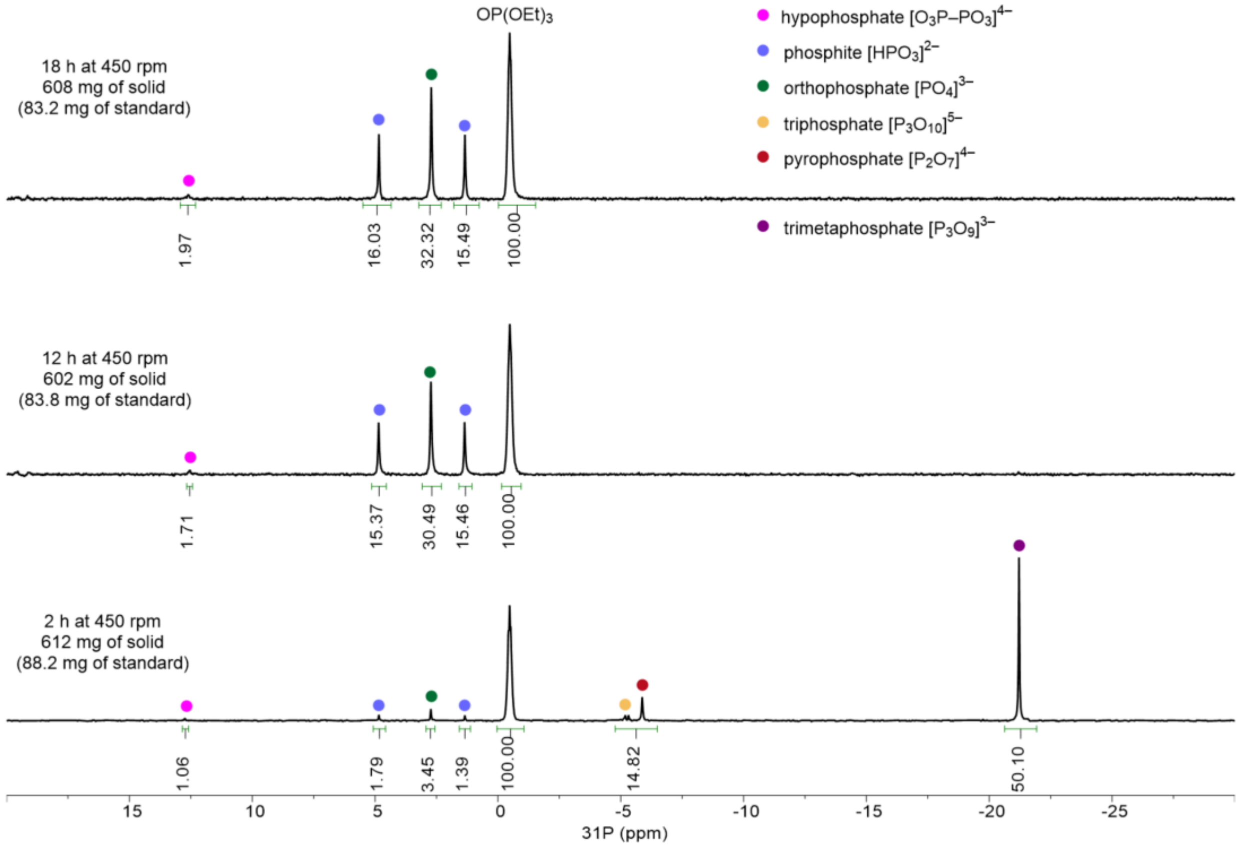

Figure S.2: ^{31}P NMR spectrum (162 MHz, H_2O/D_2O, $d1$ = 30 s, 298 K) of the reaction mixture of $Na_3P_3O_9$ (1.00 mmol) with K/KI (pre-dispersed, 5% w/w, 6.00 mmol) after the given time. $OP(OEt)_3$ was added as internal standard.

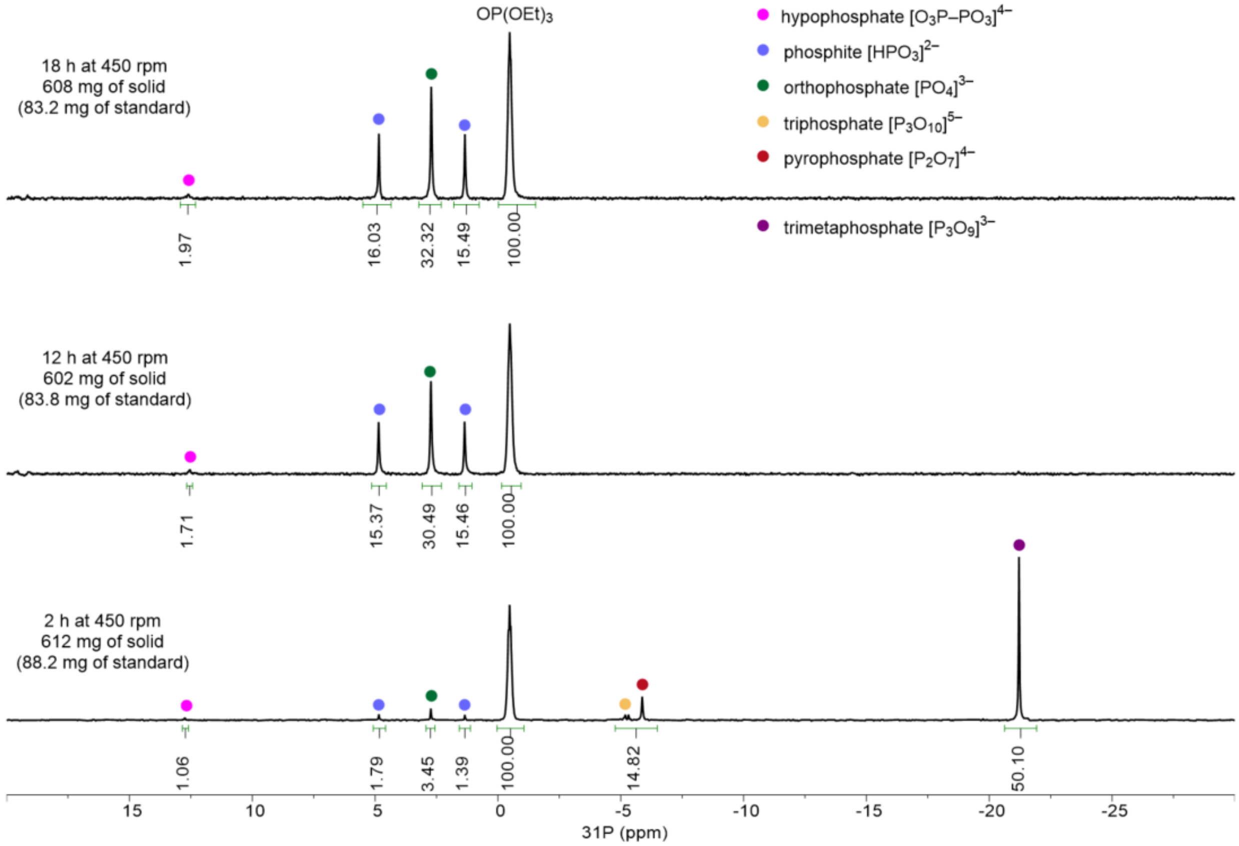

Figure S.3: ^{31}P NMR spectrum (162 MHz, H_2O/D_2O, $d1$ = 30 s, 298 K) of the reaction mixture of $Na_3P_3O_9$ (1.67 mmol) with K/KI (pre-dispersed, 10% w/w, 10.0 mmol) after the given time. $OP(OEt)_3$ was added as internal standard.

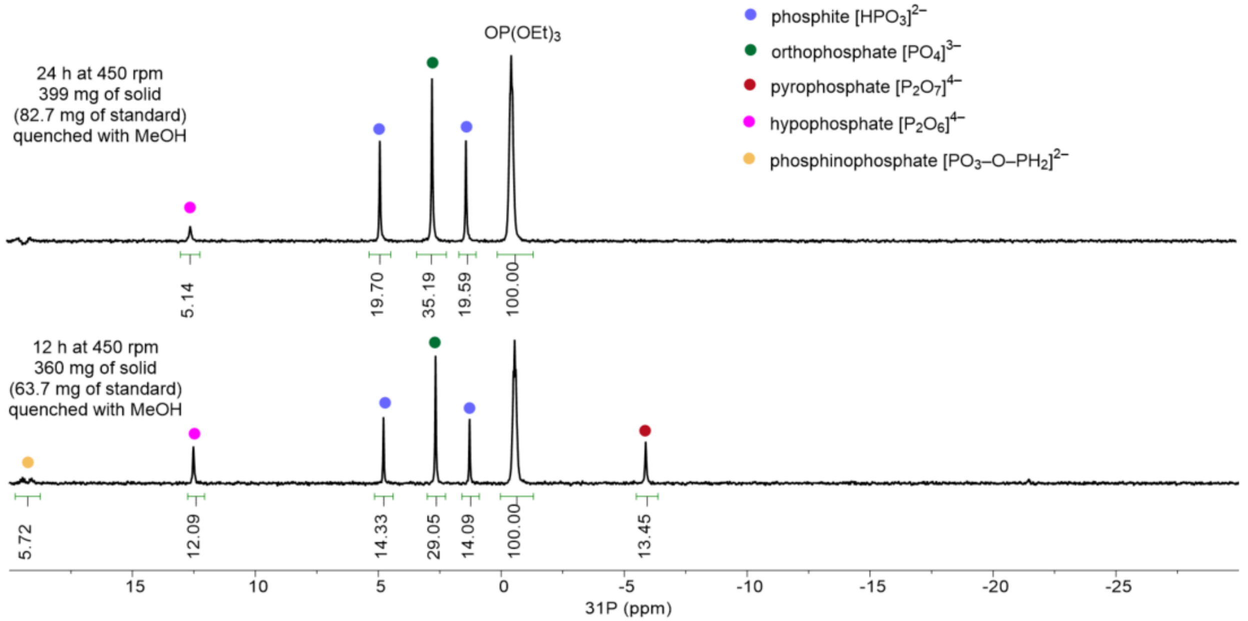

Figure S.4: ^{31}P NMR spectrum (162 MHz, H_2O/D_2O, $d1$ = 30 s, 298 K) of the reaction mixture of $(KPO_3)_n$ (5.00 mmol "KPO_3^-") with K/KI (pre-dispersed, 10% w/w, 10.0 mmol) after the given time. $OP(OEt)_3$ was added as internal standard.

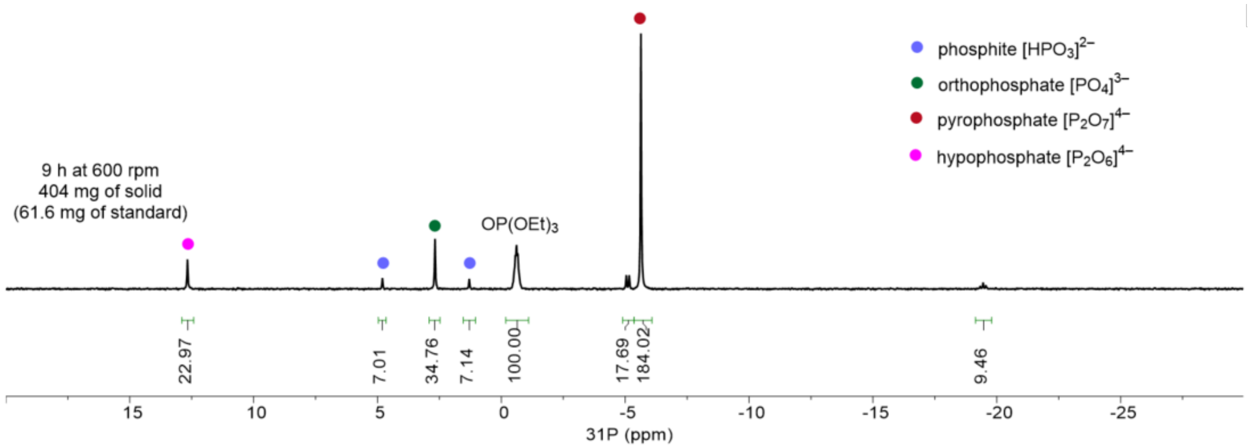

Figure S.5: ^{31}P NMR spectrum (162 MHz, H_2O/D_2O, $d1$ = 30 s, 298 K) of the reaction mixture of $Na_5P_3O_{10}$ (4.50 mmol) with K/KI (pre-dispersed, 10% w/w, 9.00 mmol) after 9 h at 600 rpm. $OP(OEt)_3$ was added as internal standard.

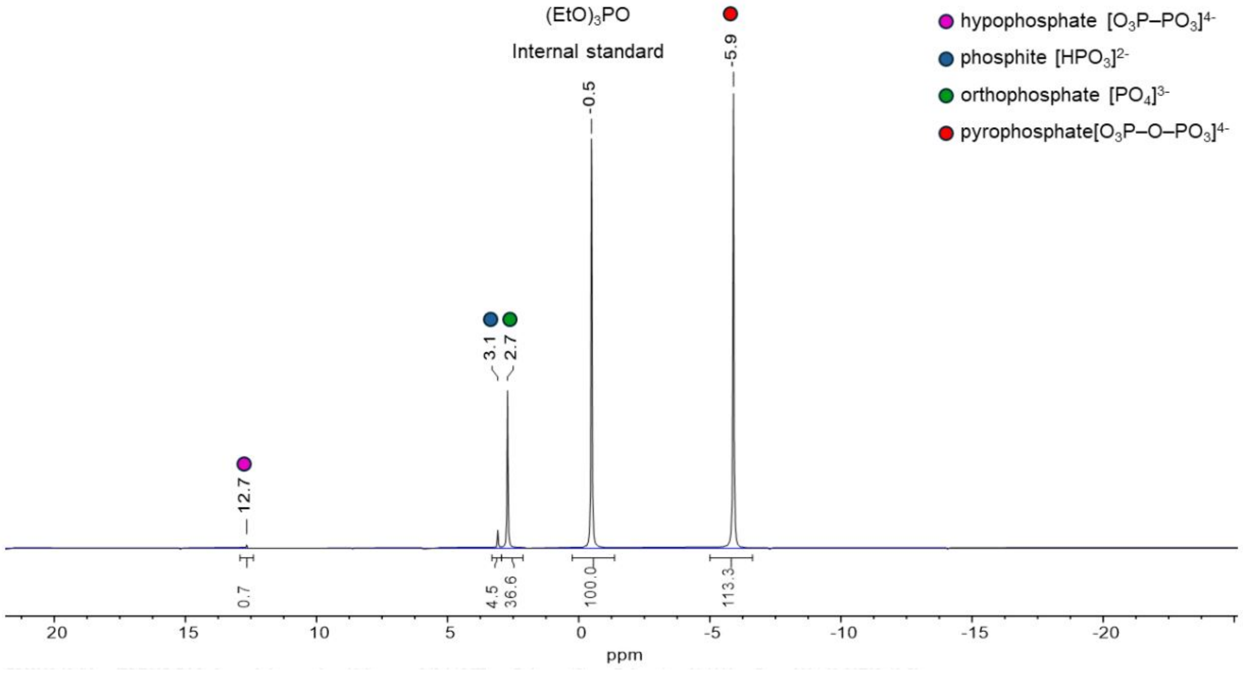

Figure S.6: ^{31}P NMR spectrum (162 MHz, H_2O/D_2O, $d1$ = 20 s, 298 K) of the reaction mixture of $K_4P_2O_7$ (3.63 mmol) with K/KI (pre-dispersed, 10% w/w, 7.27 mmol). Aliquot taken: 385 mg. $OP(OEt)_3$ (80 mg) was added as internal standard.

S.4.2.2 Quantitative ^{31}P NMR spectra

The general procedure for determining the yield is given in S.3.2. Only the spectrum window relevant for quantification is shown. Note that the compound $[O_3P–O–PH_2]^{2-}$ exhibits another signal at −178 ppm, which was not used for the quantification of the compound due to its significant deviation from the main quantification region. An exemplary full spectrum is shown in Figure S.7.

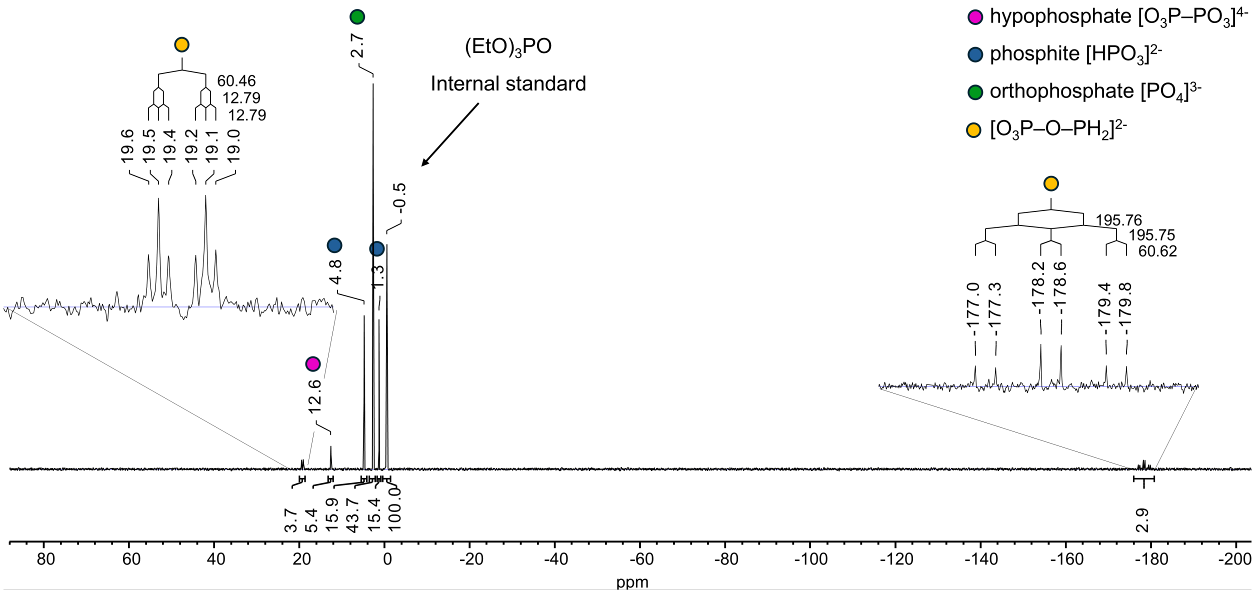

Figure S.7: Full ^{31}P NMR spectrum (162 MHz, H_2O/D_2O, $d1 = 40$ s, 298 K) of the reaction mixture of $Na_3P_3O_9$ (1.67 mmol) with K (10.0 mmol) and KI (3.52 g, K/KI ratio 10% w/w) after 12 h. Aliquot taken: 385 mg. $OP(OEt)_3$ (80 mg) was added as internal standard. Signals corresponding to $[O_3P–O–PH_2]^{2-}$ are shown zoomed in. See Figure S.10 for the corresponding standard depiction used for quantification.

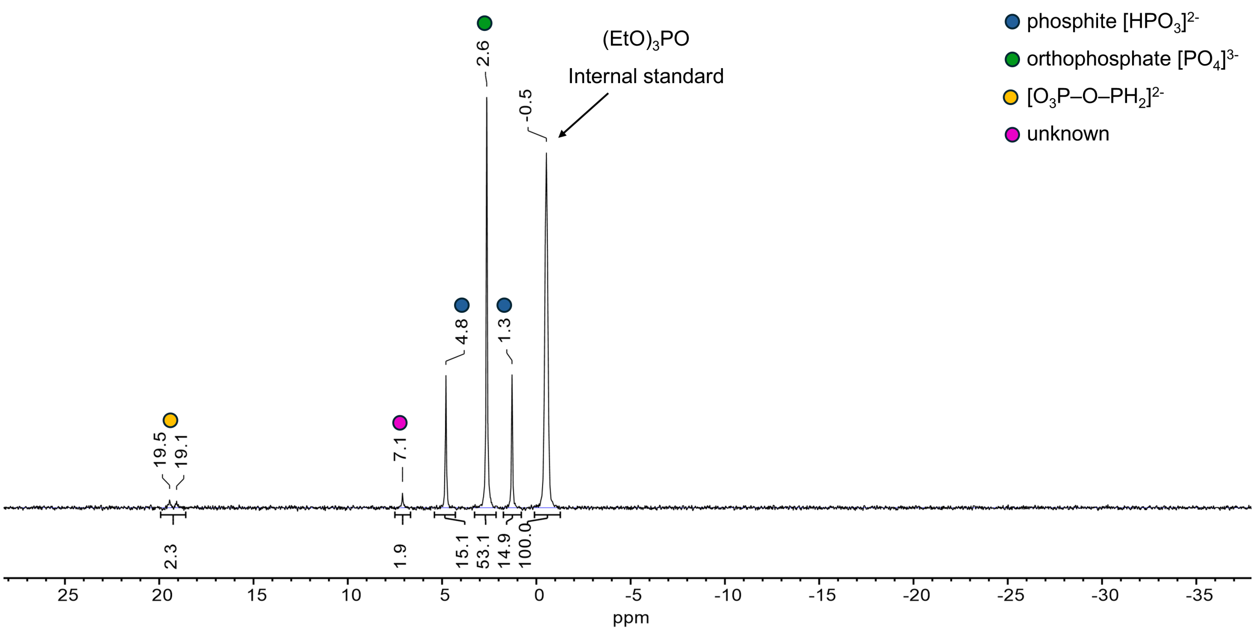

Figure S.8: ^{31}P NMR spectrum (162 MHz, H_2O/D_2O, $d1$ = 40 s, 298 K) of the reaction mixture of $Na_3P_3O_9$ (1.67 mmol) with K (10.0 mmol) and no dispersant after 12 h. Aliquot taken: 79 mg. $OP(OEt)_3$ (80 mg) was added as internal standard.

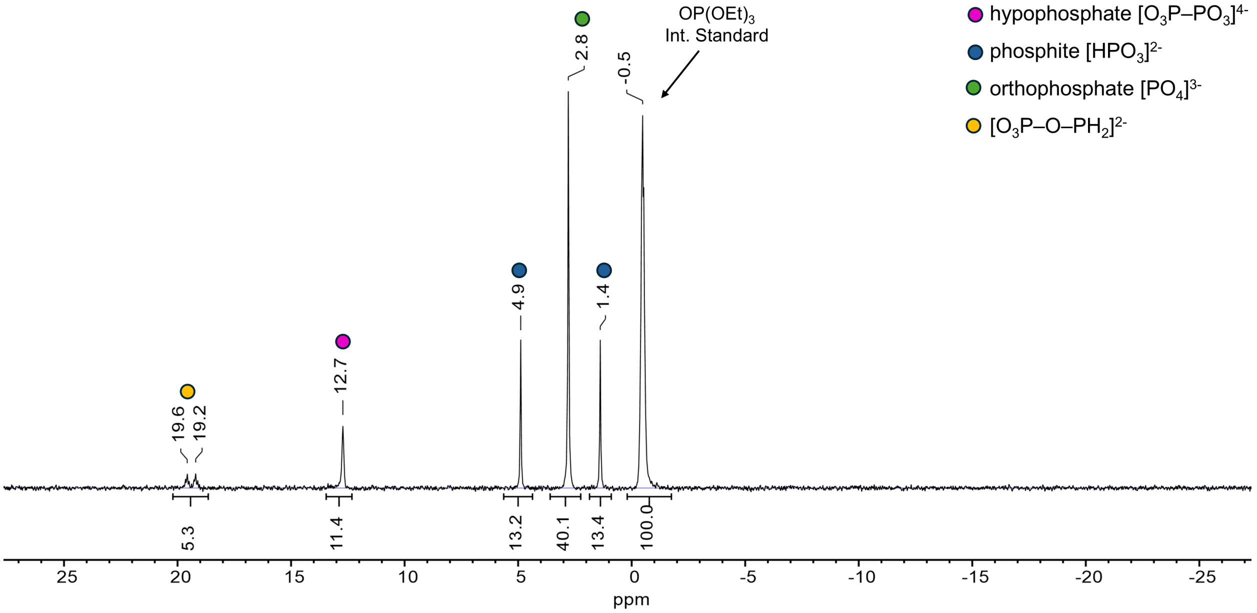

Figure S.9: ^{31}P NMR spectrum (162 MHz, H_2O/D_2O, $d1$ = 40 s, 298 K) of the reaction mixture of $Na_3P_3O_9$ (1.67 mmol) with K (10.0 mmol) and KI (7.43 g, K/KI ratio 5% w/w) after 12 h. Aliquot taken: 732 mg. $OP(OEt)_3$ (80 mg) was added as internal standard.

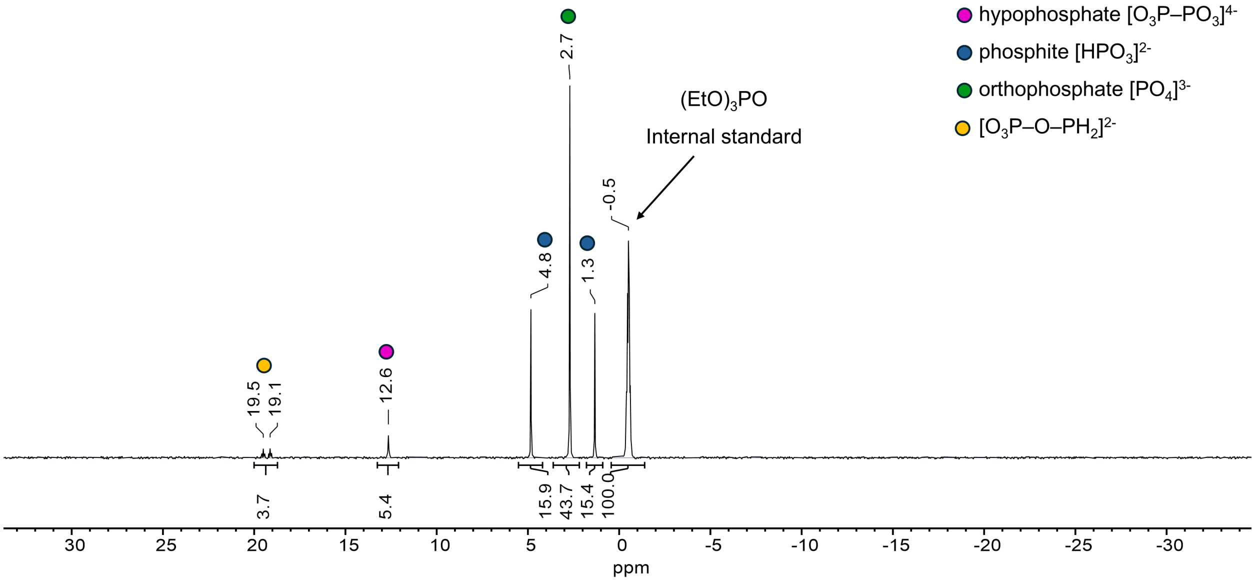

Figure S.10: ${}^{31}P$ NMR spectrum (162 MHz, H_2O/D_2O, $d1$ = 40 s, 298 K) of the reaction mixture of $Na_3P_3O_9$ (1.67 mmol) with K (10.0 mmol) and KI (3.52 g, K/KI ratio 10% w/w) after 12 h. Aliquot taken: 385 mg. $OP(OEt)_3$ (80 mg) was added as internal standard.

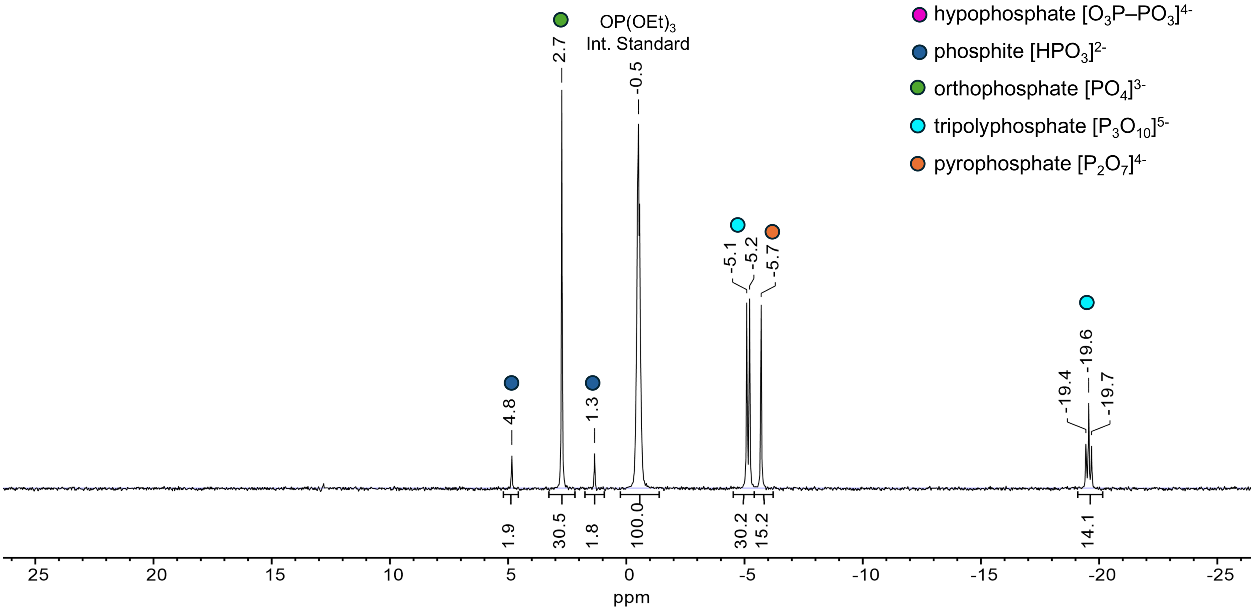

Figure S.11: ${}^{31}P$ NMR spectrum (162 MHz, H_2O/D_2O, $d1$ = 40 s, 298 K) of the reaction mixture of $Na_5P_3O_{10}$ (1.67 mmol) with K (10.0 mmol) and KI (3.52 g, K/KI ratio 10% w/w) after 12 h. Aliquot taken: 397 mg. $OP(OEt)_3$ (80 mg) was added as internal standard.

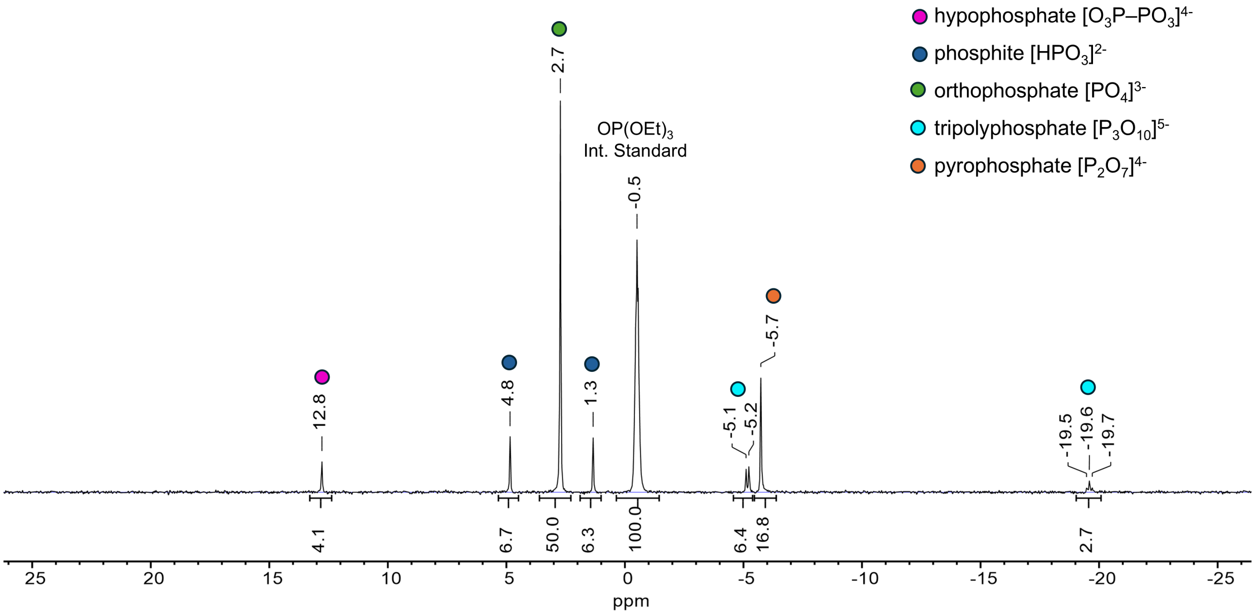

Figure S.12: ^{31}P NMR spectrum (162 MHz, H_2O/D_2O, $d1$ = 40 s, 298 K) of the reaction mixture of $Na_5P_3O_{10}$ (1.67 mmol) with K (10.0 mmol) and KI (3.52 g, K/KI ratio 10% w/w) after 24 h. Aliquot taken: 397 mg. $OP(OEt)_3$ (80 mg) was added as internal standard. Note: An aliquot of the reaction mixture was already taken after 12 h (see Figure S.11).

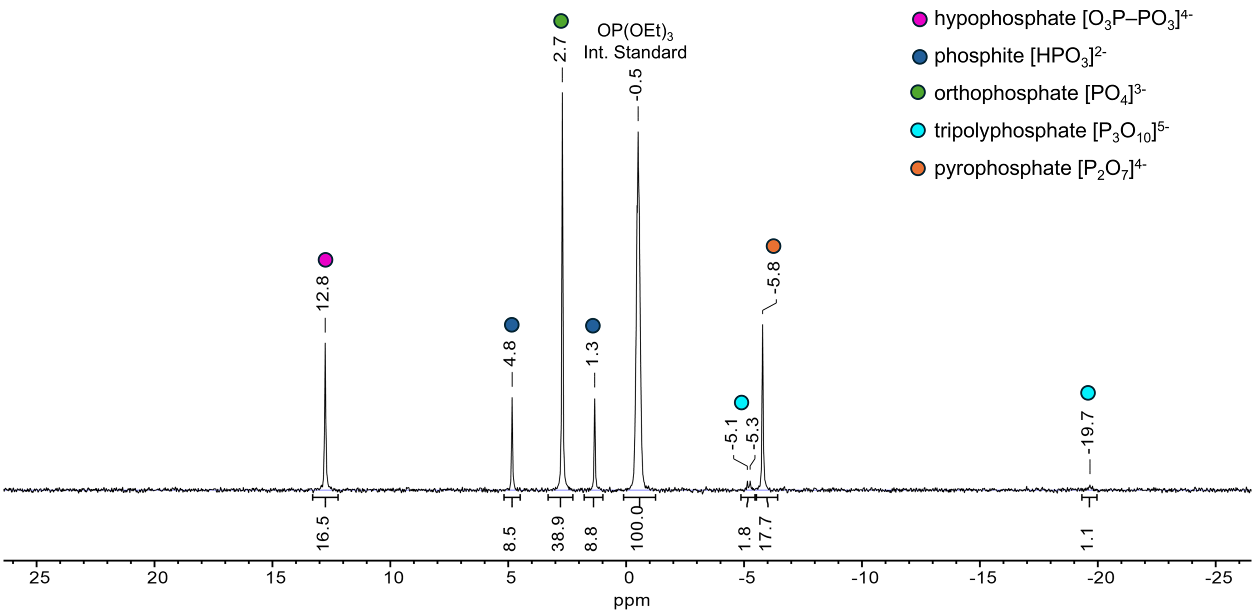

Figure S.13: ^{31}P NMR spectrum (162 MHz, H_2O/D_2O, $d1$ = 40 s, 298 K) of the reaction mixture of $Na_5P_3O_{10}$ (1.67 mmol) with K (10.0 mmol) and KI (3.52 g, K/KI ratio 10% w/w) after 36 h. Aliquot taken: 397 mg. $OP(OEt)_3$ (80 mg) was added as internal standard. Note: Aliquots of the reaction mixture were already taken after 12 h and 24 h (see Figures S.11 and S.12).

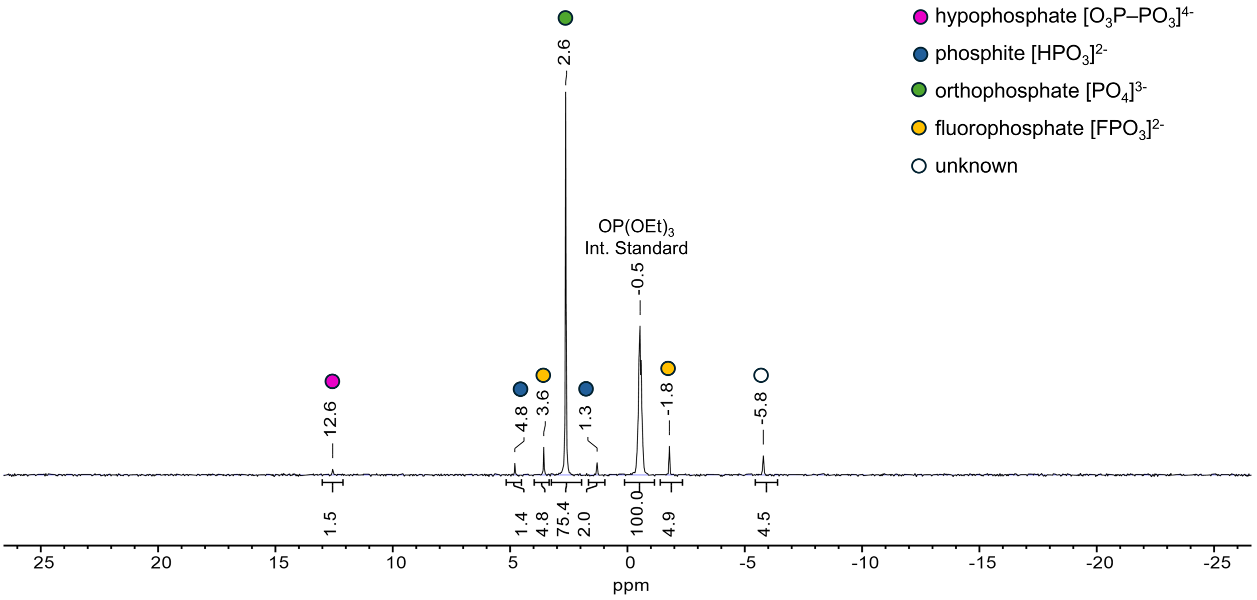

Figure S.14: ^{31}P NMR spectrum (162 MHz, H_2O/D_2O, $d1$ = 40 s, 298 K) of the reaction mixture of Na_2PO_3F (5.00 mmol) with K (10.0 mmol) without dispersant after 12 h at 450 rpm. Aliquot taken: 97.6 mg. $OP(OEt)_3$ (80 mg) was added as internal standard.

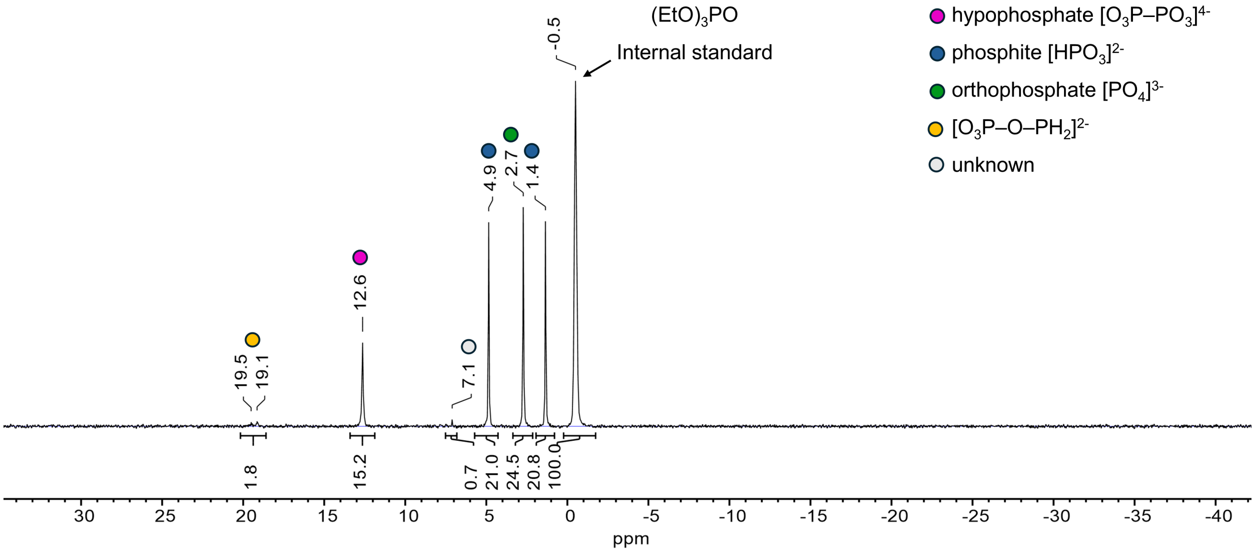

Figure S.15: ^{31}P NMR spectrum (162 MHz, H_2O/D_2O, $d1$ = 40 s, 298 K) of the reaction mixture of Na_2PO_3F (2.07 mmol) with K (4.14 mmol) and KI (1.46 g, K/KI ratio 10% w/w) after 12 h at 450 rpm. Aliquot taken: 385 mg. $OP(OEt)_3$ (80 mg) was added as internal standard.

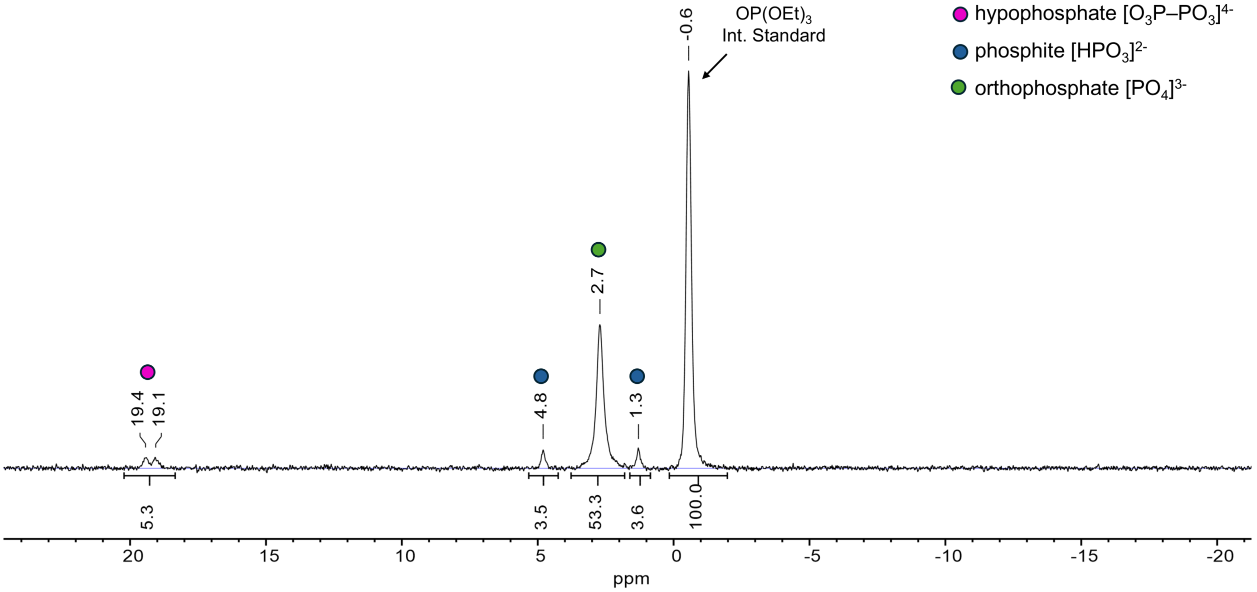

Figure S.16: ^{31}P NMR spectrum (162 MHz, H_2O/D_2O, $d1$ = 40 s, 298 K) of the reaction mixture of $(KPO_3)_n$ (5.00 mmol) with K (10.0 mmol) and no dispersant after 24 h at 450 rpm. Aliquot taken: 86.2 mg. $OP(OEt)_3$ (80 mg) was added as internal standard.

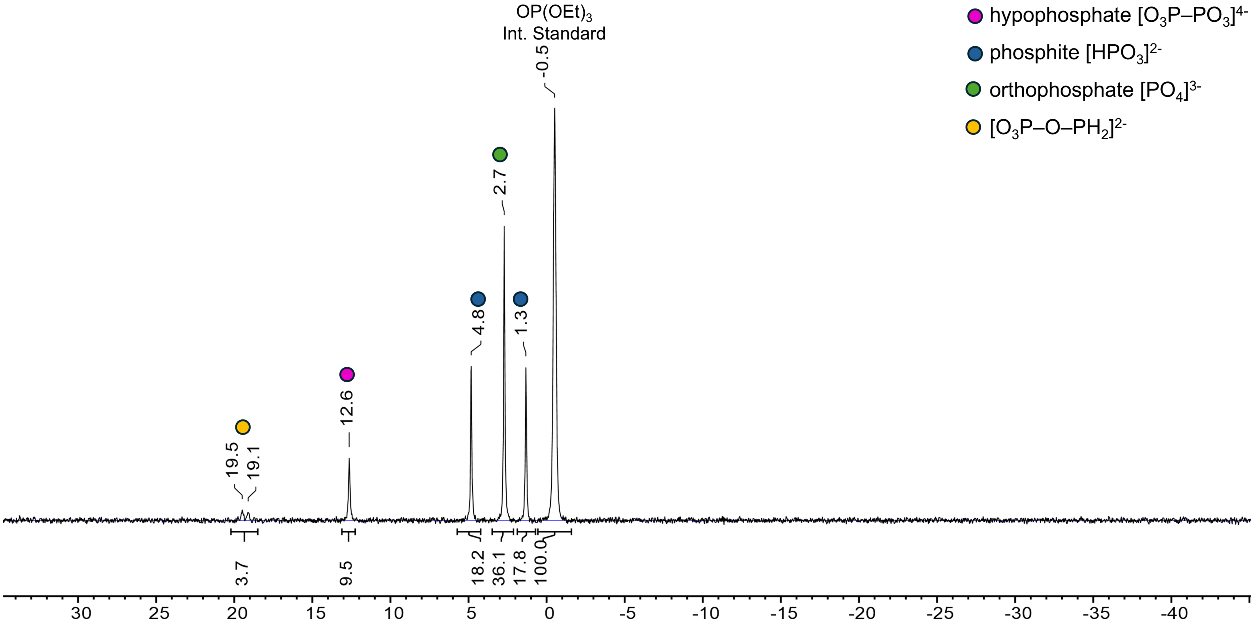

Figure S.17: ^{31}P NMR spectrum (162 MHz, H_2O/D_2O, $d1$ = 40 s, 298 K) of the reaction mixture of $(KPO_3)_n$ (5.00 mmol) with K (10.0 mmol) and KI (3.52 g, K/KI ratio 10% w/w) after 24 h at 450 rpm. Aliquot taken: 395 mg. $OP(OEt)_3$ (80 mg) was added as internal standard.

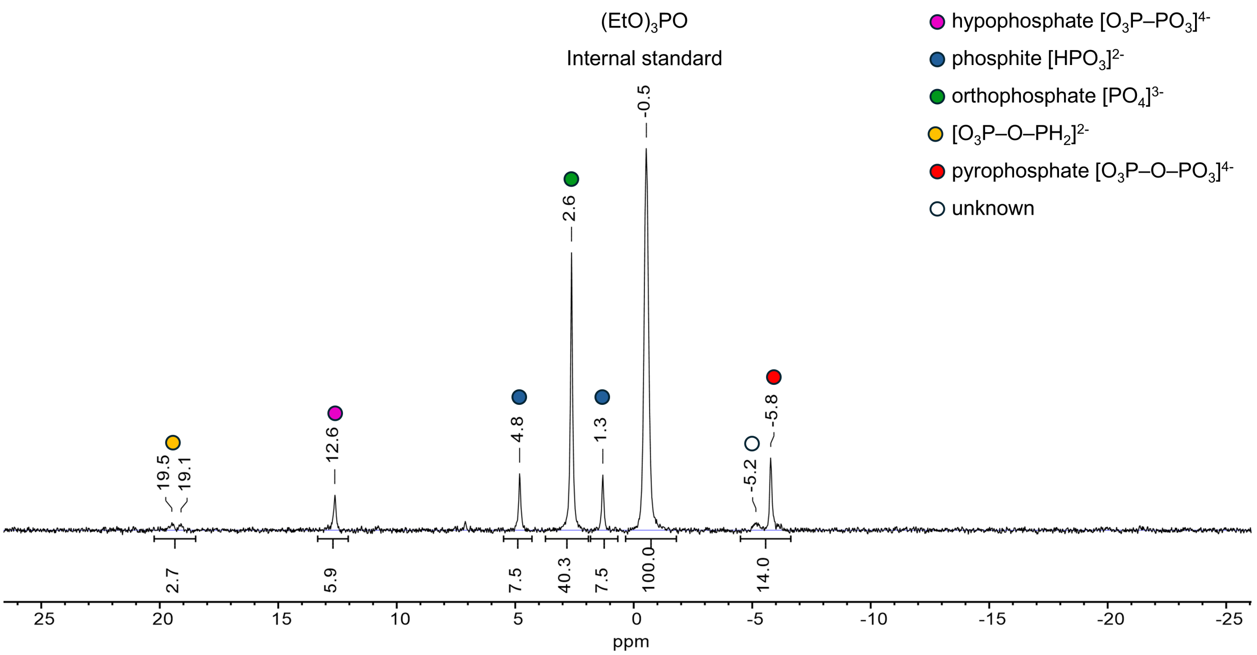

Figure S.18: ^{31}P NMR spectrum (162 MHz, H_2O/D_2O, $d1$ = 40 s, 298 K) of the reaction mixture of $Na_3P_3O_9$ (1.67 mmol) with Na (10.0 mmol) and no dispersant after 12 h. Aliquot taken: 65.1 mg. $OP(OEt)_3$ (80 mg) was added as internal standard.

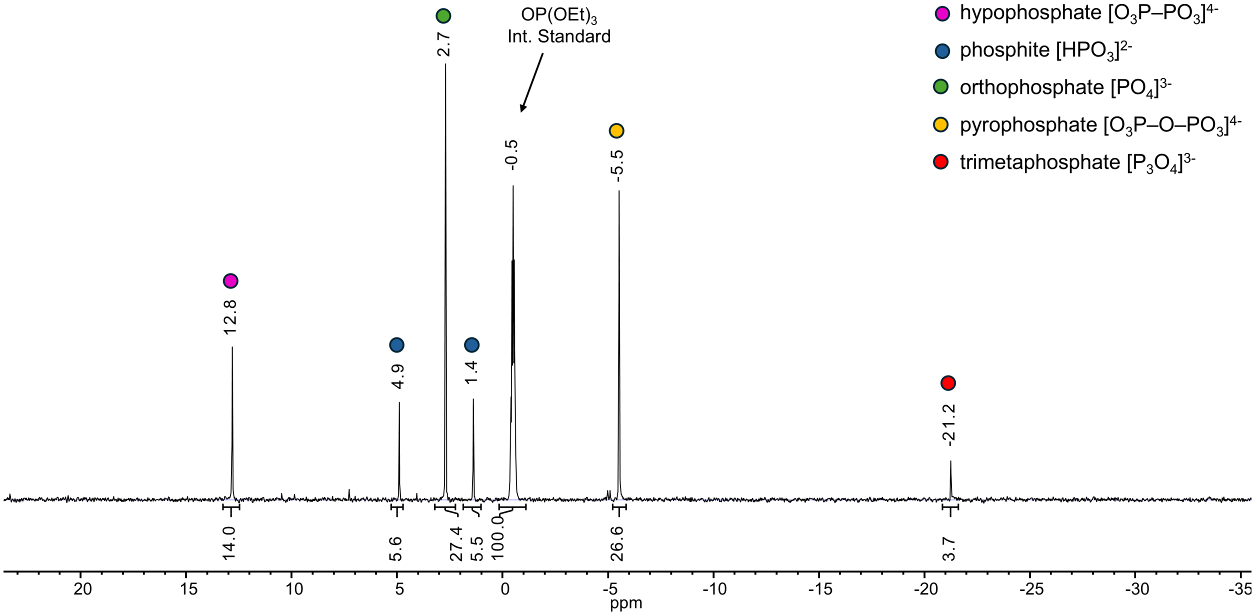

Figure S.19: ^{31}P NMR spectrum (162 MHz, H_2O/D_2O, $d1$ = 40 s, 298 K) of the reaction mixture of $Na_3P_3O_9$ (1.67 mmol) with Na (10.0 mmol) and NaCl (3.52 g, Na/NaCl ratio 10% w/w) after 12 h. Aliquot taken: 247 mg. $OP(OEt)_3$ (80 mg) was added as internal standard.

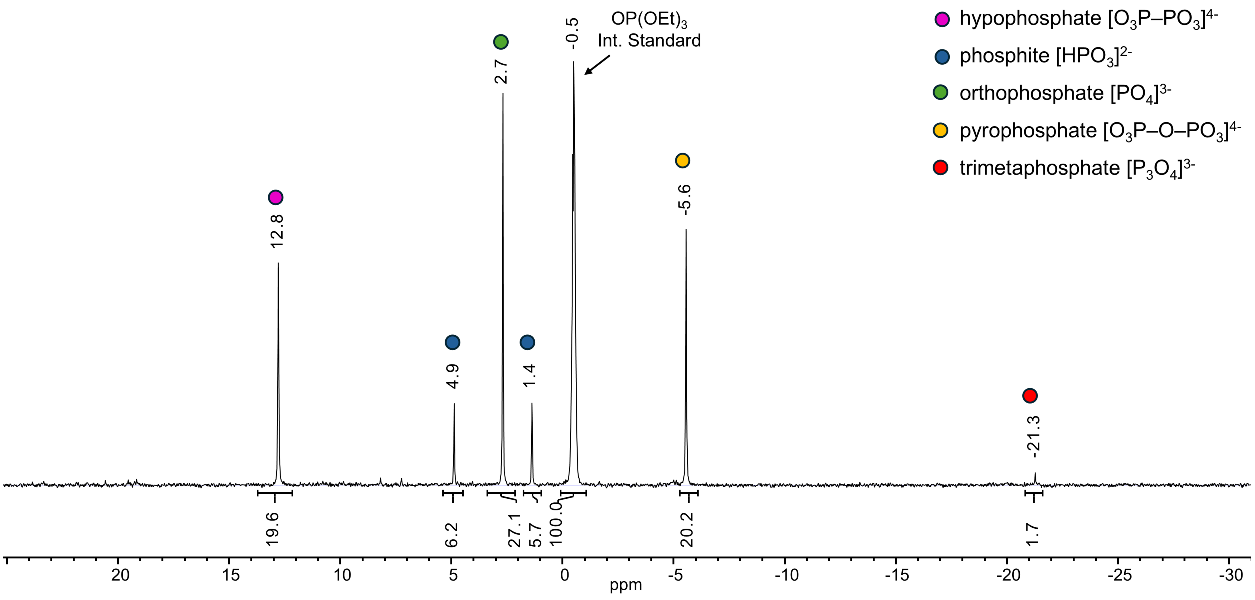

Figure S.20: ^{31}P NMR spectrum (162 MHz, H_2O/D_2O, $d1 = 40$ s, 298 K) of the reaction mixture of $Na_3P_3O_9$ (1.67 mmol) with Na (10.0 mmol) and NaCl (3.52 g, Na/NaCl ratio 10% w/w) after 24 h. Aliquot taken: 247 mg. $OP(OEt)_3$ (80 mg) was added as internal standard.

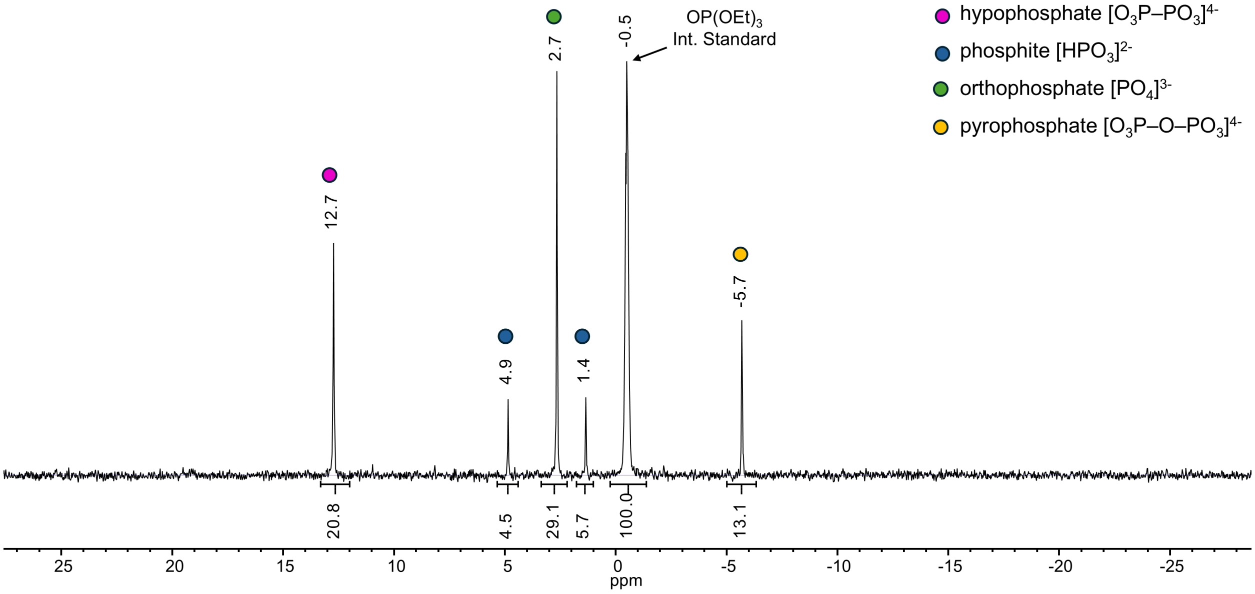

Figure S.21: ^{31}P NMR spectrum (162 MHz, H_2O/D_2O, $d1 = 40$ s, 298 K) of the reaction mixture of $Na_3P_3O_9$ (1.67 mmol) with Na (10.0 mmol) and NaCl (3.52 g, Na/NaCl ratio 10% w/w) after 36 h. Aliquot taken: 247 mg. $OP(OEt)_3$ (80 mg) was added as internal standard. Note: An aliquot of the reaction was already taken after 24 h (see Figure S.20).

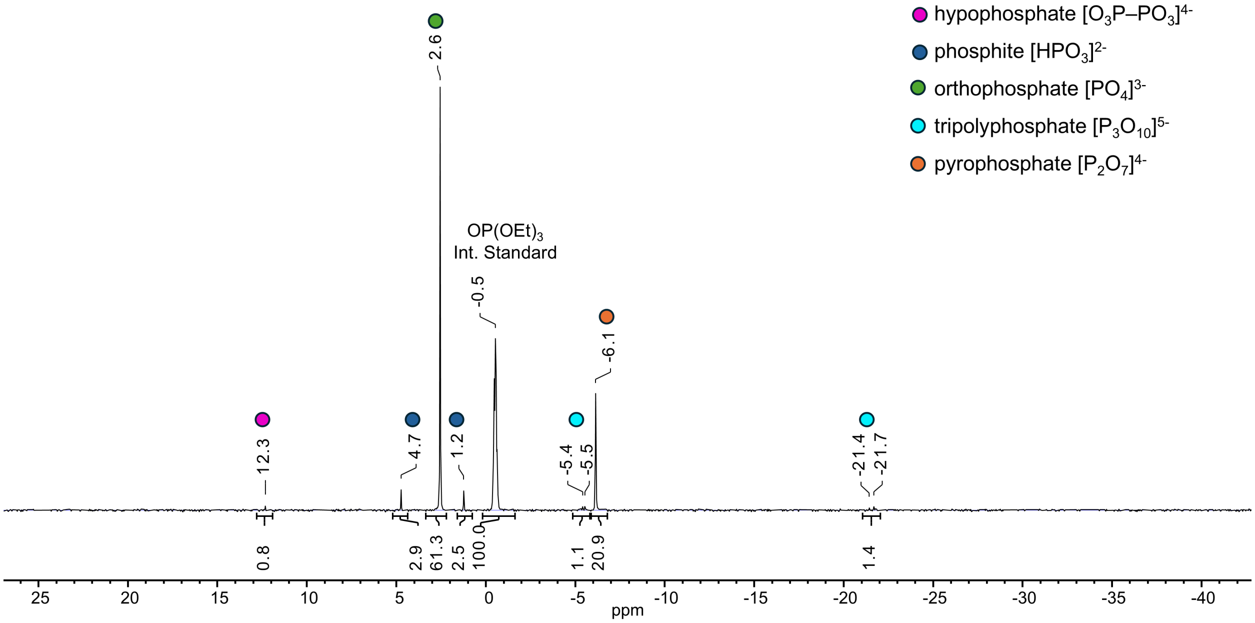

Figure S.22: ${}^{31}P$ NMR spectrum (162 MHz, H_2O/D_2O, $d1$ = 40 s, 298 K) of the reaction mixture of $Na_3P_3O_9$ (1.67 mmol) with Cs (10.0 mmol) and no dispersant after 12 h. Aliquot taken: 162 mg. $OP(OEt)_3$ (80 mg) was added as internal standard. Note: An aliquot of the reaction mixture was already taken after 24 h (see Figure S.22).

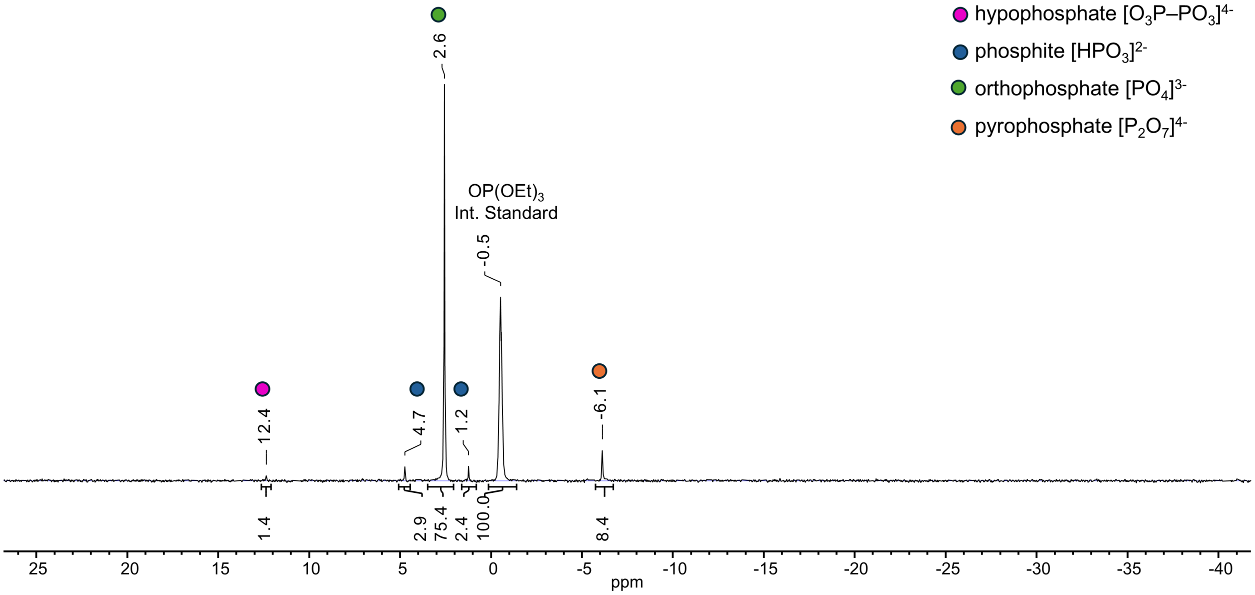

Figure S.23: ${}^{31}P$ NMR spectrum (162 MHz, H_2O/D_2O, $d1$ = 40 s, 298 K) of the reaction mixture of $Na_3P_3O_9$ (1.67 mmol) with Cs (10.0 mmol) and no dispersant after 24 h. Aliquot taken: 162 mg. $OP(OEt)_3$ (80 mg) was added as internal standard. Note: An aliquot of the reaction mixture was already taken after 12 h (see Figure S.22).

S.4.3 Orthogonal Synthesis of Ortho-phosphite

S.4.3.1 From Na_2HPO_3 and [(Trimethylsilyl)methyl]sodium ($NaCH_2SiMe_3$)

$NaCH_2SiMe_3$ was synthesized according to the literature procedure.[5] Inside the glovebox, anhydrous Na_2HPO_3 (450 mg, 3.48 mmol, 1.00 eq.), $NaCH_2SiMe_3$ (391 mg, 3.48 mmol, 1.00 eq., and thirty stainless steel balls (∅ 10 mm) were added into a 125 mL ball-milling jar. The jar was sealed properly and brought out. The mixture was ball milled at 200 rpm for 5 min, then 450 rpm for 12 h (with cooling breaks of 30 min every 1 h and direction change every cycle, total process time 24 h.). When the desired grinding time was reached, the jar was brought into the glovebox and opened. an aliquot (100 mg) was taken out and hydrolyzed with D_2O and analyzed with ^{31}P NMR spectroscopy (see Figure S.26). Then the reaction mixture was bill milled again for 36 h and 48 h. An aliquot of each fraction was analyzed by ^{31}P NMR spectroscopy (see Figure S.27 and S.28 respectively). The reaction mixture was isolated in a vial.

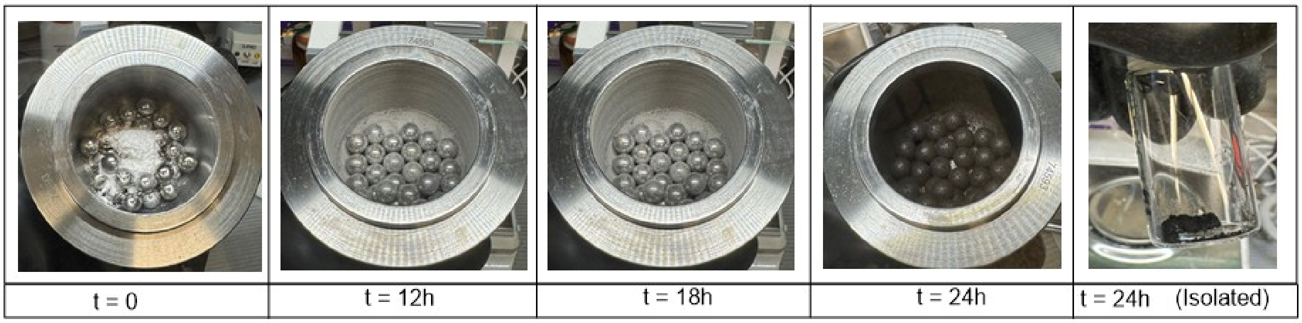

Figure S.24: Ball-mill jar before and after reaction, showing crude product at different reaction times.

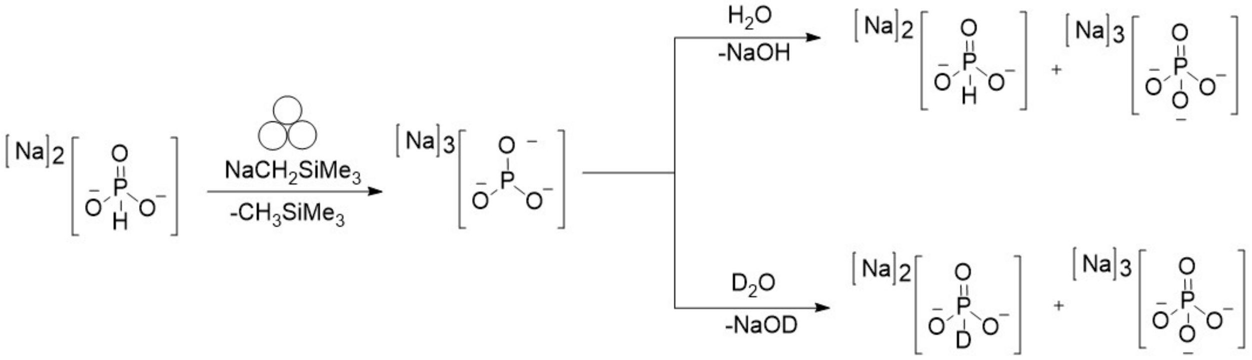

Figure S.25: Synthesis of ortho-phosphite from sodium phosphite using $NaCH_2SiMe_3$ and subsequent hydrolysis of the ortho-phosphite with H_2O and D_2O.

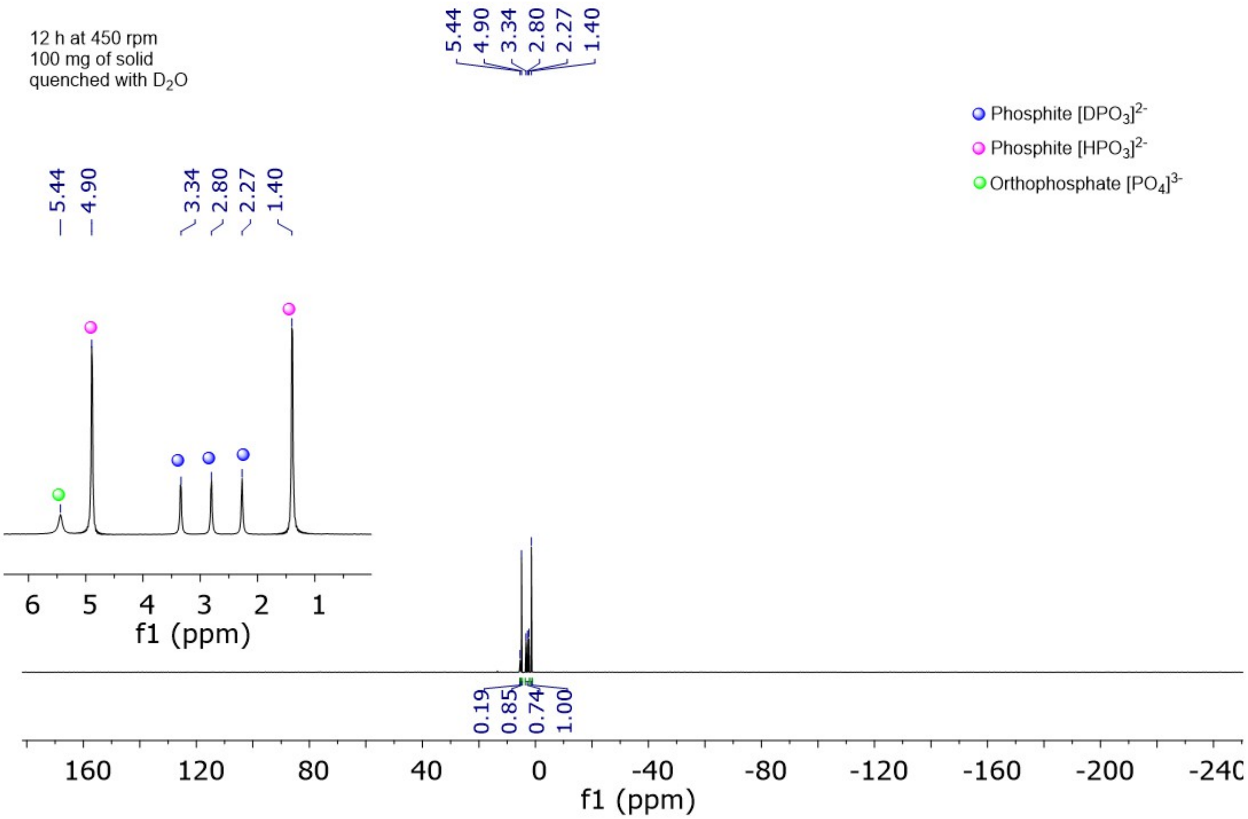

Figure S.26: ^{31}P NMR spectrum (162 MHz, 298 K, $d1$ = 2 s) of the hydrolysis reaction of the crude mixture with D_2O after 12 h.

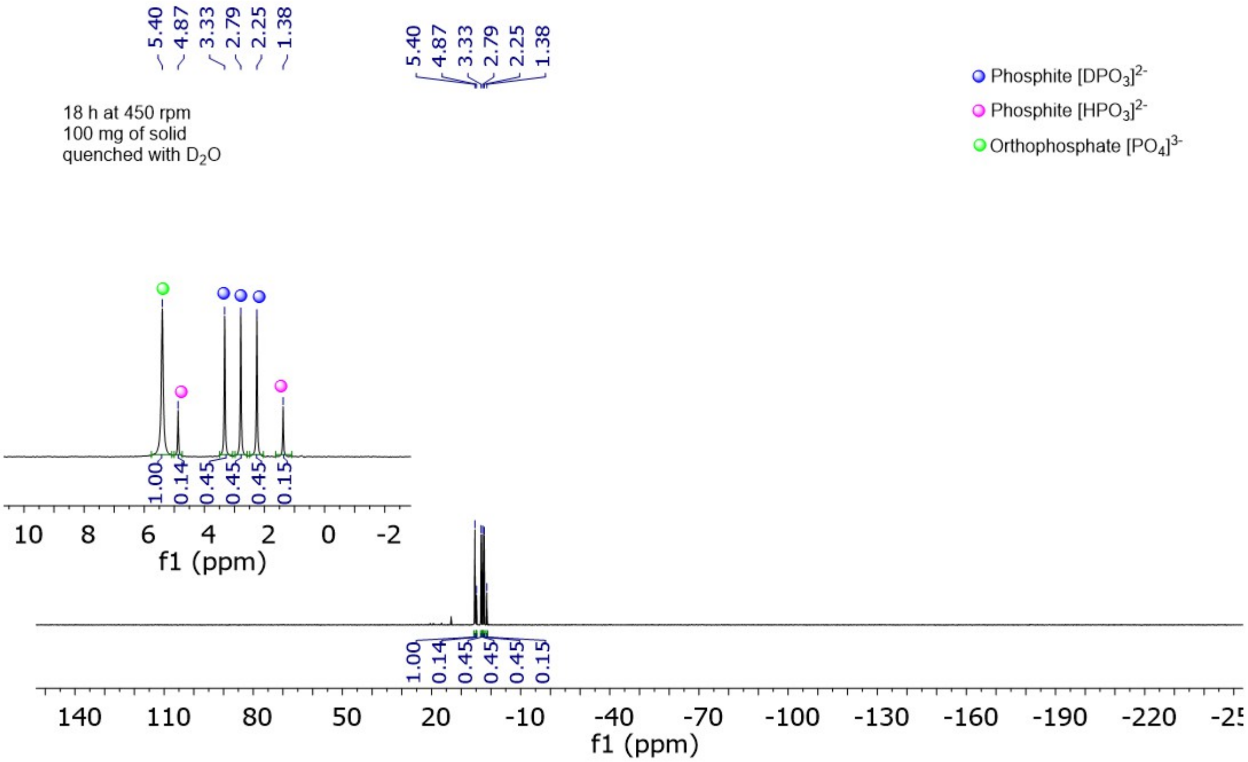

Figure S.27: ^{31}P NMR spectrum (162 MHz, 298 K, $d1 = 2$ s) of the hydrolysis reaction of the crude mixture with D_2O after 18 h.

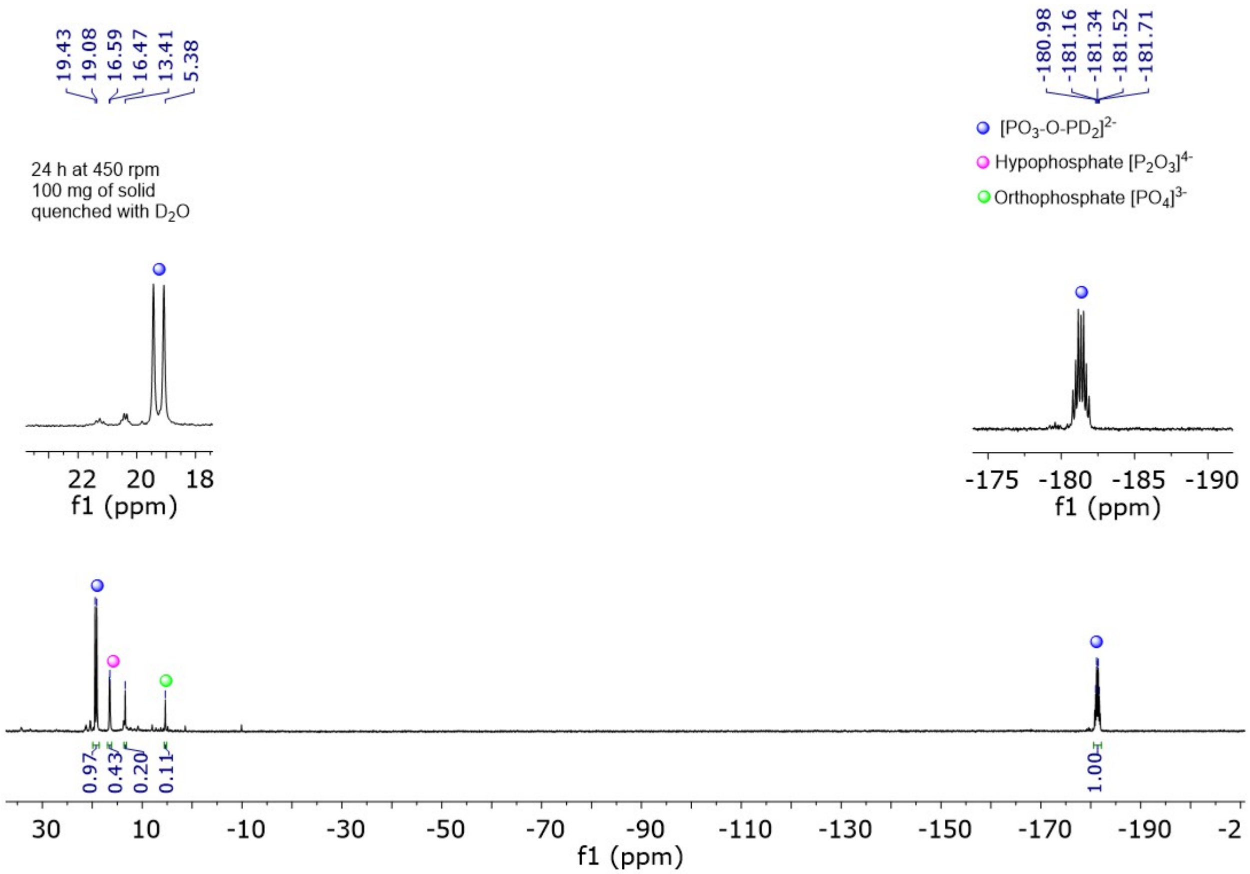

Figure S.28: ^{31}P NMR spectrum (162 MHz, 298 K, $d1$ = 2 s) of the hydrolysis reaction of the crude mixture with D_2O after 24 h .

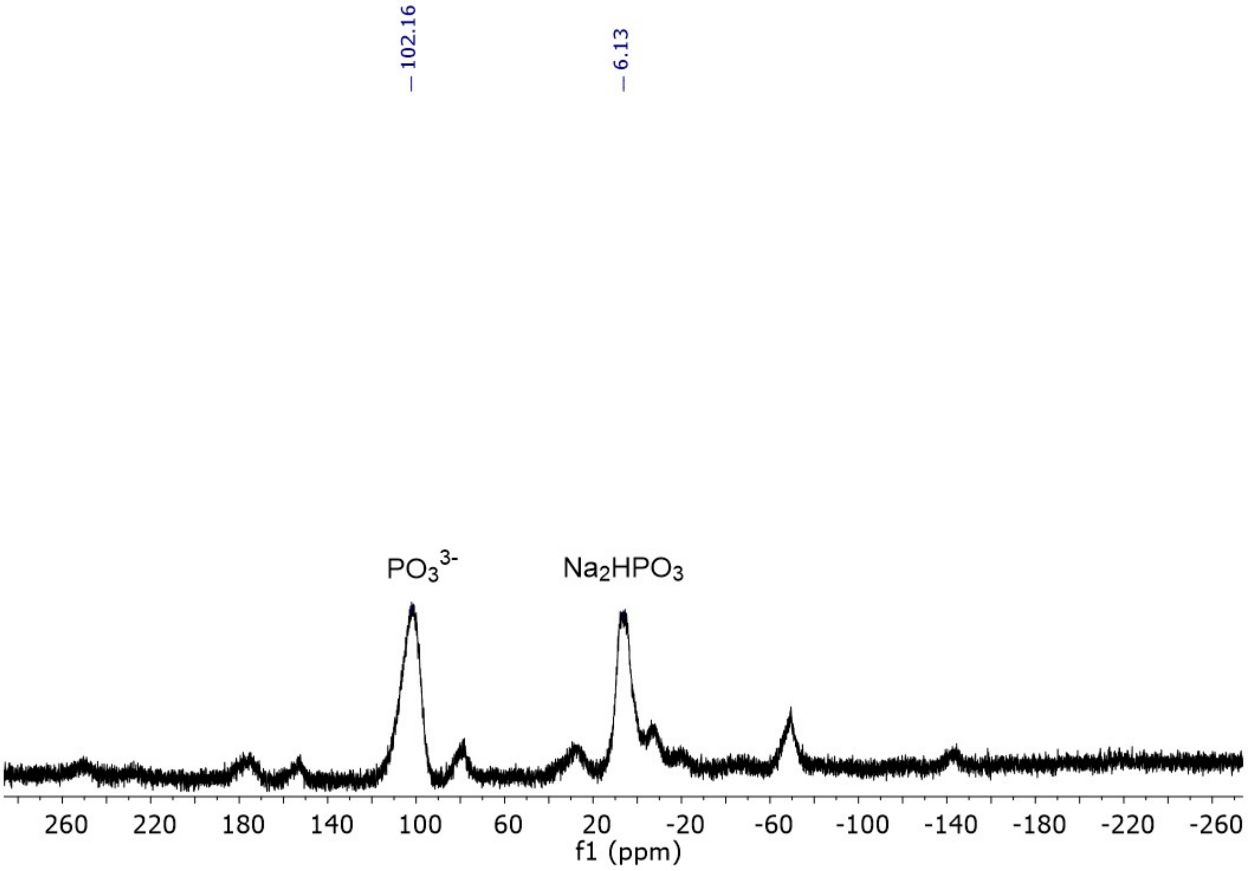

Figure S.29: ^{31}P solid-state NMR spectrum (15 kHz MAS, 202 MHz, 298 K, $d1 = 2$ s) of the reaction mixture of Na_2HPO_3 (3.48 mmol) with $NaCH_2SiMe_3$ (3.48 mmol) after 18 h at 450 rpm.

S.4.3.2 From Na_2HPO_3 and Benzyl Potassium (BnK)

Benzyl potassium was synthesized according to the literature procedure.[6] Inside the glovebox, anhydrous Na_2HPO_3 (438 mg, 3.48 mmol, 1.0 equiv) and benzyl potassium (453 mg, 3.48 mmol, 1.0 equiv) were charged into a 125 mL stainless steel milling jar together with thirty stainless steel balls (∅ 10 mm). The jar was tightly sealed and removed from the glovebox for milling. The mixture was initially milled at 200 rpm for 5 mins, followed by 450 rpm for 24 h, with 30 min cooling breaks after each hour of milling and reversal of rotation direction between cycles (total process duration: 48 h).

After completion of the milling, the jar was returned to the glovebox and opened. Two separate aliquots (100 mg each) of the milled material were withdrawn, hydrolyzed with H_2O and D_2O, and analyzed by solution-phase ^{31}P NMR spectroscopy (Figure S.33 and Figure S.32). An additional portion of the sample was analyzed by solid-state ^{31}P NMR spectroscopy (Figure S.34).

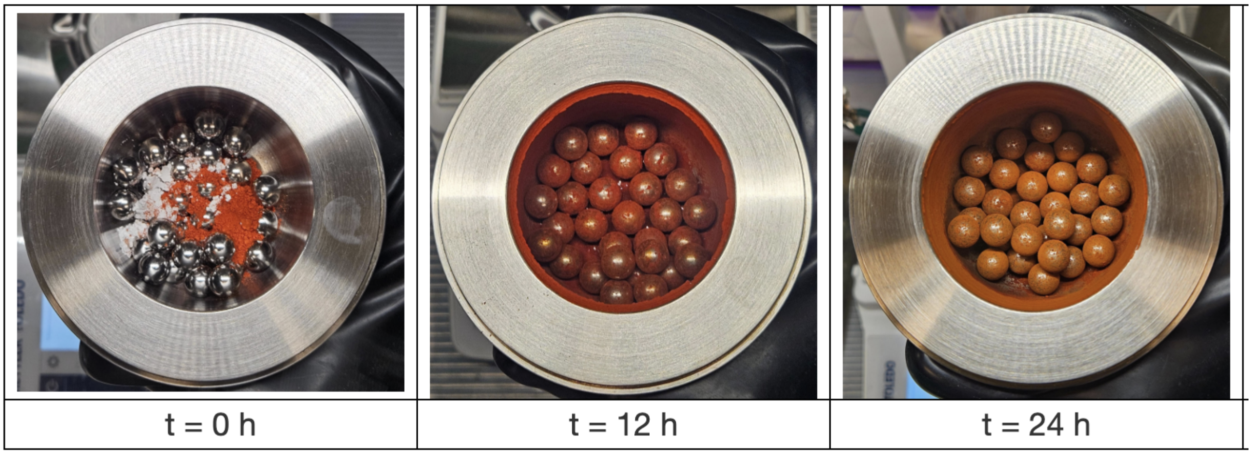

Figure S.30: Reaction progress at different timescale.

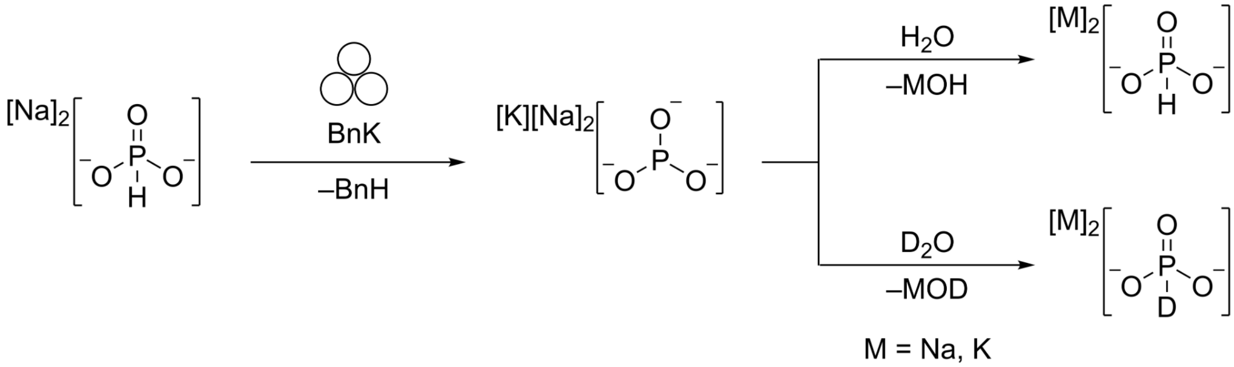

Figure S.31: Synthesis of ortho-phosphite from sodium phosphite using benzyl potassium and subesequent hydrolysis of the ortho-phosphite.

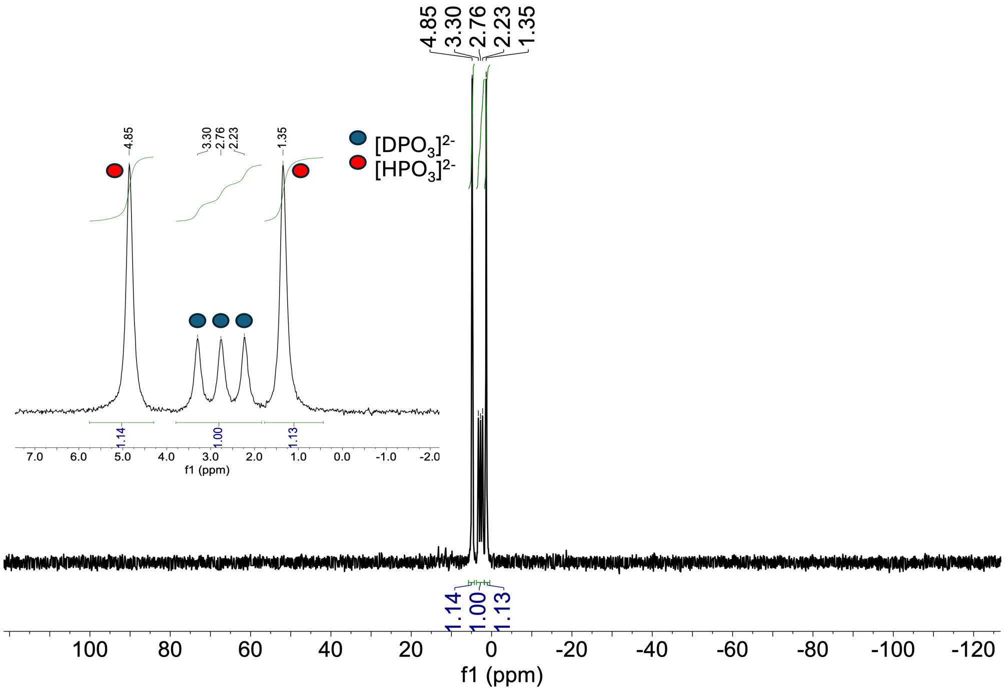

Figure S.32: ^{31}P NMR spectrum (162 MHz, 298 K, $d1$ = 40 s) of the hydrolysis reaction of the crude mixture with D_2O after 24 h.

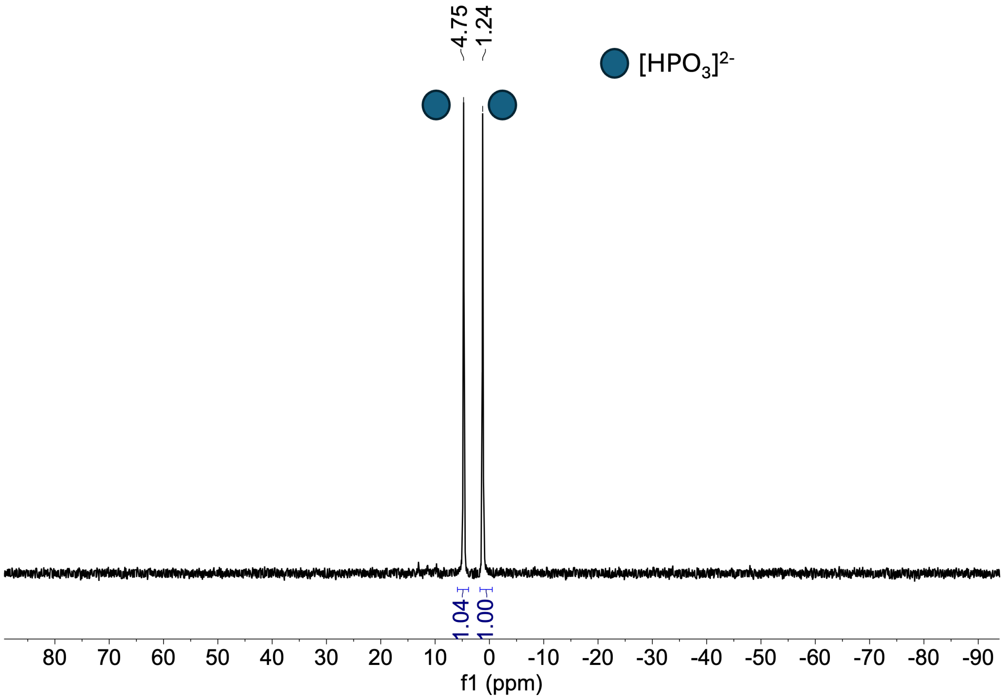

Figure S.33: ^{31}P NMR spectrum (162 MHz, 298 K, $d1$ = 40 s) of the hydrolysis reaction of the crude mixture with H_2O after 24 h.

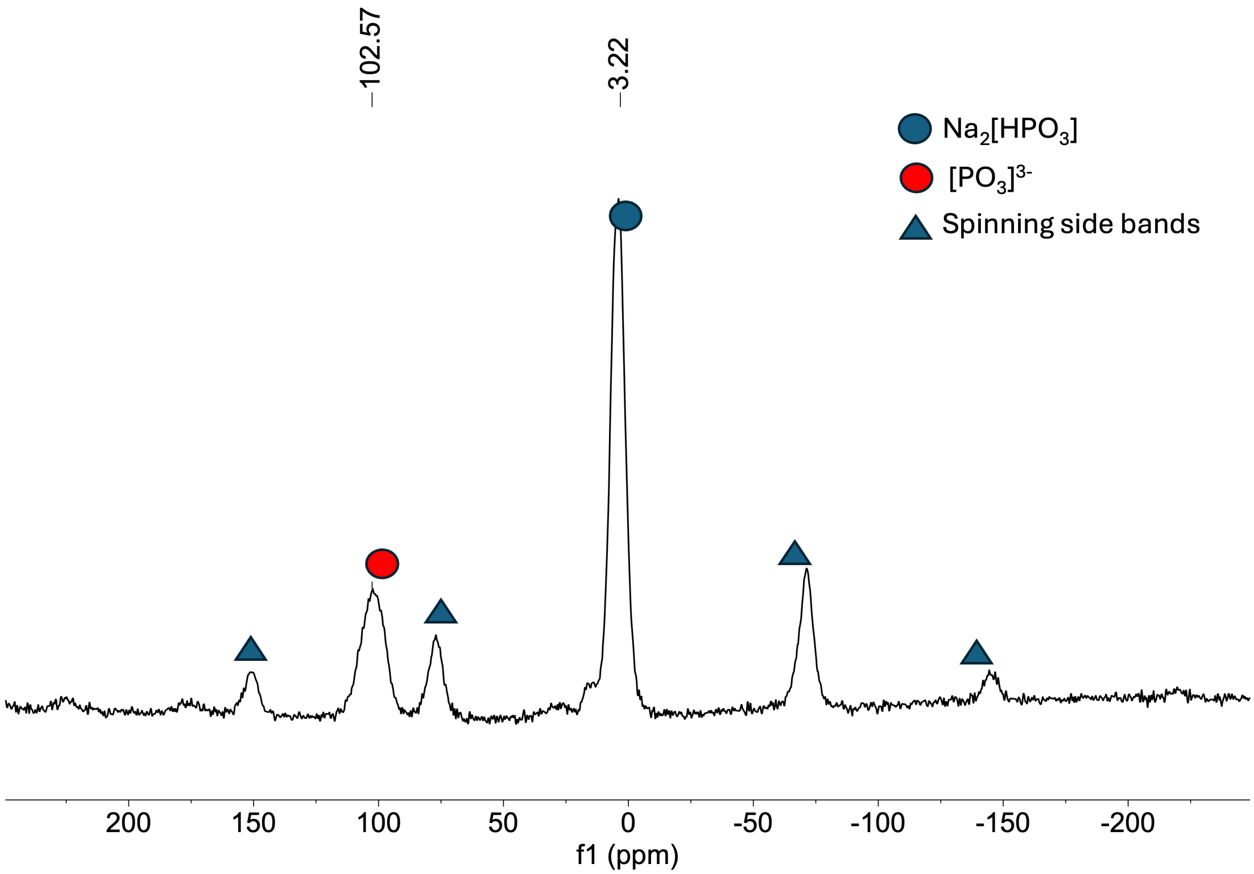

Figure S.34: ^{31}P solid-state NMR spectrum (15 kHz MAS, 202 MHz, 298 K, $d1 = 4$ s) of the reaction mixture of Na_2HPO_3 (3.48 mmol) with BnK (3.48 mmol) after 24 h at 450 rpm.

S.4.3.3 From K_2HPO_3 and Benzyl Potassium (BnK)

Anhydrous potassium phosphite (K_2HPO_3) was prepared by the neutralization of phosphorous acid (H_3PO_3) with two equivalents of potassium hydroxide (KOH) in water, followed by drying in an oven at 200 °C for two days. Inside the glovebox, potassium phosphite (K_2HPO_3, 500 mg, 3.16 mmol, 1.0 equiv) and benzyl potassium (823 mg, 6.32 mmol, 2.0 equiv) were charged into a 125 mL stainless steel milling jar together with thirty stainless steel balls (10 mm diameter). The jar was tightly sealed and removed from the glovebox for milling. The mixture was first milled at 200 rpm for 5 min, followed by 450 rpm for 24 h, with 30 min cooling breaks after each hour of milling and reversal of rotation direction between cycles (total process duration: 48 h).

After completion of the milling, the jar was returned to the glovebox and opened. An aliquot (100 mg) of the milled material was withdrawn, hydrolyzed with D_2O, and analyzed by solution-phase ^{31}P NMR spectroscopy (Figure S.37). An additional portion of the sample was analyzed by solid-state ^{31}P NMR spectroscopy (Figure S.38).

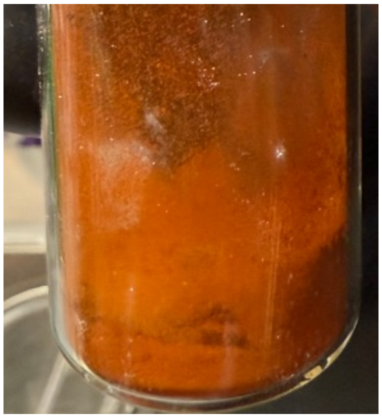

Figure S.35: Isolated crude product after 24 h.

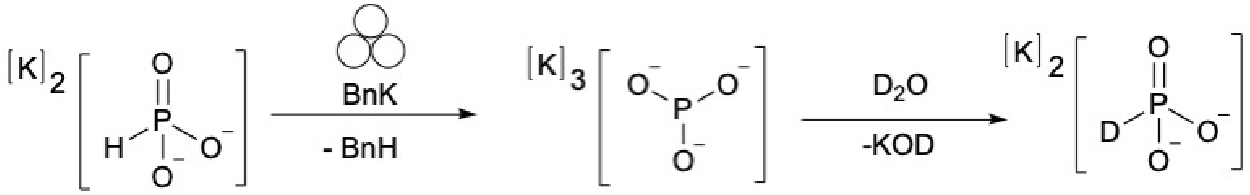

Figure S.36: Synthesis of ortho-phosphite from potassium phosphite using benzyl potassium and subsequent hydrolysis of ortho-phosphite.

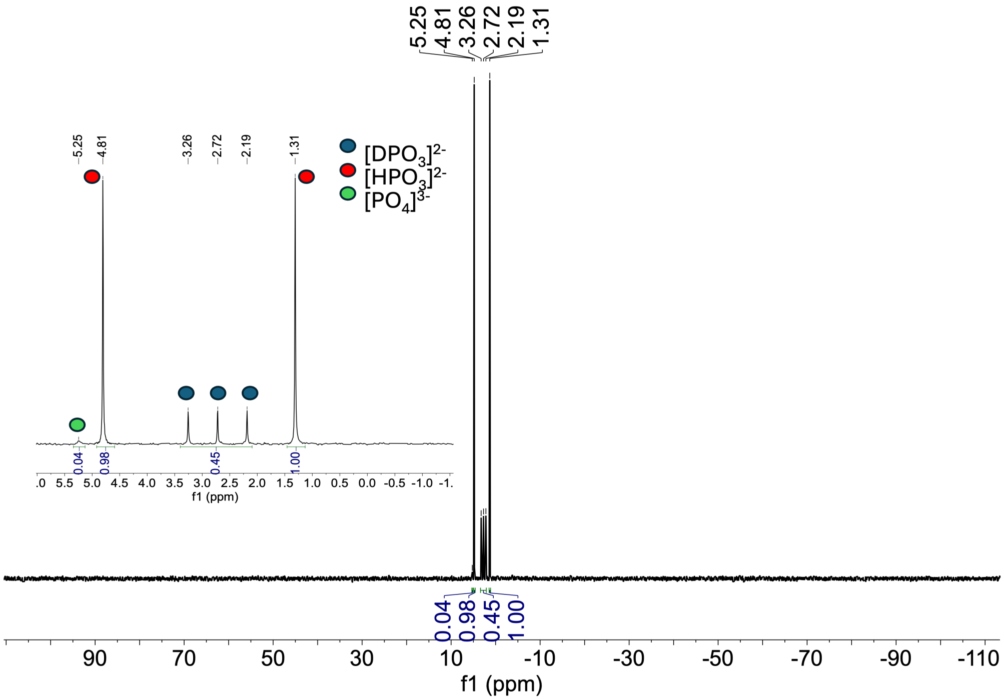

Figure S.37: ^{31}P NMR spectrum (162 MHz, 298 K, $d1 = 40$ s) of the hydrolysis reaction of the crude mixture with D_2O after 24 h.

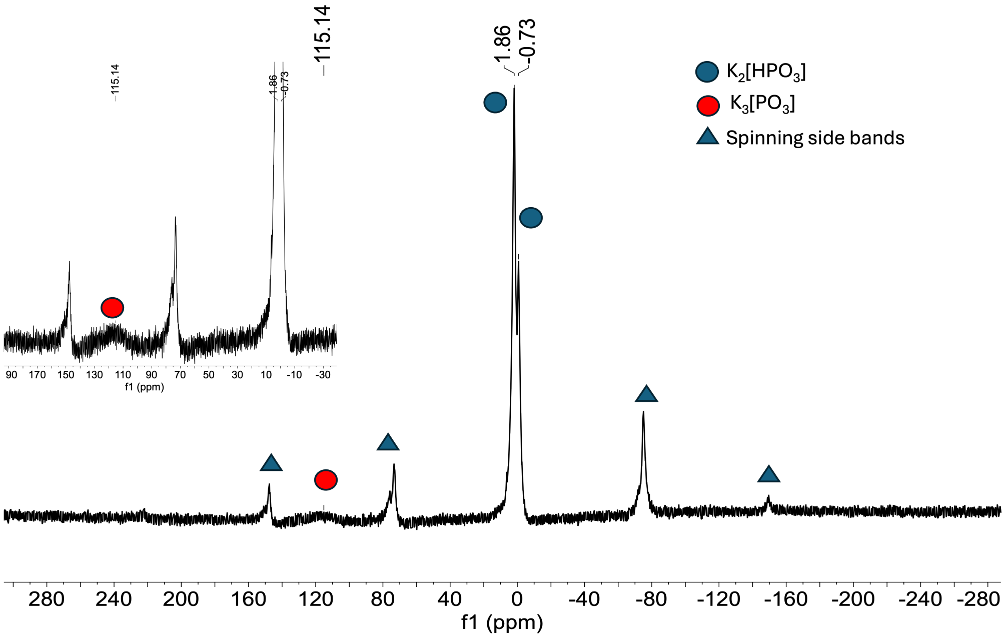

Figure S.38: ^{31}P solid-state NMR spectrum (15 kHz MAS, 202 MHz, 298 K, $d1 = 4$ s) of the reaction mixture of K_2HPO_3 (3.16 mmol) with BnK (6.32 mmol) after 24 h at 450 rpm.

S.4.4 Attempted Isotopic Exchange of Hydrogen with Deuterium in Na_2HPO_3

Na_2HPO_3 (50 mg) was dissolved in D_2O (2 mL) in a 20 mL vial and stirred at room temperature for 2 h at pH = 13. An aliquot was subsequently analyzed by ^{31}P NMR spectroscopy, which revealed no evidence of H/D exchange. The mixture was then stirred overnight, and a second aliquot was analyzed under the same conditions; but no evidence of H/D exchange was observed.

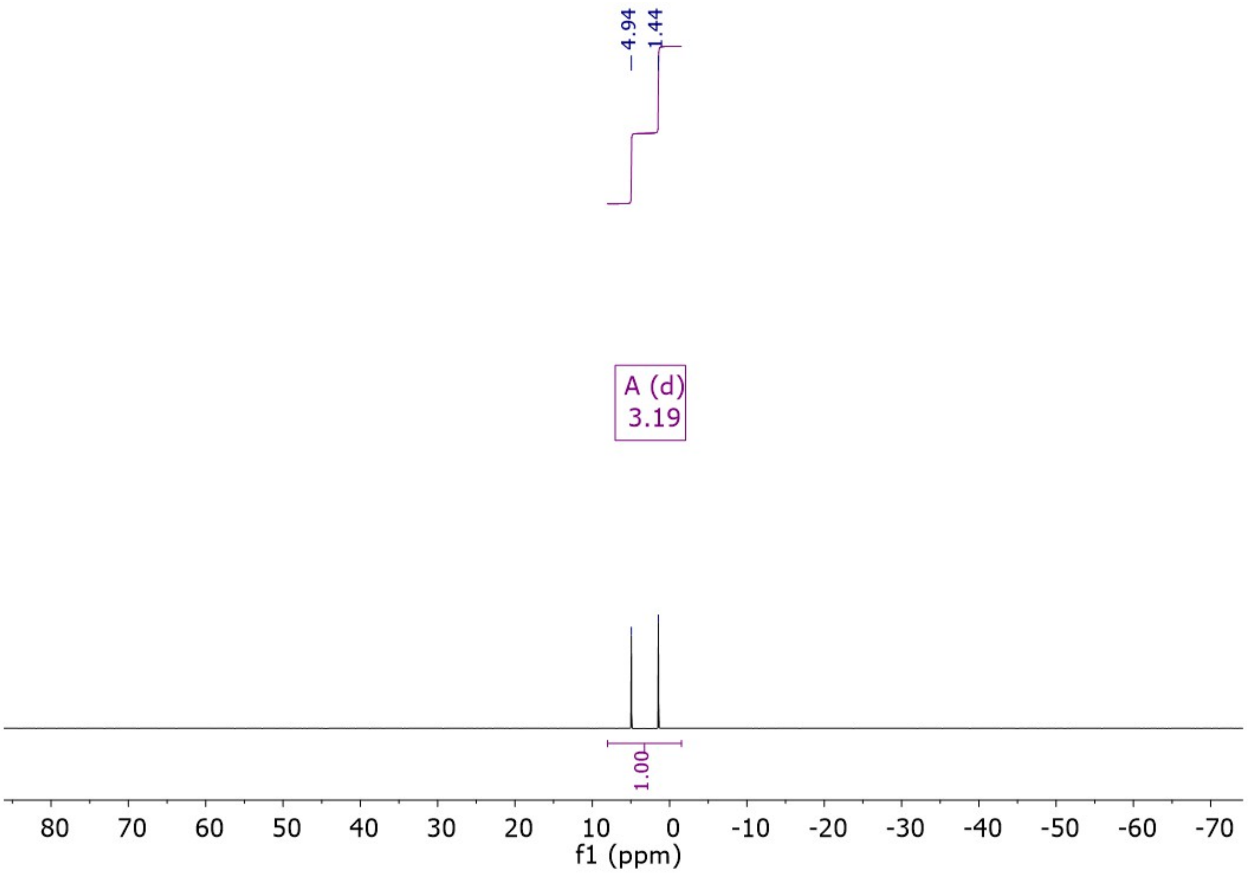

Figure S.39: ^{31}P NMR spectrum (162 MHz, 298 K, $d1$ = 40 s) of the reaction mixture of Na_2HPO_3 with D_2O at pH 13.

S.5 ^{31}P Solid-state NMR Analyses of the Crude Ball-milling Mixtures

S.5.1 Reduction of $Na_3P_3O_9$ with K (Without Dispersant)

The reaction was performed according to the optimized procedure (see S.3.3), just without dispersant (see Figure S.8 for corresponding solution-state ^{31}P NMR spectrum after hydrol-

ysis). The crude mixture was filled into a 3.2 mm ZrO_2 MAS rotor inside of a glovebox, and then brought out for the solid-state NMR measurements.

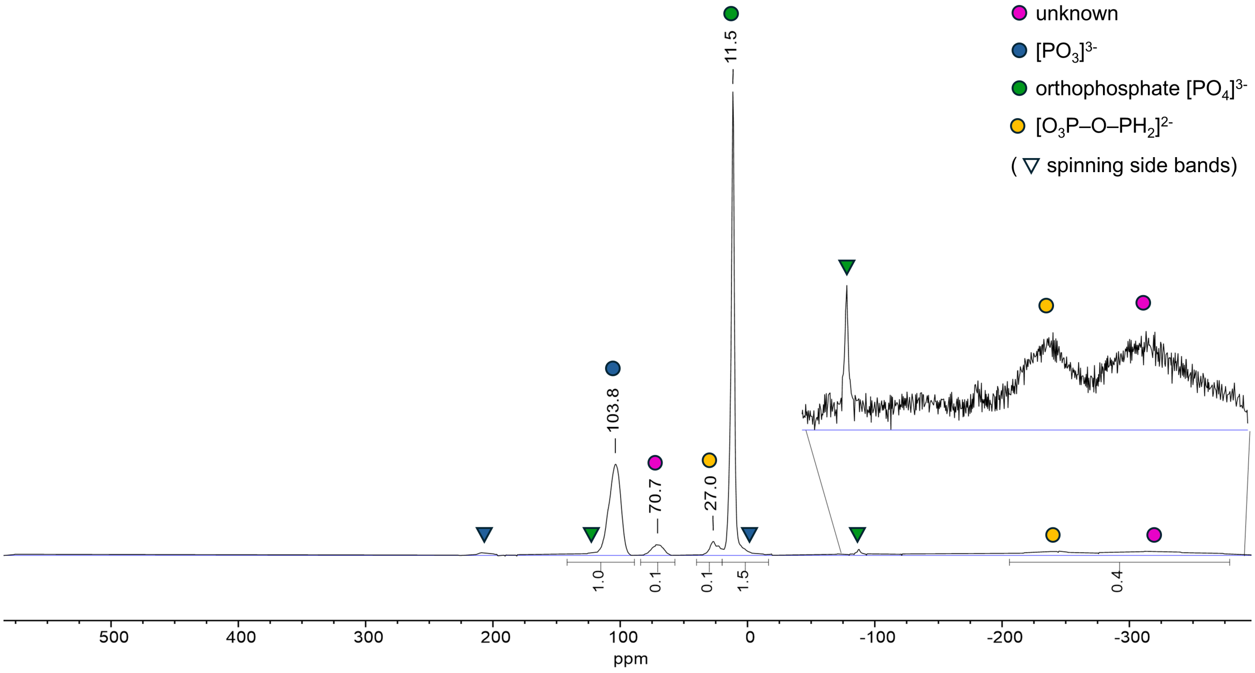

Figure S.40: ^{31}P solid-state NMR spectrum (20 kHz MAS, 202 MHz, 298 K, $d1$ = 4 s) of the reaction mixture of $Na_3P_3O_9$ (1.67 mmol) with K (10.0 mmol, no dispersant) after 12 h at 450 rpm.

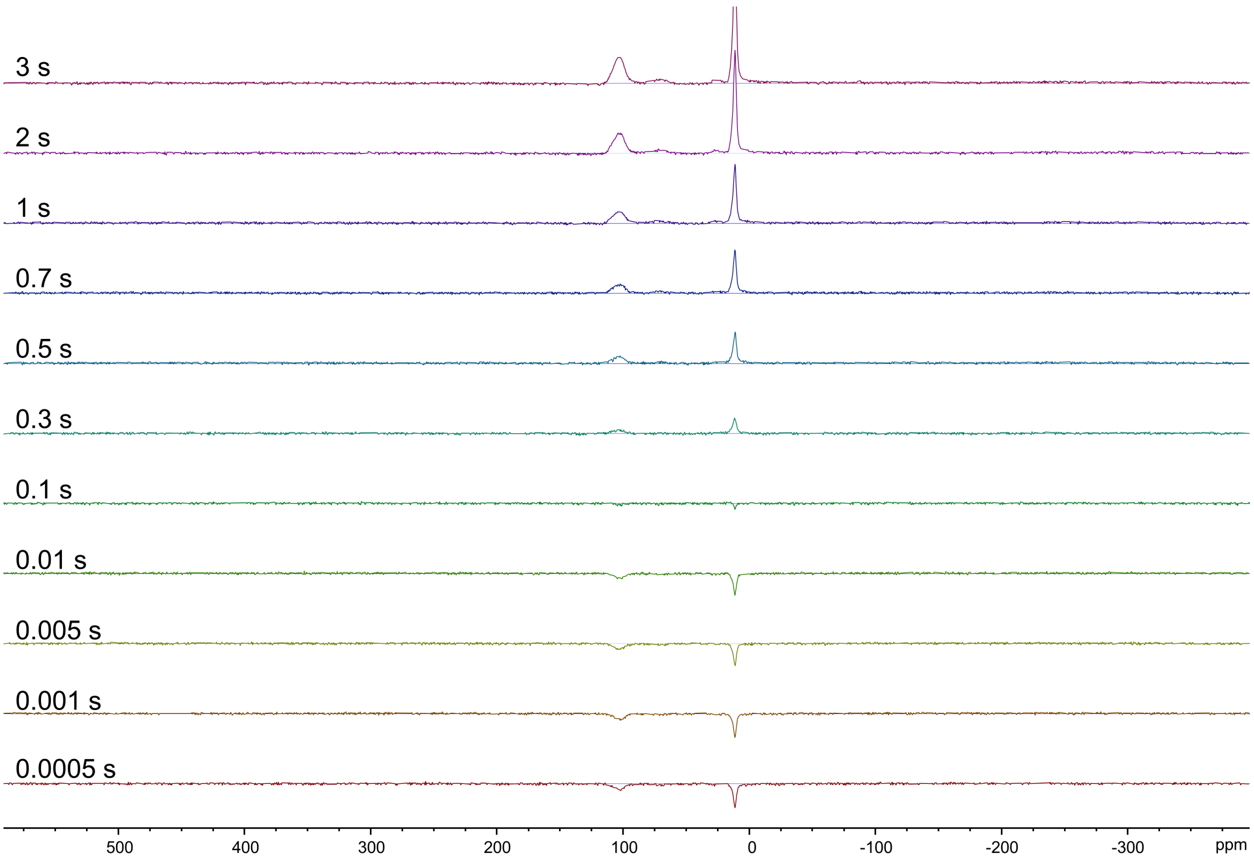

Figure S.41: Measurement of T_1 relaxation times. ^{31}P solid-state NMR spectra (20 kHz MAS, 202 MHz, 298 K) of the reaction mixture of $Na_3P_3O_9$ (1.67 mmol) with K (10.0 mmol, no dispersant) after 12 h at 450 rpm. Values over the spectra represent the corresponding scan delay ($d1$).

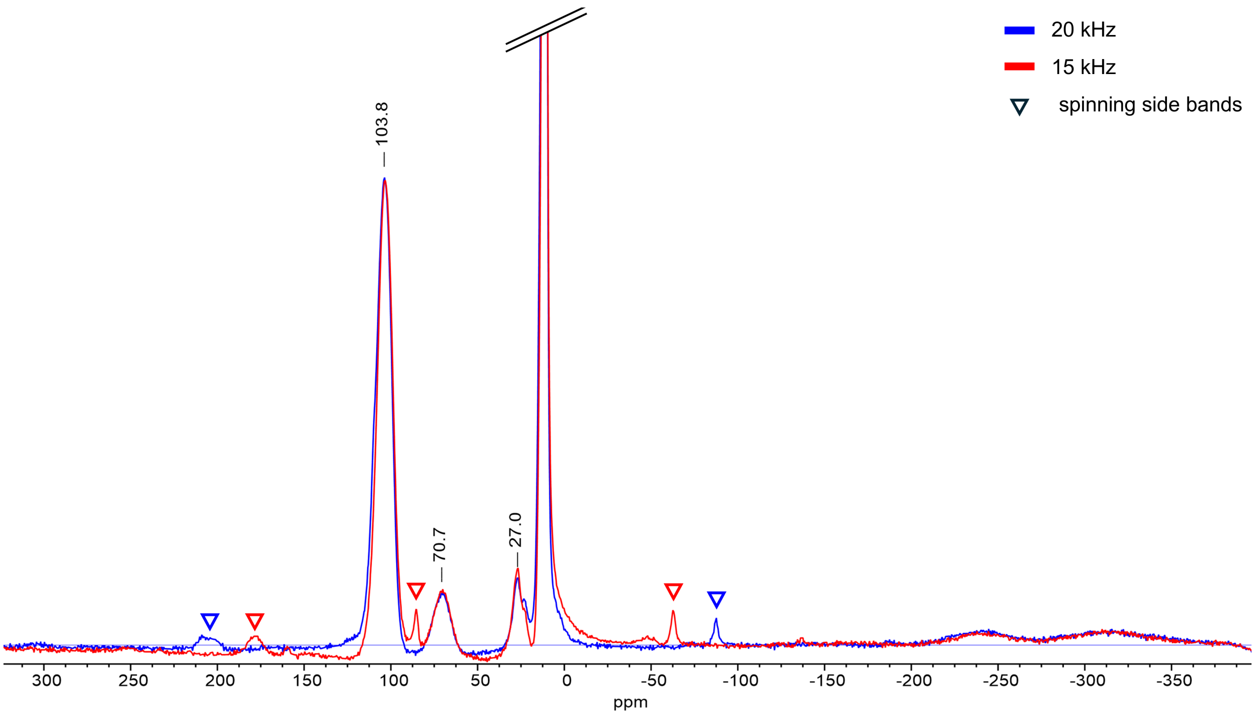

Figure S.42: Comparison of different MAS rotation frequencies. ^{31}P solid-state NMR spectra (202 MHz, 298 K) of the reaction mixture of $Na_3P_3O_9$ (1.67 mmol) with K (10.0 mmol, no dispersant) after 12 h at 450 rpm.

S.5.2 Reduction of $Na_3P_3O_9$ with K and KI

The reaction was performed according to a scale-up experiment of the reduction of $Na_3P_3O_9$ with K and KI (10% w/w K/KI) (see S.10). The crude mixture was filled into a 3.2 mm ZrO_2 MAS rotor inside of a glovebox, and then brought out for the NMR measurements.

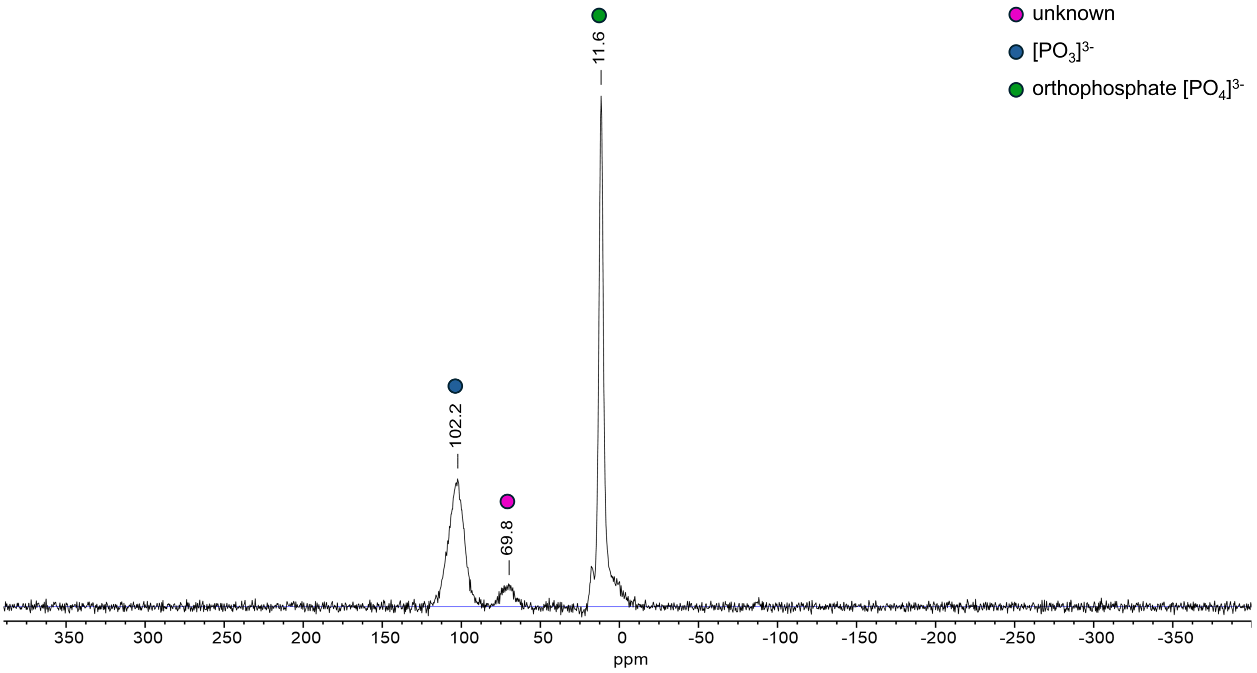

Figure S.43: ^{31}P solid-state NMR spectrum (20 kHz MAS, 202 MHz, 298 K, $d1 = 4$ s) of the reaction mixture prepared by reduction of $Na_3P_3O_9$ with K and KI (10% w/w K/KI) according to a scale-up procedure such as outlined in section S.10.

S.5.3 Exposing $PO_3{}^{3-}$ Containing Crude Material to Air

Inside the glovebox, a portion of the crude product acquired from a scale-up experiment of the reduction of $Na_3P_3O_9$ with K and KI (10% w/w K/KI) (see S.10 for an exemplary procedure and S.5.4 for the solid-state ^{31}P NMR spectrum) was filled into a vial. The vial was brought out of the glovebox and opened. The mixture started to change color from black to grey immediately. When taking out a small portion of the mixture out of a vial with a spatula, the discoloration was immediate and resulted in a white powder. After stirring/shaking the solid for several minutes, the material turned completely white (see Figure S.44). The mixture was loaded into a 3.2 mm ZrO_2 MAS rotor for the solid-state NMR measurements.

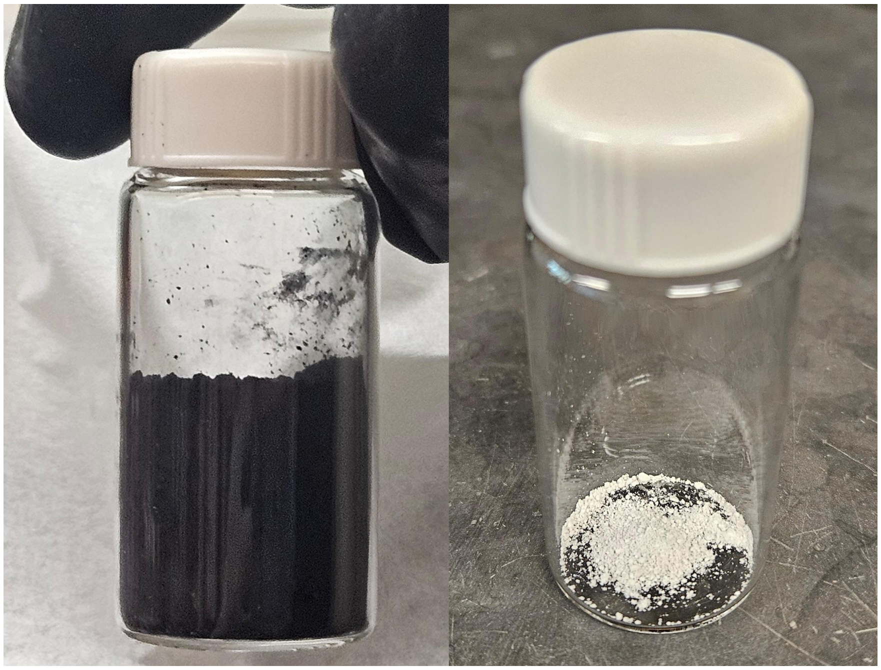

Figure S.44: Left side: isolated product from from the scale-up experiment of the reduction of $Na_3P_3O_9$ with K and KI (10% w/w K/KI) (see S.10). Right side: A portion of this crude product after being exposed to air.

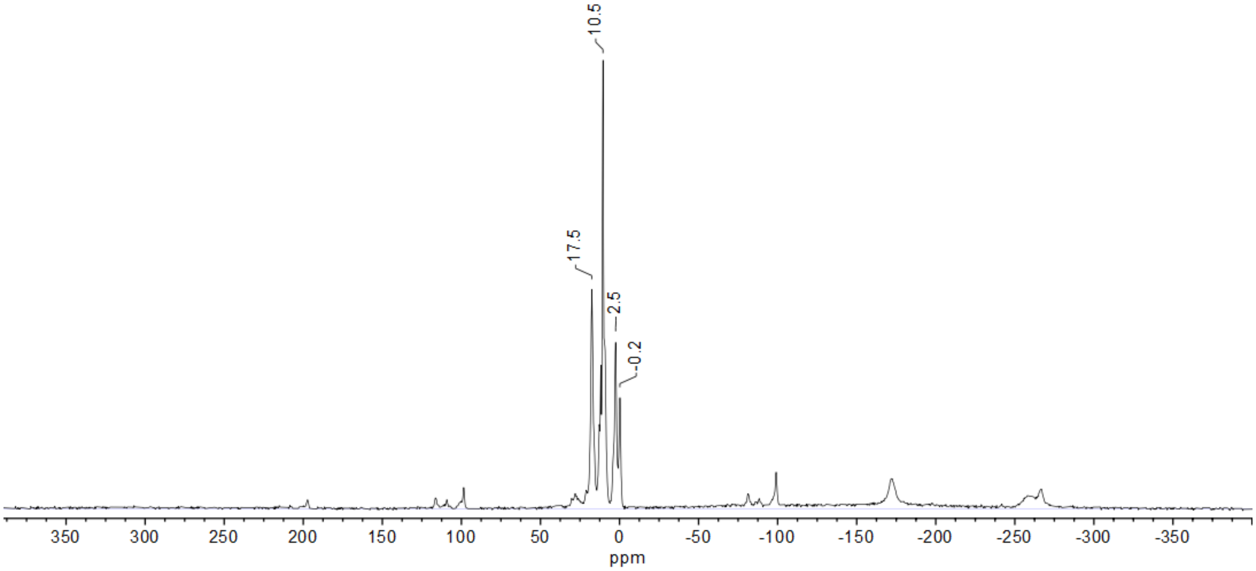

Figure S.45: Solid-state ^{31}P NMR spectrum (20 kHz MAS, 202 MHz, 298 K, $d1 = 4$ s) of the reaction mixture after being exposed to air.

S.5.4 Reduction of Na_2PO_3F with K and KI

The reaction was performed according to the general procedure (see S.1) with the following parameters: Na_2PO_3F (298 mg, 2.07 mmol), K (162 mg, 4.14 mmol, 2 eq.), KI (1458 mg, 10% w/w K/KI), 450 rpm, 12 h. The crude mixture was filled into a 3.2 mm ZrO_2 MAS rotor inside of a glovebox, and then brought out for the NMR measurements.

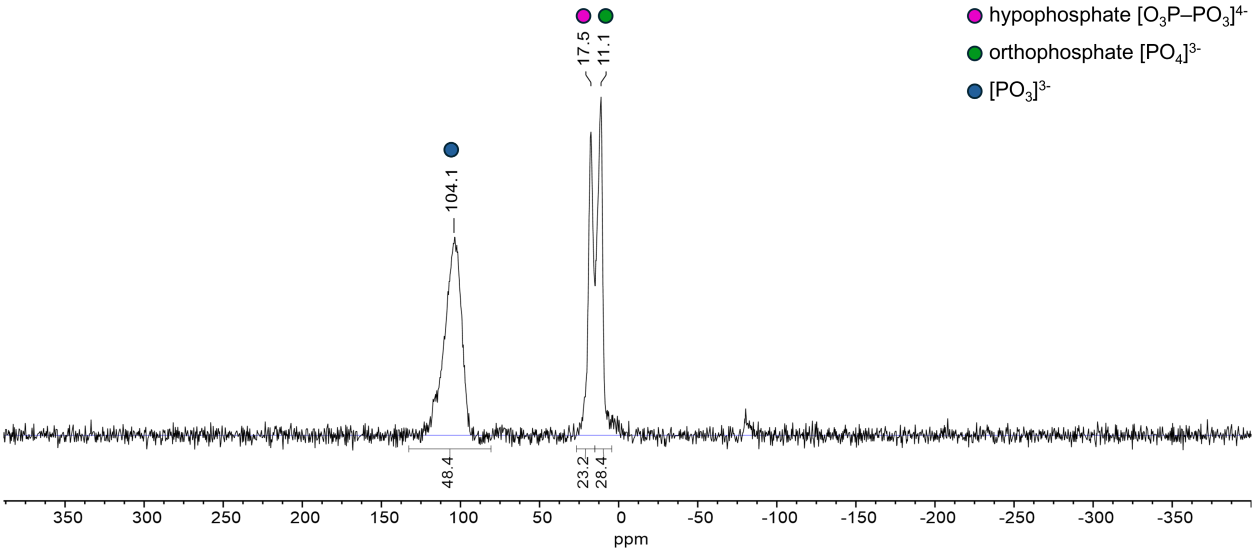

Figure S.46: ^{31}P solid-state NMR spectrum (20 kHz MAS, 202 MHz, 298 K, $d1 = 4$ s) of the reaction mixture prepared according to the above procedure.

S.6 Raman Analyses of the Crude Ball-milling Mixtures

Raman spectra were collected using a LabRAM HR microscope Raman system (Horiba) with a 633 nm excitation laser and a 600 lines/mm grating. The laser was calibrated using a Si(100) wafer (520.5 cm^{-1}) prior to the measurements. Samples and reference compounds were sealed in glass tubes in a nitrogen-filled glovebox and Raman spectra were recorded directly through the glass tubes to prevent air and moisture exposure. Spectra were acquired with acquisition times of 10–15 seconds, averaged over 5 accumulations, using laser powers ranging from 1.7 to 17 mW. Raman spectra were background-subtracted and normalized to the highest peak intensity using LabSpec 6 software (Horiba).

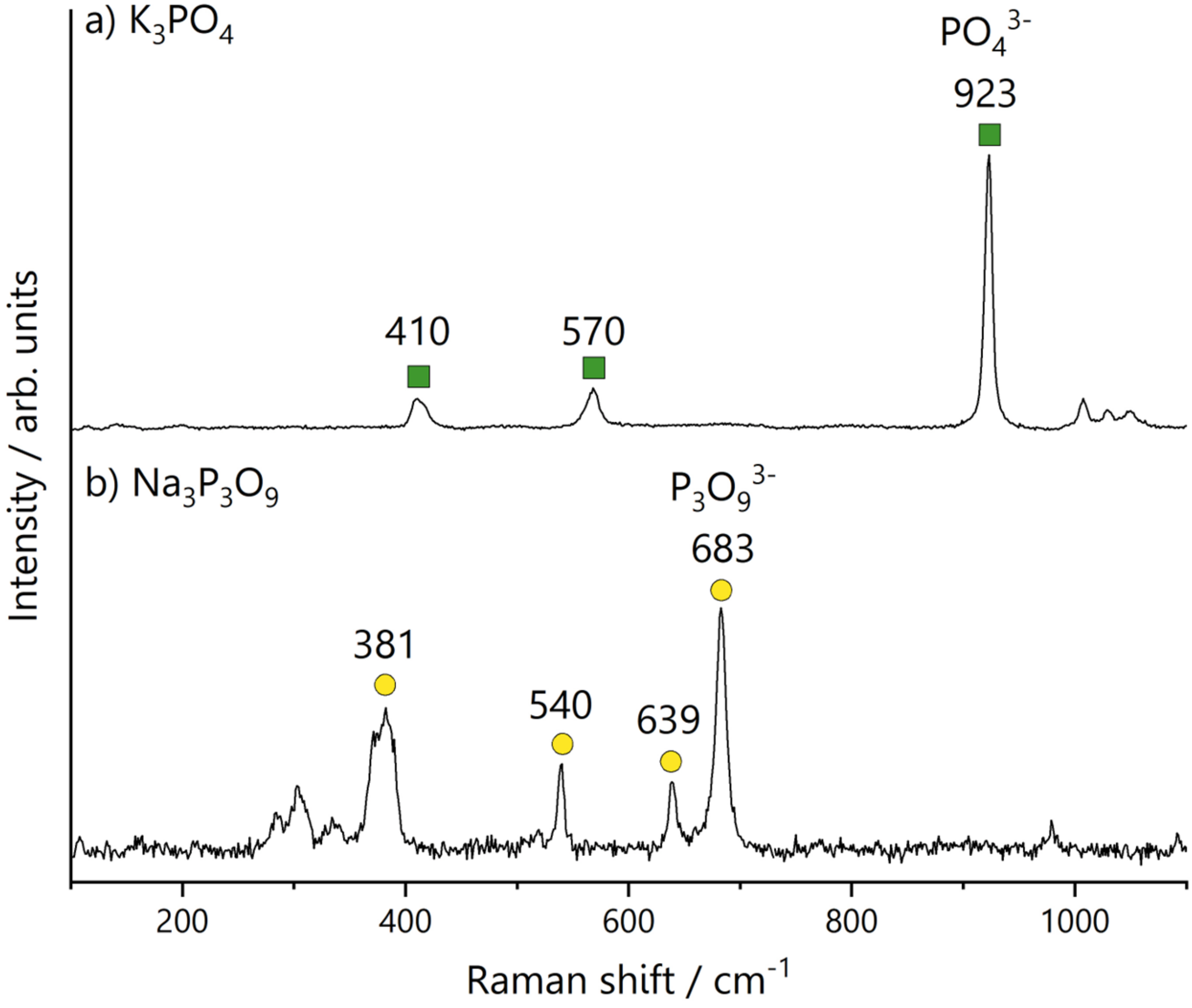

Figure S.47: Raman spectra of reference compounds (acquired with 17 mW laser power). a) K_3PO_4 shows characteristic peaks associated with the PO_4^{3-} (orthophosphate) group, including peaks at 410 cm^{-1} (symmetric bending mode), 570 cm^{-1} (asymmetric bending mode), and 923 cm^{-1} (symmetric stretching mode of the P–O bond). b) $Na_3P_3O_9$ shows characteristic peaks associated with the cyclic $P_3O_9^{3-}$ (metaphosphate) group, including peaks at 381 cm^{-1} (P–O–P symmetric bending), 540 cm^{-1} (P–O–P symmetric bending), 639 cm^{-1} (P–O–P symmetric bending deformation or ring breathing), and 683 cm^{-1} (P–O–P symmetric bending asymmetric stretching).

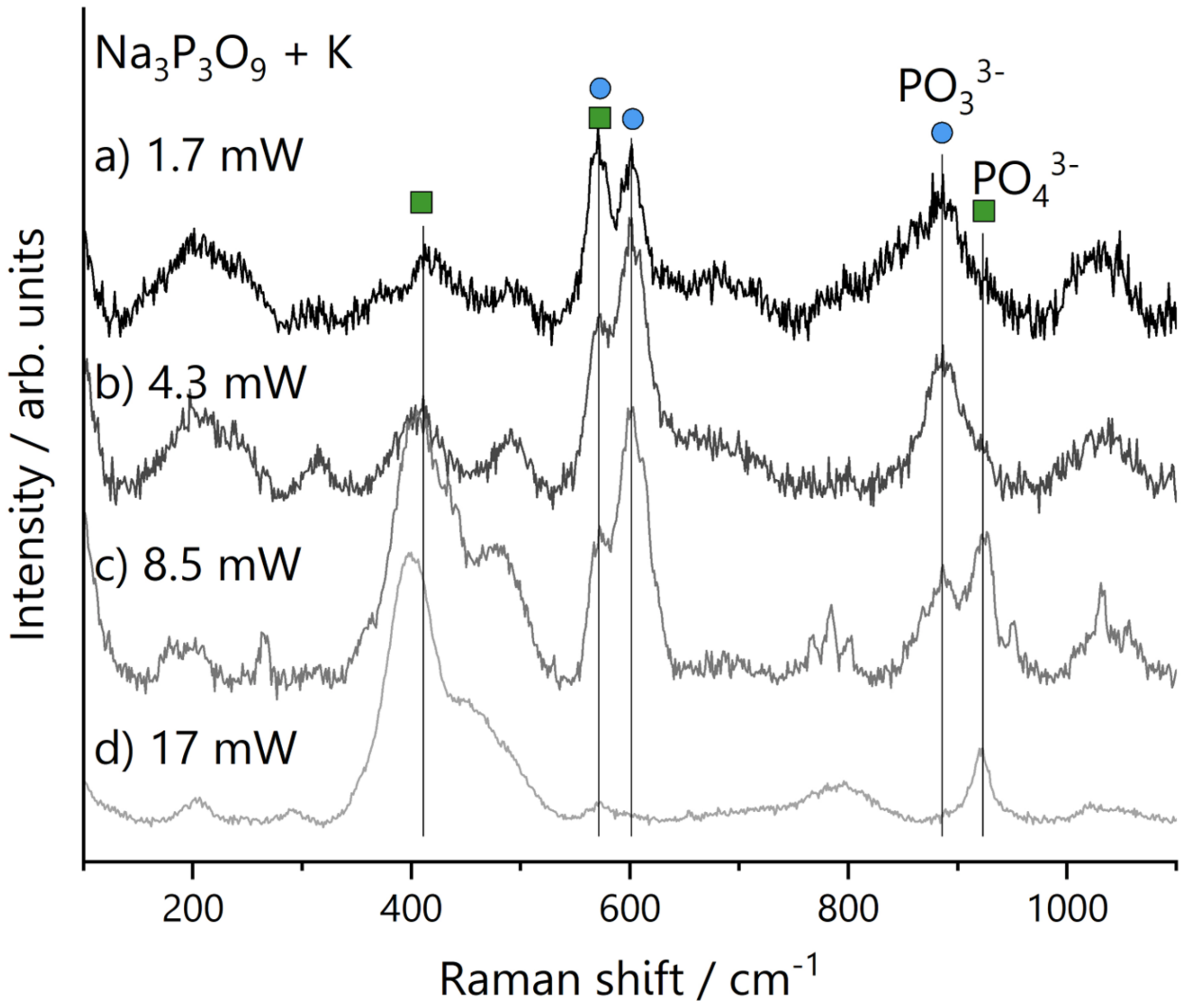

Figure S.48: Raman spectra at different laser powers from 1.7 to 17 mW (a–d) of a mechanochemical reaction mixture ($Na_3P_3O_9$ + K, no dispersant). Each spectrum was collected at a different sample position. Increasing laser power leads to an intensification of $PO_4{}^{3-}$-associated Raman bands, while those attributed to $PO_3{}^{3-}$ diminish. This behavior may result from localized laser-induced heating, which either causes preferential degradation of $PO_3{}^{3-}$-enriched surface regions or promotes thermally driven oxidation or conversion of $PO_3{}^{3-}$ to $PO_4{}^{3-}$.

S.7 Detection of PH_3 after PO_3^{3-} Containing Crude Material Hydrolysis/Methanolysis

Inside of the glovebox, the crude mixture from the reduction of $Na_3P_3O_9$ with K (ca. 9 mg, synthesized according to the optimized procedure (see S.3.3, just without dispersant)) was filled into a J-Young type NMR tube. The NMR tube was closed, brought into the fume-hood, and the lower part of the tube was submerged in liquid nitrogen. The cap was briefly opened to add deionized water via a syringe, and then closed again. The NMR tube was then taken out of the liquid nitrogen and allowed to warm up (see Figure S.49). ***Danger! A pressure build up to up to 1 bar is expected!*** Then the tube was shaken and examined via ^{31}P NMR spectroscopy. The same procedure was repeated with (wet) methanol instead of water. See Figures S.50 and S.51 for the spectra.

PH_3 was detected in the ^{31}P NMR spectrum after methanolysis. Note that a quantification was not possible with this method. In contrast to methanolysis, after hydrolysis, no PH_3 was detected in the ^{31}P NMR spectrum. A possible reason for this could be the low solubility of PH_3 in water.

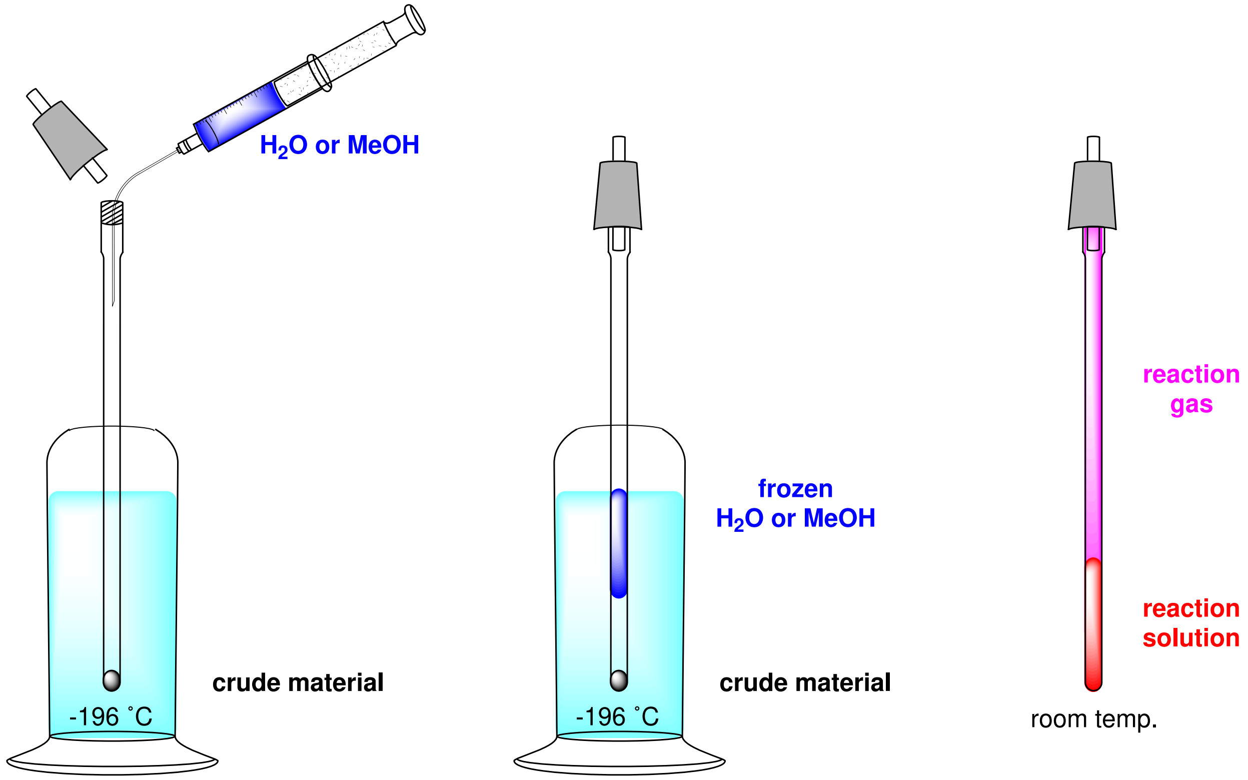

Figure S.49: Illustration of the gas-capturing method employed for the PH_3 detection via ^{31}P NMR spectroscopy.

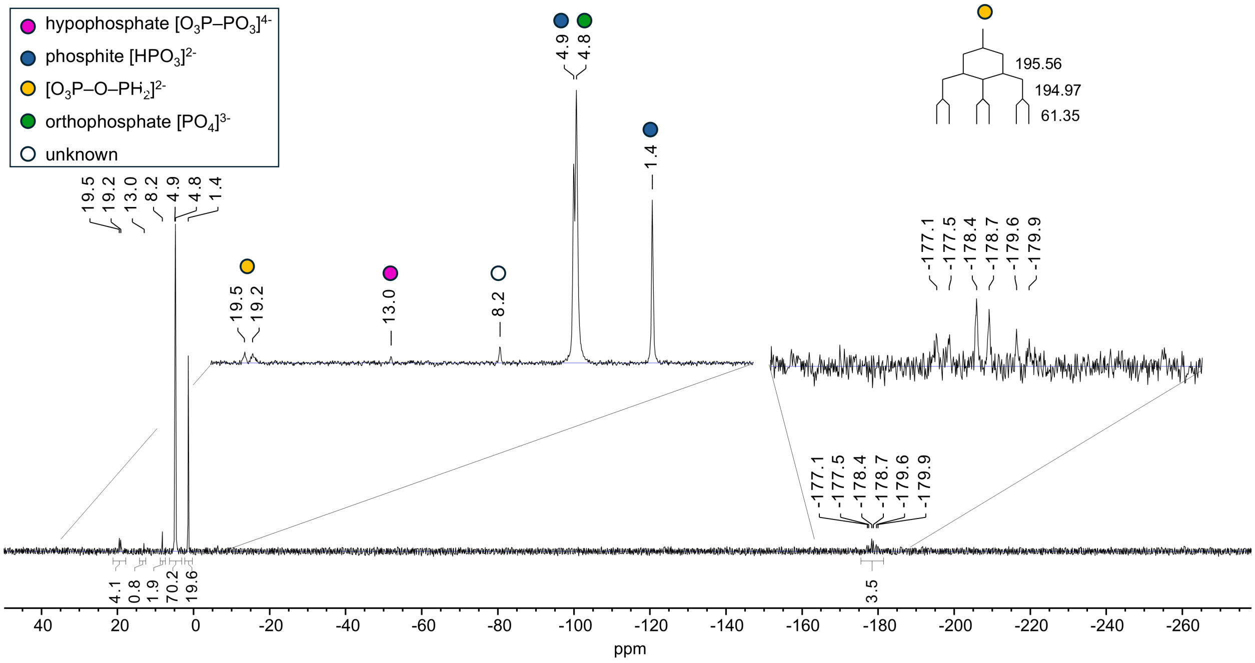

Figure S.50: ^{31}P NMR spectrum (162 MHz, H_2O, $d1 = 40$ s, 298 K) of the hydrolysis reaction of the crude mixture in a closed J-Young type NMR tube for gas capturing.

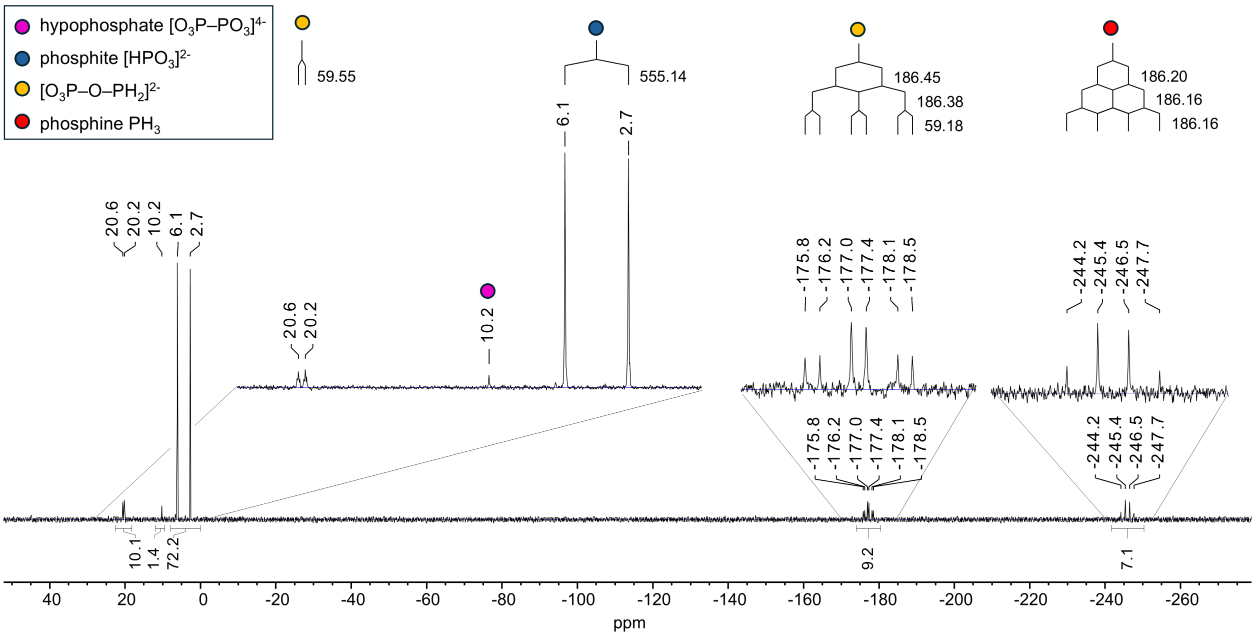

Figure S.51: ^{31}P NMR spectrum (162 MHz, CH_3OH, $d1 = 40$ s, 298 K) of the methanolysis reaction of the crude mixture in a closed J-Young type NMR tube for gas capturing.

S.8 ICP-OES Analysis of Crude Material after Hydrolysis

ICP-OES measurements were carried out using an Agilent 5100 ICP-OES equipped with Dual View (DV). Standard solutions (Sigma-Aldrich) and samples were prepared in 2% HNO_3 and filtered through 0.2 µm syringe filters prior to analysis. Elemental quantification was performed using optimized wavelengths to ensure high sensitivity and minimal spectral interference. Calibration curves were generated from multi-element standards at 0 ppm, 1 ppm, 5 ppm, 40 ppm, and 45 ppm in 2% HNO_3.

The 8 mg of the crude mixture derived from the reaction of ($Na_3P_3O_9$ + K, no dispersant) was digested in 50 mL of 2% HNO_3 to prepare the stock solution. Then, 1.25 mL of this stock solution was further diluted with 8.75 mL of 2% HNO_3 to prepare the final sample for ICP-OES analysis.

Table S.2: ICP-OES elemental analysis and normalization to phosphorus

**Element**	**ICP-OES values (ppm)**	**Real ppm**	**mmol/kg**	**Norm. to P**
K	8.22	51.38	1.313939	2.120791
Na	2.53	15.81	0.687799	1.110157
P	3.07	19.19	0.619551	1.000000

The approximate formula based on mole normalization is K_2NaPO_3. These results are consistent with the expected stoichiometric ratio between phosphorus, sodium, and potassium atoms. The excess amounts of Na and K observed (see Table S.2) can be attributed to the loss of PH_3 during the hydrolysis (see Section S.7).

S.9 Assessment of Oxygen Balance and Phosphorus Redox States

For a full assessment of the overall P:O ratio and the average P oxidation states, the calculated yields of the corresponding ^{31}P NMR data of the aqueous workups of exemplary reactions were used. Additionally, the amount of PH_3 was calculated as 100% minus the recovered total yield of phosphorus-containing species. The results are summarized in Tables S.3, S.4, and S.5 (see below).

Table S.3: Phosphorus species distribution, oxidation states, and oxygen atom counts for the reaction of $\mathbf{Na_3P_3O_9}$ (1.67 mmol) with K (10.0 mmol) and KI (3.52 g, K/KI ratio 10% w/w) after 12 h at 450 rpm. See Figure S.10 for the ^{31}P NMR spectrum.

**Species**	PO_4^{3-}	HPO_3^{2-}	$[O_3P–PO_3]^{4-}$	$[O_3P–PH_2]^{2-}$	PH_3	Total/Avg.
**Yield (%)**	44.1	31.6	5.4	3.7	11.5	100
**Oxidation State**	+5	+3	+4	−1	−3	+3.17
**O:P ratio**	4	3	3	0.5	0	3.02

Table S.4: Phosphorus species distribution, oxidation states, and oxygen atom counts for the reaction of $\mathbf{Na_2PO_3F}$ (2.07 mmol) with K (4.14 mmol) and KI (1.46 g, K/KI ratio 10% w/w) after 12 h at 450 rpm. See Figure S.15 for the ^{31}P NMR spectrum. *Slight deviation due to a minor unidentified species that has been taken into account for the total yield

**Species**	PO_4^{3-}	HPO_3^{2-}	$[O_3P–PO_3]^{4-}$	$[O_3P–PH_2]^{2-}$	PH_3	Total/Avg.
**Yield (%)**	25.9	44.1	16.0	1.9	9.5*	99.3*
**Oxidation State**	+5	+3	+4	−1	−3	+3.07
**O:P ratio**	4	3	3	0.5	0	2.94

Table S.5: Phosphorus species distribution, oxidation states, and oxygen atom counts for the reaction of $\mathbf{KPO_3}$ (5.00 mmol) with K (10.0 mmol) and KI (3.52 g, K/KI ratio 10% w/w) after 24 h at 450 rpm. See Figure S.17 for the ^{31}P NMR spectrum.

**Species**	PO_4^{3-}	HPO_3^{2-}	$[O_3P–PO_3]^{4-}$	$[O_3P–PH_2]^{2-}$	PH_3	Total/Avg.
**Yield (%)**	36.1	36.0	9.5	3.7	11.0	100
**Oxidation State**	+5	+3	+4	−1	−3	+3.08
**O:P ratio**	4	3	3	0.5	0	2.96

S.10 Scale-up of the PO_3^{3-} Salt Synthesis

The reduction of $Na_3P_3O_9$ with K and KI was scaled up several times with varying loadings of material and jar sizes. The procedure was based on the general procedure (see S.1), but scaled up accordingly. The largest successful scale-up reactions contained 5.53 mmol and 8.33 mmol $Na_3P_3O_9$, respectively. Herein, the largest scale-up is described in detail (the changed parameters are bold):

Procedure: Inside the glovebox, $Na_3P_3O_9$ (**2.55 g**, **8.33 mmol**, 1.00 eq.), potassium (**1.96 g**, **50.0 mmol**, 6.00 eq., **six** freshly cut chunks), the dispersant (**17.6 g**) and **100** stainless steel balls (∅ 10 mm) were added into a **500 mL** ball-milling jar. The jar was sealed and brought out. The mixture was ball milled at 200 rpm for **15 min**, then 450 rpm for 12 h (with cooling breaks of 30 min every 1 h and direction change every cycle, total process time 24 h). When the desired grinding time was reached, the jar was brought into the glovebox and opened. The reaction mixture was isolated in a vial.
Yield: Theoretically produced crude material: 22.1 g. Isolated crude material: 18.8 g (85 %). The composition of the scaled up reaction product is similar to the original procedure with somewhat increased PO_3^{3-} yield (38 %).

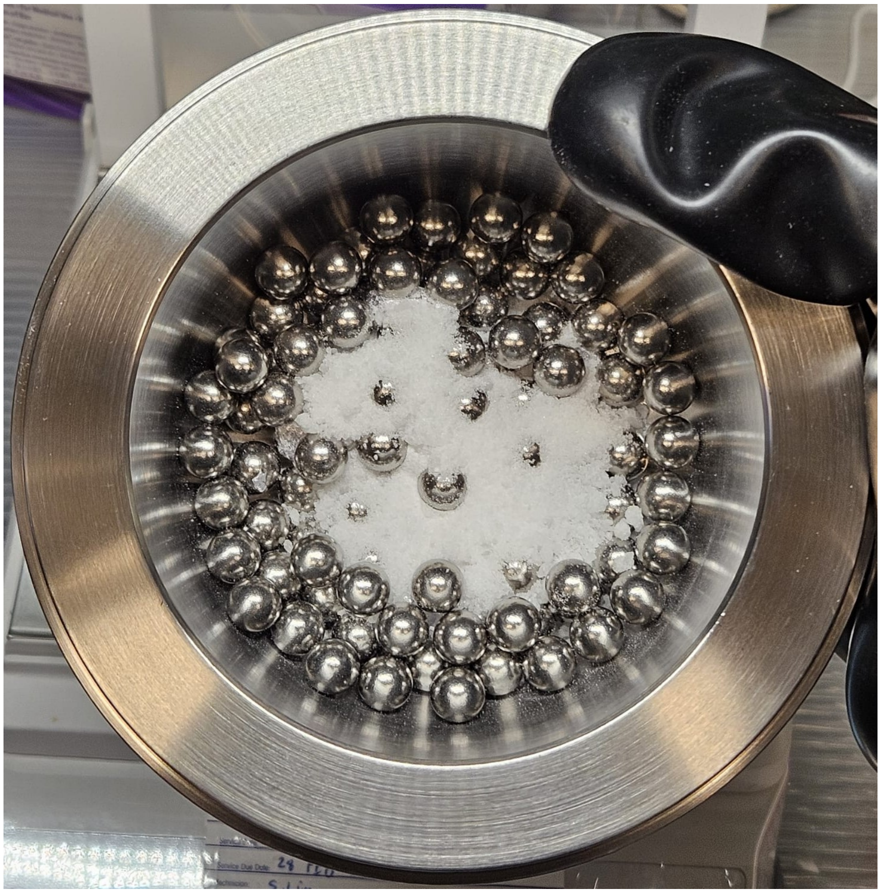

Figure S.52: Ball-milling jar before the reaction, after filling it with the reactants.

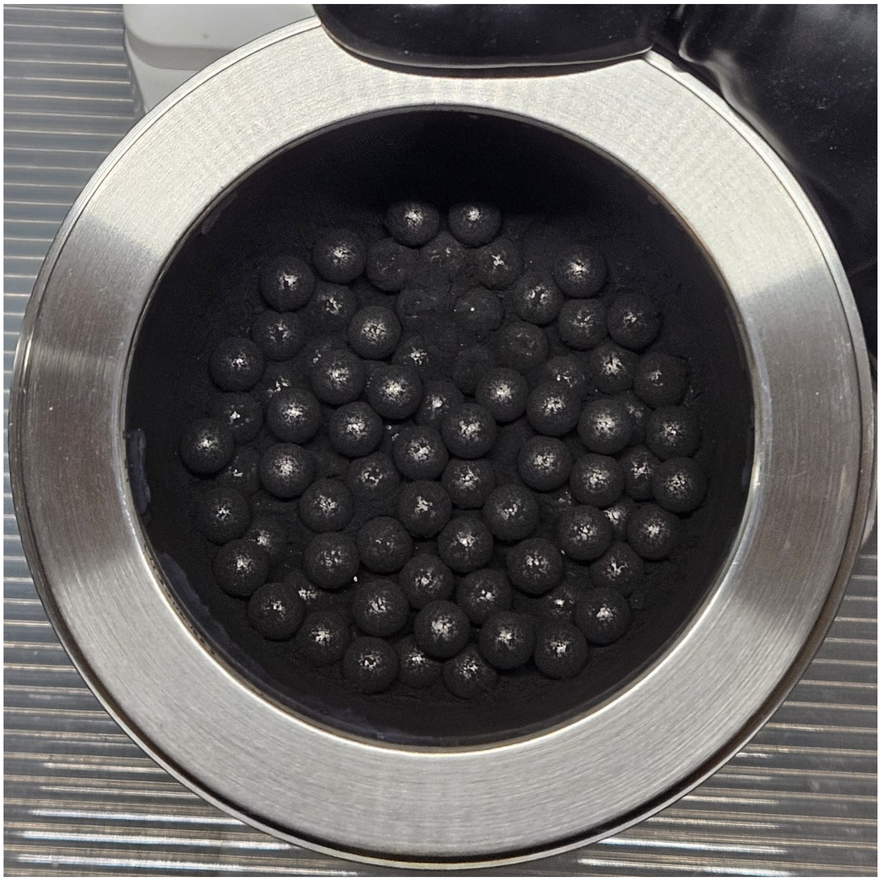

Figure S.53: Ball-milling jar after the reaction, containing the crude product.

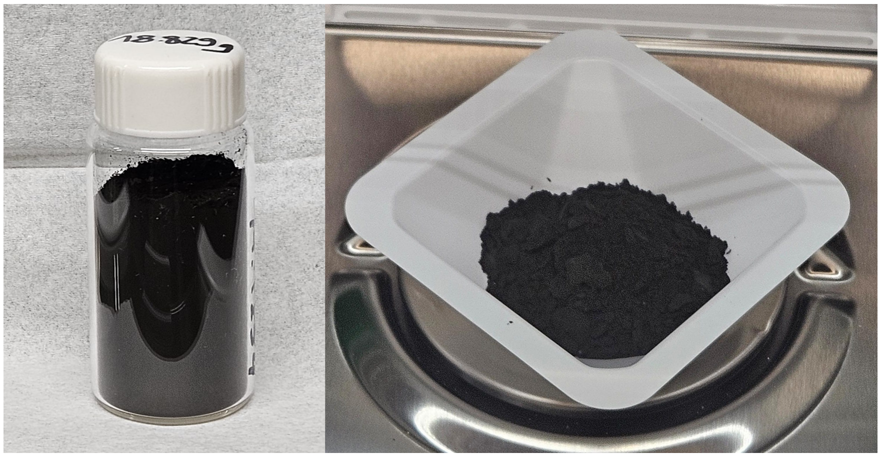

Figure S.54: Vial containing the isolated crude material (18.8 g, left). 15 g aliquot of the crude material in a weighing boat (right).

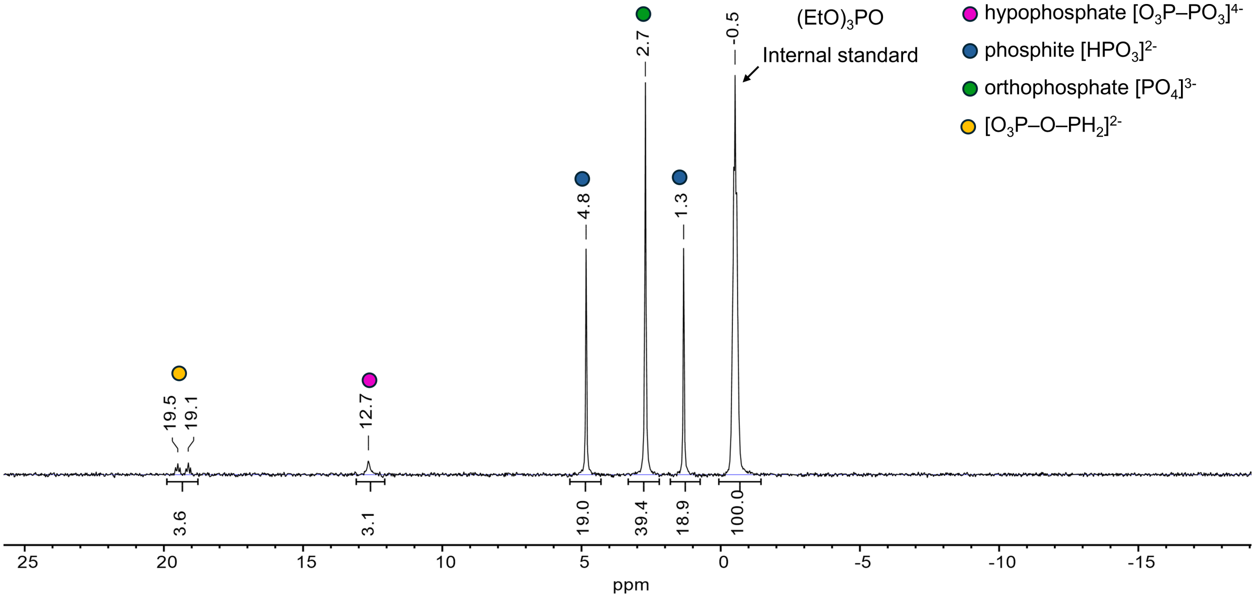

Figure S.55: ^{31}P NMR spectrum (162 MHz, H_2O/D_2O, $d1 = 40$ s, 298 K) of the (scale-up) reaction mixture of $Na_3P_3O_9$ (8.33 mmol) with K (50.0 mmol) and KI (17.6 g, K/KI ratio 10% w/w) after 12 h. Aliquot taken: 385 mg. $OP(OEt)_3$ (80 mg) was added as internal standard.

S.11 Reactivity of the Crude $PO_3{}^{3-}$ Salt

S.11.1 Synthesis of Phosphite ($HPO_3{}^{2-}$) Salts

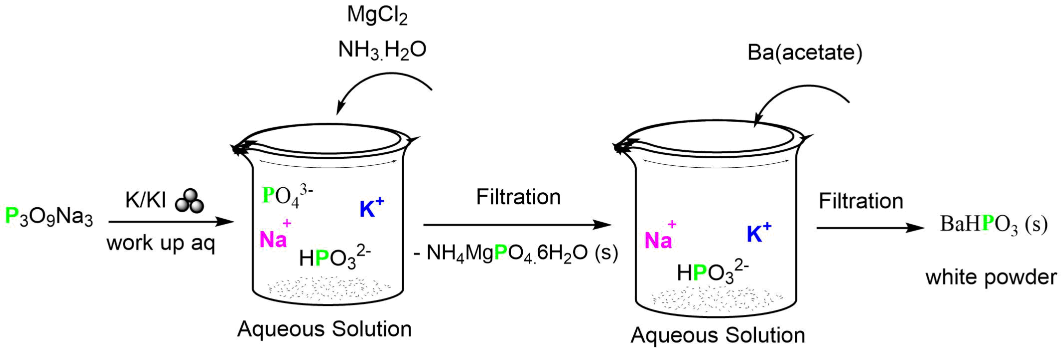

Figure S.56: Isolation process of phosphite as $BaHPO_3{\cdot}H_2O$.

Procedure: The crude mixture containing $PO_3{}^{3-}$ (300 mg) derived from the reaction of $Na_3P_3O_9$ with K (no dispersant) was hydrolyzed by degassed water under nitrogen. The purification was conducted according to a previously reported procedure.[4] Magnesium chloride (278.25 mg, 1.3 eq.) and ammonium hydroxide (28% w/w, 0.75 mL, 4.5 eq.) were added, resulting in the rapid formation of a white precipitate, presumably $NH_4MgPO_4{\cdot}6H_2O$. The slurry was stirred for 5 min and filtered through filter paper. The removal of orthophosphate was confirmed by analyzing an aliquot of the filtrate by ^{31}P NMR spectroscopy. Barium acetate (0.6 g, 1.0 eq.) was then added to the filtrate, and the formed barium phosphite ($BaHPO_3{\cdot}H_2O$) was collected on a frit (15 mL, medium porosity), transferred to a vial, and dried at 120 °C until a constant mass was achieved, providing $BaHPO_3{\cdot}H_2O$ (78 mg, 66%).[7]

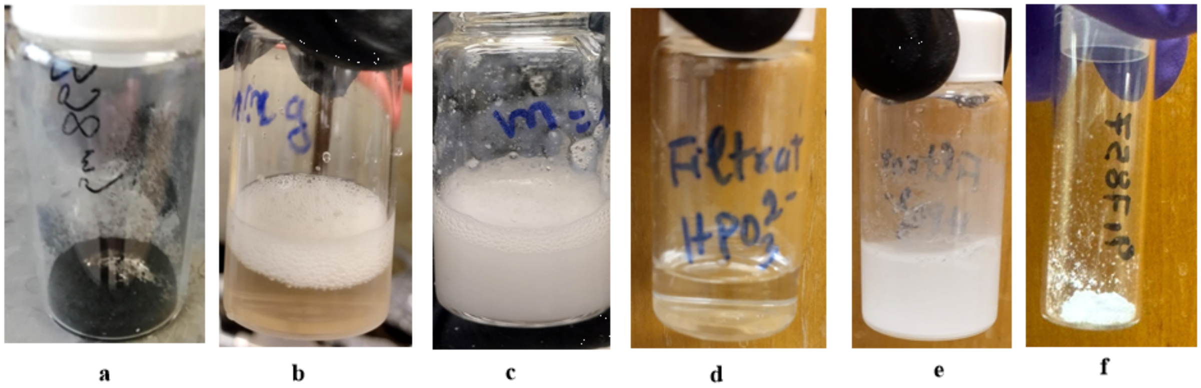

Figure S.57: Procedure for isolating $BaHPO_3{\cdot}H_2O$. a: crude mixture $PO_3{}^{3-}$. b: after hydrolysis with H_2O. c: after adding $MgCl_2$ and NH_3. d: mixture solution after filtration. e: after adding barium acetate. f: dried $BaHPO_3{\cdot}H_2O$.

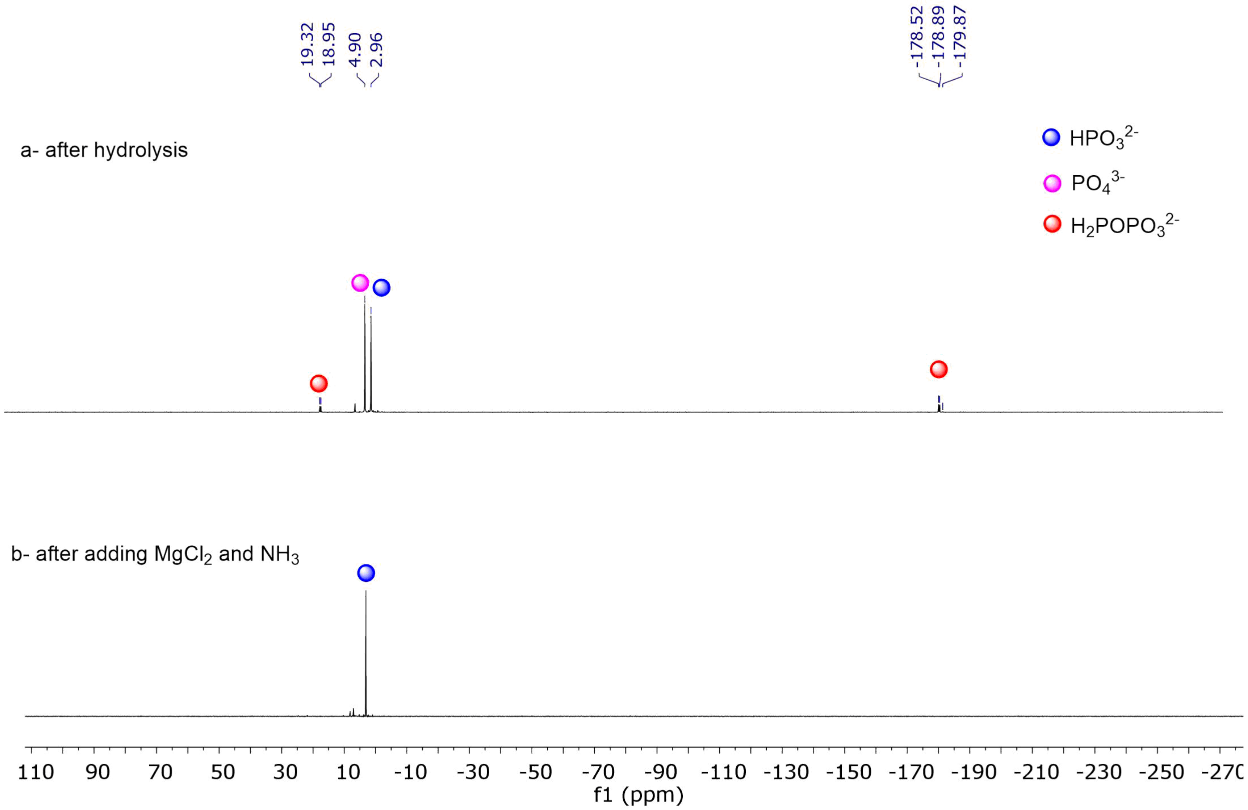

Figure S.58: $^{31}P\{^1H\}$ NMR spectrum (162 MHz, H_2O/D_2O, $d1$ = 20 s, 298 K) a) after hydrolysis and b) after adding $MgCl_2$ and NH_3.

S.11.2 Synthesis of $P(OSiMe_3)_3$

S.11.2.1 In THF

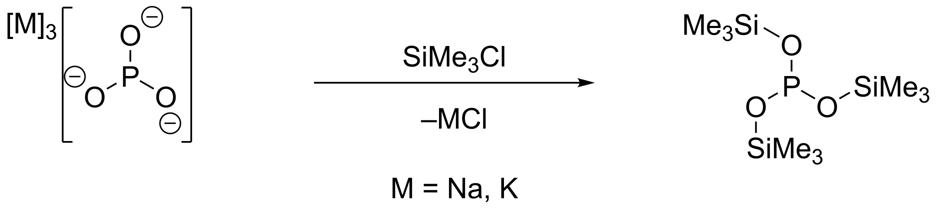

Figure S.59: Synthesis of tris(trimethylsilyl)phosphite.

Reaction: Inside the glovebox, the crude ball-milling mixture (3.31 g, containing 1.19 mmol PO_3^{3-} (synthesized from $Na_3P_3O_9$ + K/KI, 10 w/w % K/KI (pre-dispersed), assuming 32% PO_3^{3-} yield, synthesized according to S.3.3) was loaded into a 100 mL Schlenk flask and suspended in 35 mL THF. Afterwards, TMSCl (3.50 mL, 27.6 mmol) was added. The suspension was vigorously stirred for 5 h. An aliquot was analyzed by NMR spectroscopy, showing a mixture of $P(OTMS)_3$ and $OP(OTMS)_3$. The suspension was filtered through Celite®, and all volatile materials were removed from the filtrate.

Distillation workup: The crude product was distilled at 1000 mTorr (1.33 mbar). The oil bath was set to 55 °C. After several minutes, the distillation head temperature reached 30 °C, which resulted in transfer of a colorless liquid. After the distillation head temperature started to decrease again, the distillation was stopped immediately. The receiving vial was brought back into the glovebox. The purified product was isolated by first pipetting it into a vial and then flushing out the rest with THF. Afterwards, the THF was removed again in vacuo.

Yield: 162 mg (0.543 mmol, 46%)

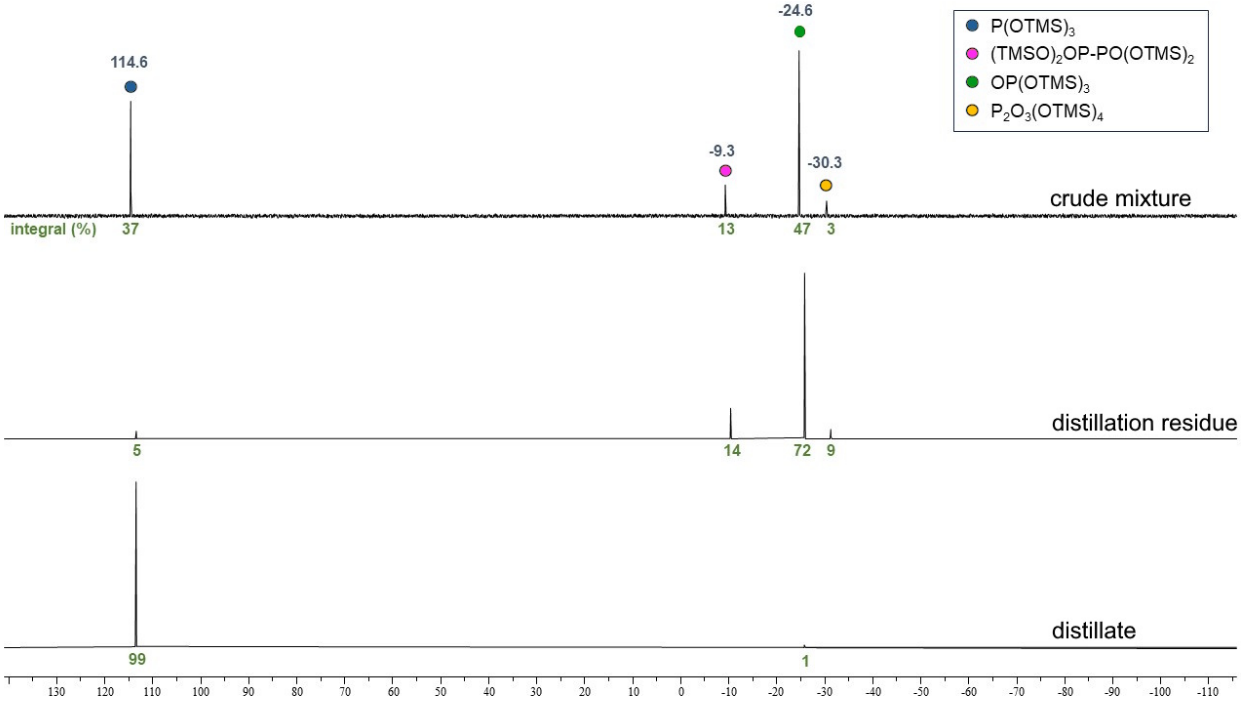

Figure S.60: $^{31}P\{^{1}H\}$ NMR spectrum (162 MHz, THF, $d1$ = 20 s, 298 K) of the crude mixture, the distillation residue, and the distillate. The green numbers are the respective integrals. Note that $P(SiMe_3)_3$ was not observed under these conditions.

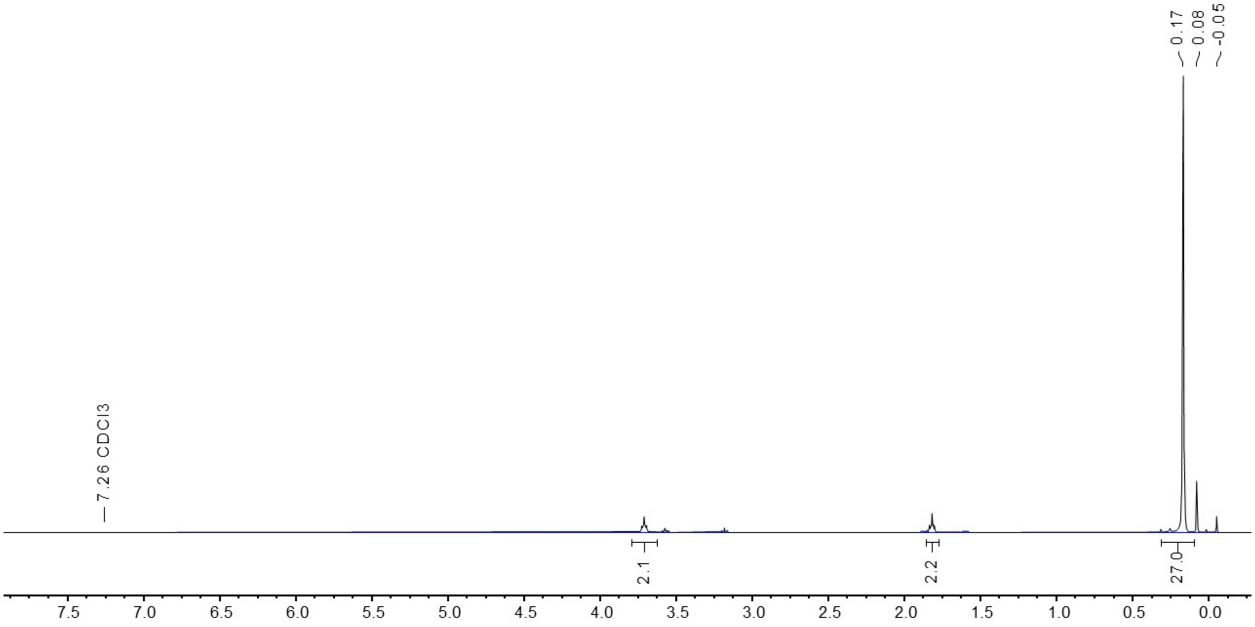

Figure S.61: ^{1}H NMR spectrum (400 MHz, $CDCl_3$, $d1$ = 1 s, 298 K) of the distillate.

S.11.2.2 Without Solvent

Reaction: Inside the glovebox, the crude ball-milling mixture (2.79 g, containing 1.17 mmol PO_3^{3-} (synthesized from $Na_3P_3O_9$ + K + KI, 10 w/w % K/KI, assuming 37% PO_3^{3-} yield, synthesized in a scale-up reaction according to S.10) was loaded into a 50 mL Schlenk flask. Afterwards, TMSCl (3.0 mL, 23.6 mmol) was added. The Schlenk flask was tightly closed and brought out of the glovebox. The suspension was vigorously stirred for 23 h at 100 °C. ***Warning, elevated pressure! Use a blast shield!*** After the mixture was allowed to cool down, the flask was brought into the glovebox again. An aliquot was diluted with THF and analyzed by ^{31}P NMR spectroscopy. The product was not isolated.

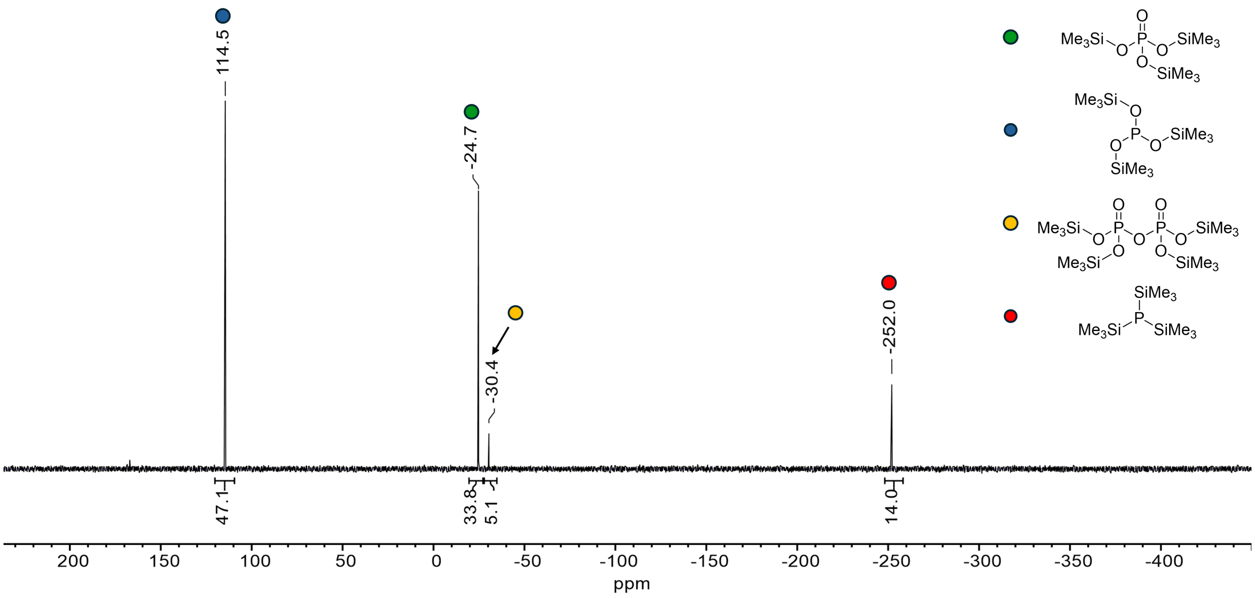

Figure S.62: ^{31}P NMR spectrum (162 MHz, THF, $d1$ = 40 s, 298 K) of the crude mixture.

S.12 Preliminary Reactivity Studies

S.12.1 Generation of Dimethyl Methylphosphonate (DMMP)

Methylation of the crude mixture containing PO_3^{3-} gave dimethyl methylphosphonate (DMMP), which is used as a flame-retardant.[8,9] It was obtained in approximately 28% ^{31}P NMR-yield in a mixture with trimethyl phosphate and was not isolated.

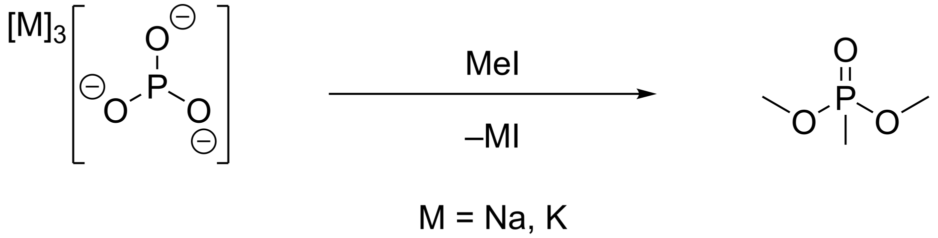

Figure S.63: Synthesis of dimethyl methylphosphonate.

Reaction: Inside the glovebox, the crude ball-milling mixture (100 mg, containing 0.036 mmol PO_3^{3-} (synthesized from $Na_3P_3O_9$ + K + KI, 10 w/w % K/KI, assuming 32% PO_3^{3-} yield, synthesized according to S.3.3) was filled into a J-Young type NMR tube. Afterwards, C_6D_6 (0.4 mL) and an excess of methyl iodide (0.1 mL) were added, and the NMR tube was tightly closed with a Teflon screw cap. The NMR tube was taken out of the glovebox and then heated at 150 °C for three hours. Afterwards, the NMR tube was cooled down to room temperature and analyzed *via* $^{31}P\{^1H\}$ NMR spectroscopy. The product was not isolated.

Yield determination: The NMR tube was brought back into the glovebox. Ph_3PO (13.9 mg, 0.0499 mmol) was added as internal standard. The NMR tube was taken out again and analyzed *via* quantitative $^{31}P\{^1H\}$ NMR spectroscopy. Calculated yield: 0.0101 mmol, 28%.

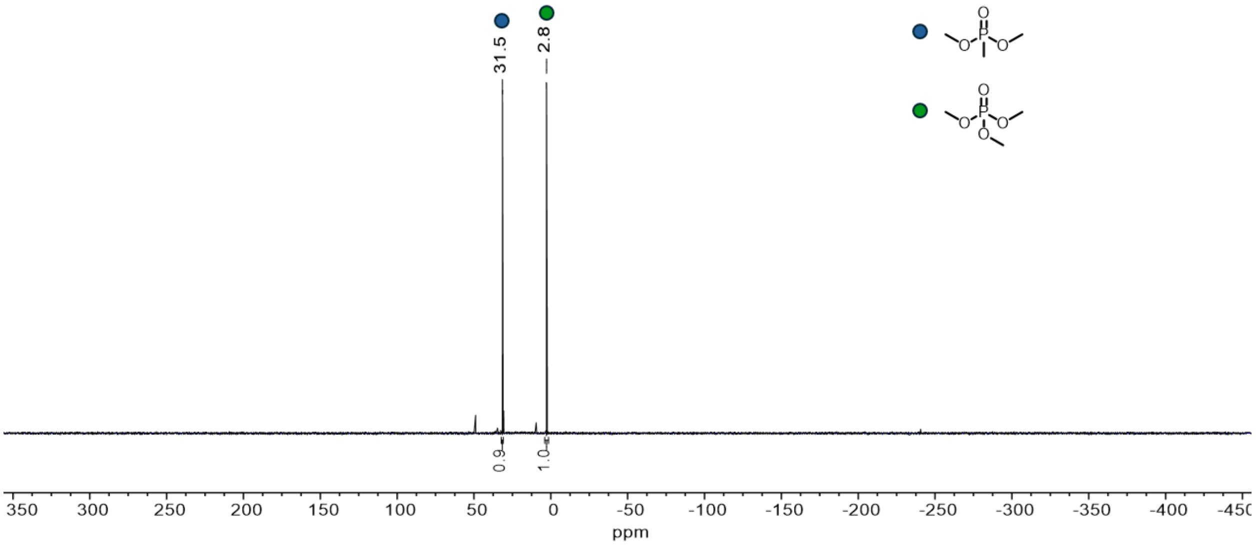

Figure S.64: $^{31}P\{^{1}H\}$ NMR spectrum (162 MHz, C_6D_6, $d1$ = 2 s, 298 K) of the DMMP generation.

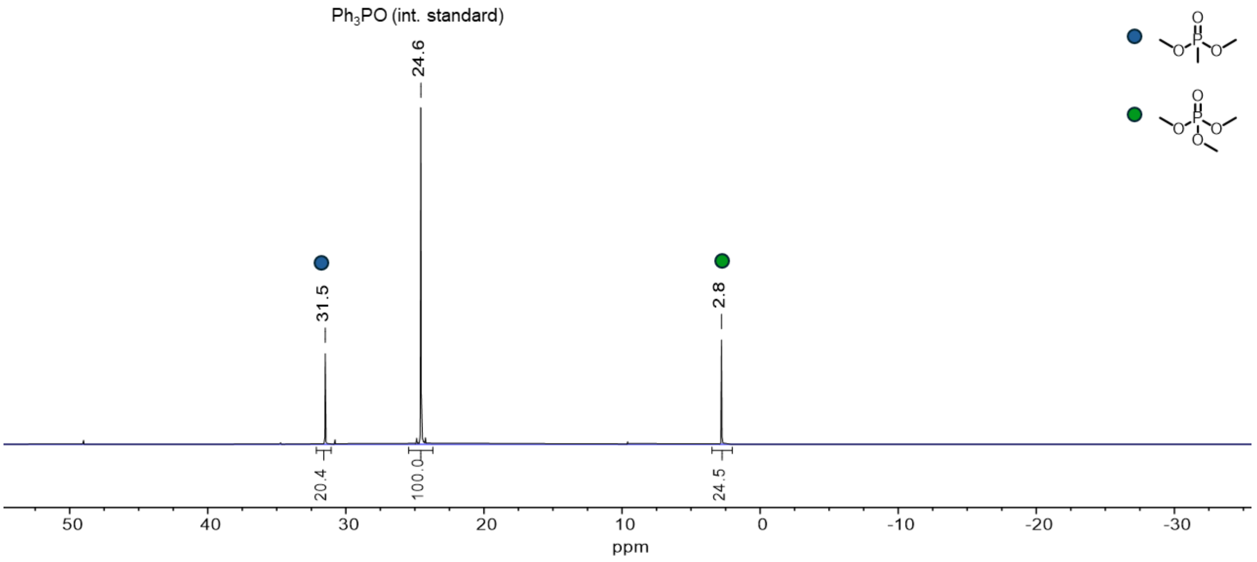

Figure S.65: $^{31}P\{^{1}H\}$ NMR spectrum (162 MHz, C_6D_6, $d1$ = 25 s, 298 K) of the DMMP generation after addition of Ph_3PO as the internal standard.

S.12.2 Generation of Dibenzyl Benzylphosphonate (DBBP)

Reaction of PO_3^{3-} with benzyl bromide gave the corresponding dibenzyl benzylphosphonate (DBBP) in approximately 32% ^{31}P NMR yield and was not isolated.

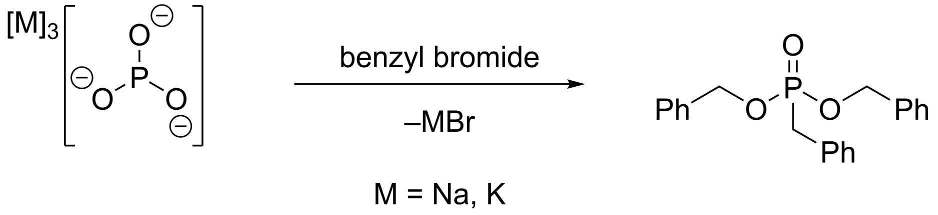

Figure S.66: Generation of dibenzyl benzylphosphonate.

Reaction: Inside the glovebox, the crude ball-milling mixture (500 mg, containing 0.18 mmol PO_3^{3-} (synthesized from $Na_3P_3O_9$ + K + KI, 10 w/w % K/KI, assuming 32% PO_3^{3-} yield, synthesized according to S.3.3) was loaded into a 50 mL Schlenk flask. Afterwards, an excess of benzyl bromide (1.00 mL) was added, and the flask was tightly closed. The flask was taken out of the glovebox and then heated at 60 °C for two days. No visible change occurred. Therefore, the flask was heated at 100 °C for an additional three days, after which the suspension turned from black to grey. Afterwards, the flask was allowed to cool down to room temperature. All volatile materials were removed *in vacuo* and the flask was brought back into the glovebox. The remaining green/gray solid was extracted with THF (1 mL) and all volatile materials were removed again *in vacuo*. The entire sample was dissolved in C_6D_6 and analyzed *via* $^{31}P\{^1H\}$ NMR spectroscopy.

Yield determination: Inside the glovebox, Ph_3PO (27.8 mg, 0.100 mmol) was added to the sample as internal standard. The mixture was analyzed via quantitative $^{31}P\{^1H\}$ NMR spectroscopy. The NMR tube was gently heated to facilitate dissolution. The solution exhibited an almost imperceptible turbidity, which was considered negligible. Calculated yield: 0.0570 mmol, 32%.

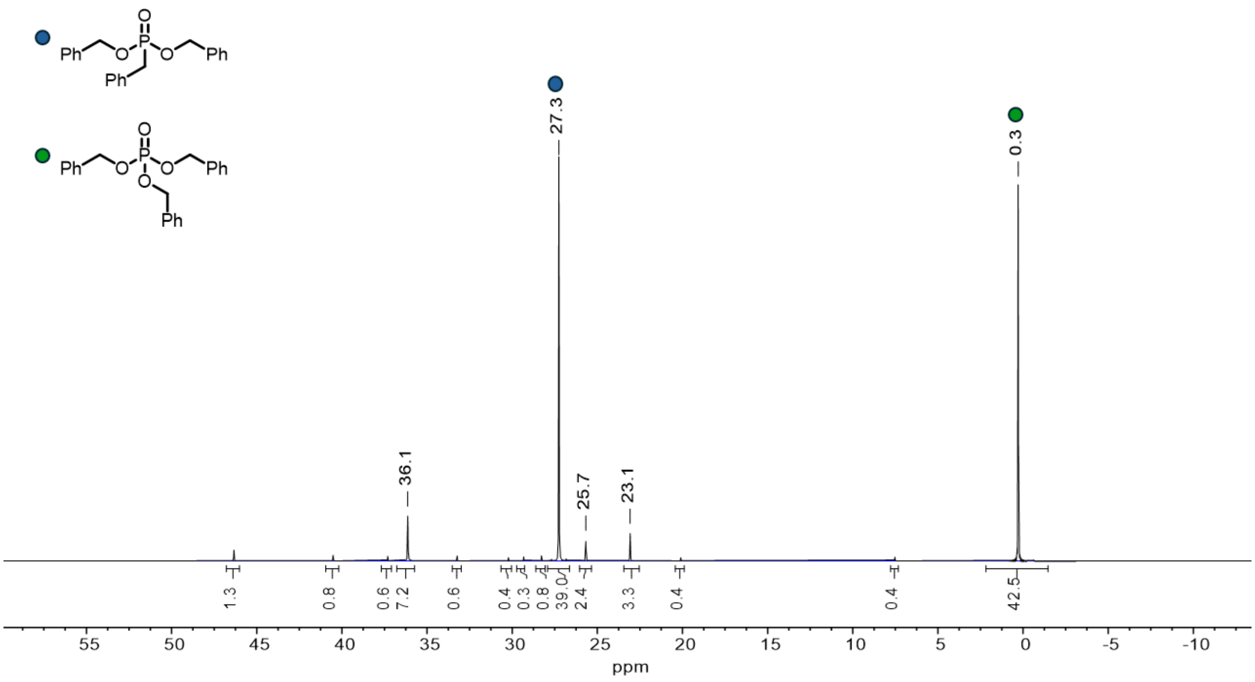

Figure S.67: $^{31}P\{^1H\}$ NMR spectrum (162 MHz, C_6D_6, $d1$ = 2 s, 298 K) of the DBBP reaction mixture.

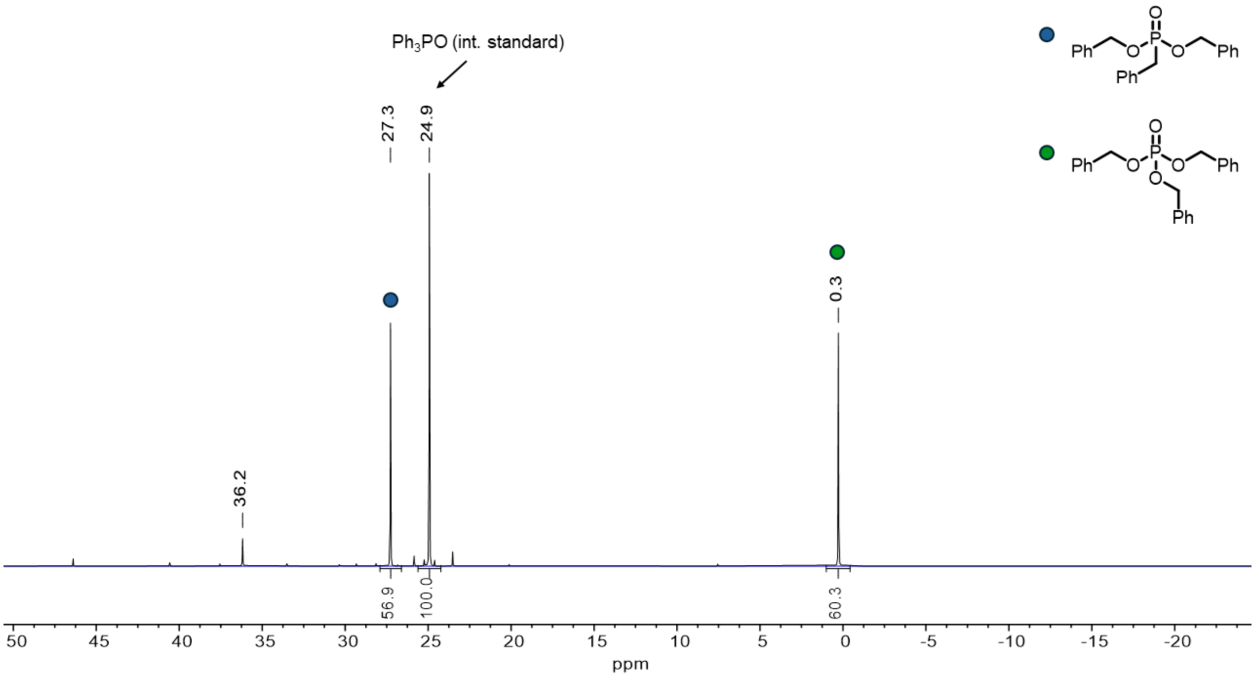

Figure S.68: $^{31}P\{^1H\}$ NMR spectrum (162 MHz, C_6D_6, $d1$ = 25 s, 298 K) of the DBBP reaction mixture after addition of Ph_3PO as the internal standard.

S.12.3 Reaction of the PO_3^{3-}-Containing Crude Mixture with $GeMe_3Cl$

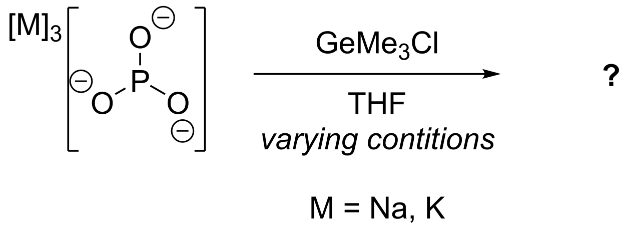

Figure S.69: Reaction of the PO_3^{3-}-containing crude mixture with $GeMe_3Cl$.

Reaction: Inside the glovebox, the crude ball-milling mixture (3.62 g, containing approximately 1.60 mmol PO_3^{3-}, synthesized according to S.10) was loaded into a 20 mL vial. Afterwards, 3 mL of THF and an excess of $GeMe_3Cl$ (4.71 g) were added, and the vial was tightly closed. The mixture was vigorously stirred for two days.

Purification attempt: The suspension was filtered through Celite® and the solid residue was washed twice with 2 mL THF. All volatile materials were removed *in vacuo* (see Figure S.70 for ^{31}P NMR spectrum). To the crude mixture, 15 mL n-hexane were added, resulting in a suspension. The precipitate was allowed to settle. Afterwards, the supernatant was pipetted off and passed through a glass filter. The volatile materials were removed and an oily liquid with a small amount of solid precipitate remained. The mixture was dried *in vacuo* for 4 h at 22 °C (final pressure 0.045 mbar) while stirring. Afterwards, the oil was separated by taking it up with a pipette and passing it through a glass filter. Then, 0.5 mL hexane was added to the oil. The solution was stored at −35 °C, resulting in more precipitate. The suspension was passed through a glass filter (pre-cooled to −35 °C). The final product is a dark yellow oil, which is still a mixture of products. Final yield: 146 mg. (see Figure S.71 for ^{31}P NMR spectrum)

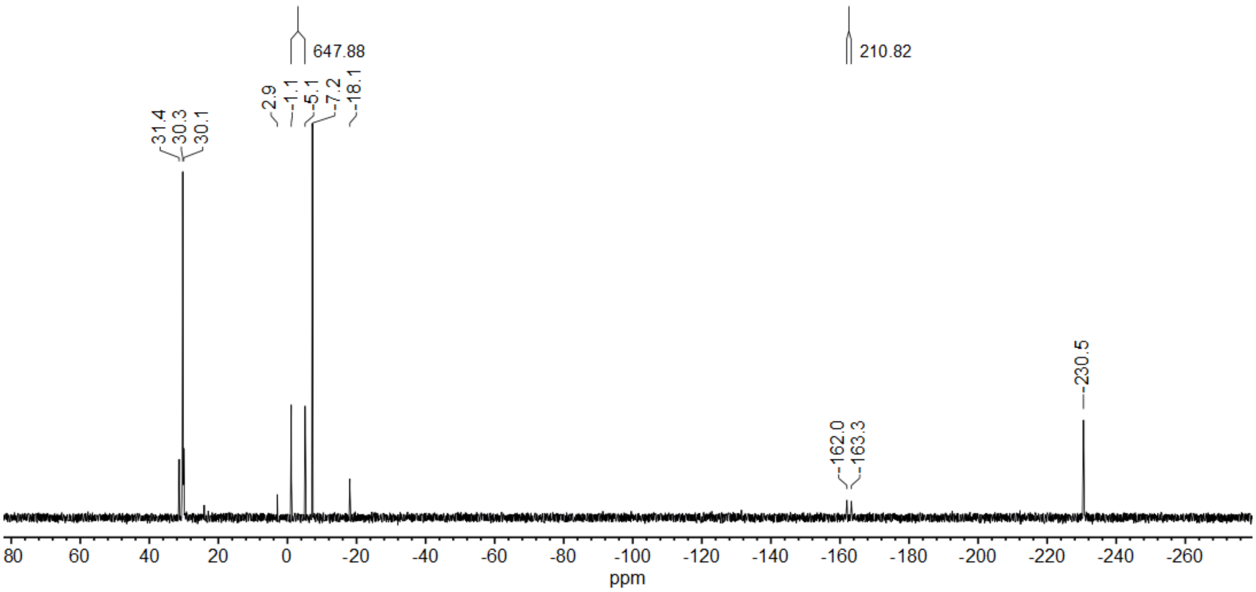

Figure S.70: ^{31}P NMR spectrum (162 MHz, THF, $d1 = 20$ s, 298 K) of the mixture from the reaction between the PO_3^{3-} containing crude mixture and $GeMe_3Cl$ after the first filtration.

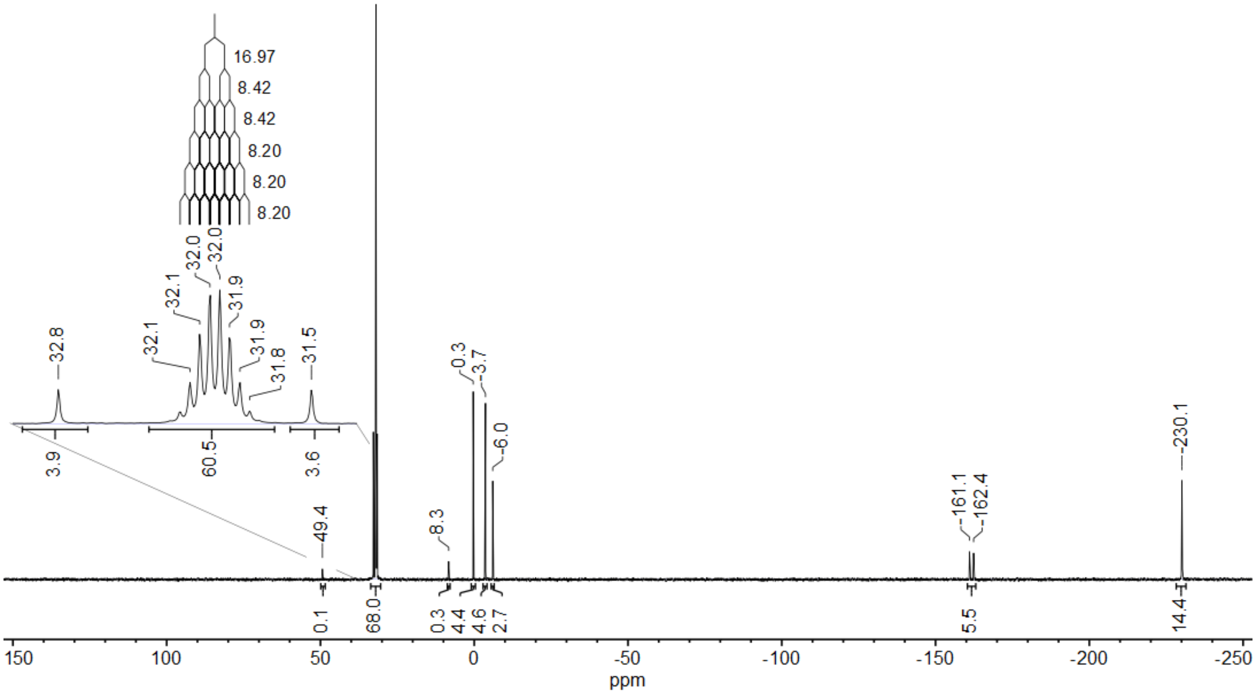

Figure S.71: ^{31}P NMR spectrum (162 MHz, THF, $d1 = 40$ s, 298 K) of the mixture from the reaction between the PO_3^{3-}-containing crude mixture and $GeMe_3Cl$ after the full purification attempt.

S.13 Computed Raman Spectra

S.13.1 General

Calculations were performed with the ORCA 6.0 program package.[10] Geometry optimizations and frequency calculations were performed using the ωB97X-3c method.[11–18] No imaginary frequencies were obtained, therefore confirming the calculated structure to be a local minimum.

S.13.2 Optimization of the Na_3PO_3 and K_3PO_3 Model Structures

Starting coordinates were obtained from a solid-state structure of Na_3SbO_3.[19] The Na_3PO_3 model was created by taking a representative fragment from the lattice containing eight Na_3SbO_3 molecules and replacing Sb with P as a starting point for gas-phase calculations. The K_3PO_3 model was created by replacing Na with K in the optimized Na_3PO_3 model prior to optimization.

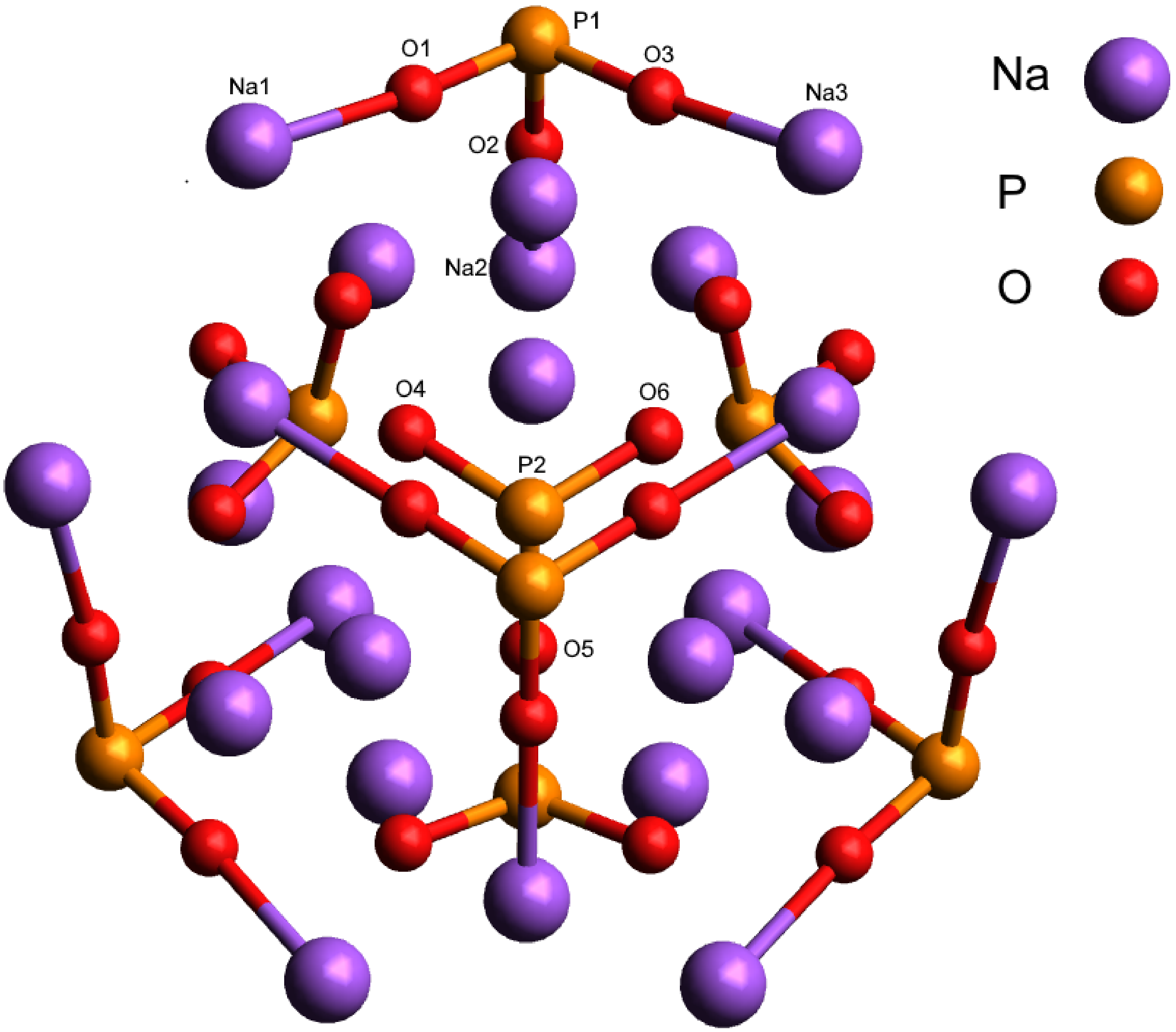

Figure S.72: Optimized structure of the Na_3PO_3 model compound. Selected bond lenghts [Å] and angles [°]: P1–O1 1.644, P1–O2 1.644, P1–O3 1.644, O1–Na1 2.136, O2–Na2 2.138, O3–Na3 2.137, P2–O4 1.638, P2–O5 1.638, P2–O6 1.638, O1-P1-O2 100.0, O4-P2-O5 103.4.

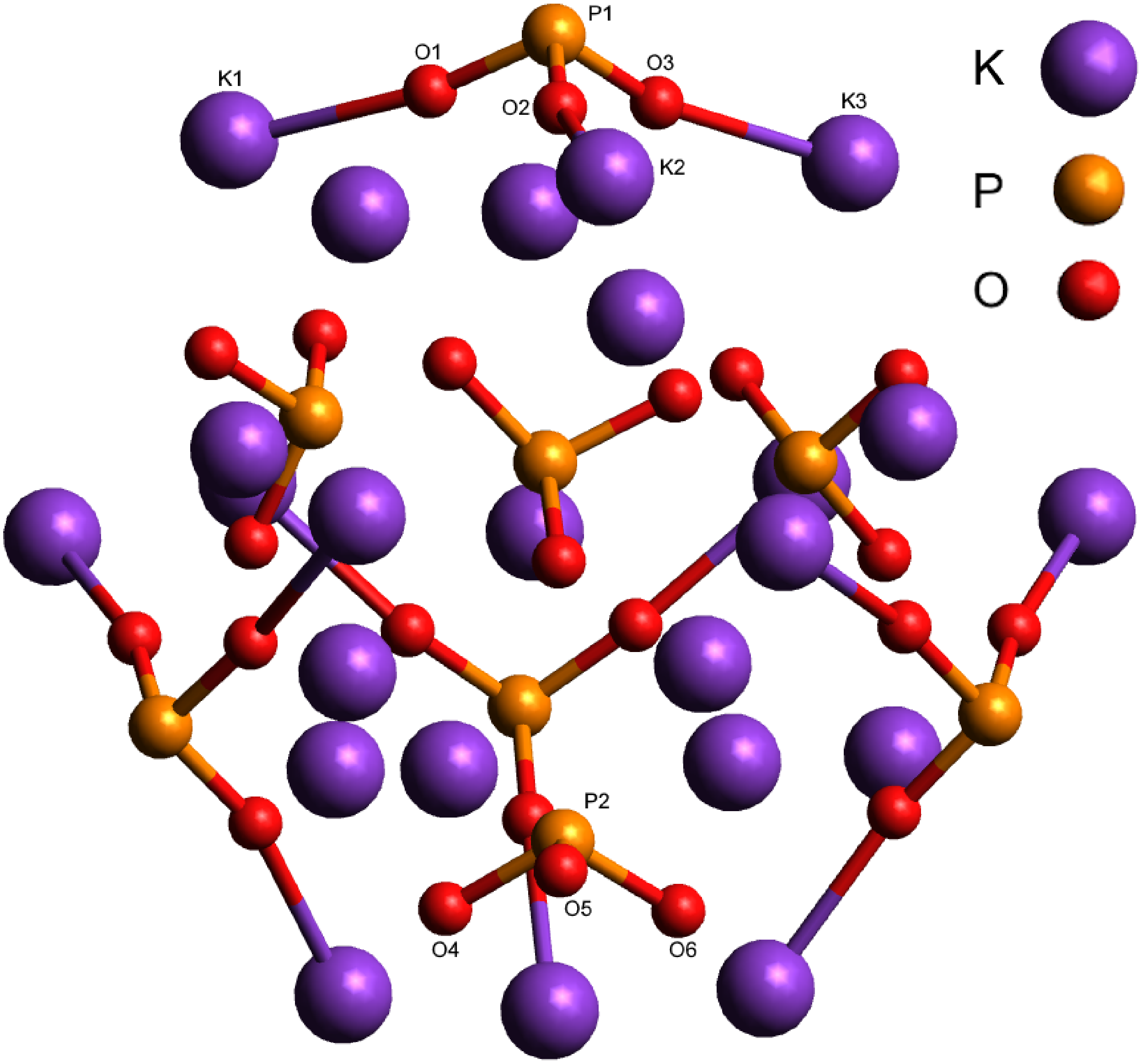

Figure S.73: Optimized structure of the K_3PO_3 model compound. Selected bond lenghts [Å] and angles [°]: P1–O1 1.651, P1–O2 1.636, P1–O3 1.632, O1–K1 2.492, O2–K2 2.459, O3–K3 2.452, P2–O4 1.618, P2–O5 1.623, P2–O6 1.624, O1-P1-O2 101.5, O4-P2-O5 104.3.

S.13.3 Raman Spectra of the Na_3PO_3 and K_3PO_3 Model Structures

The computed spectra were plotted using Chemcraft 1.8.[20] Band broadening is Gaussian and set to 30 cm^{-1} width on half height.

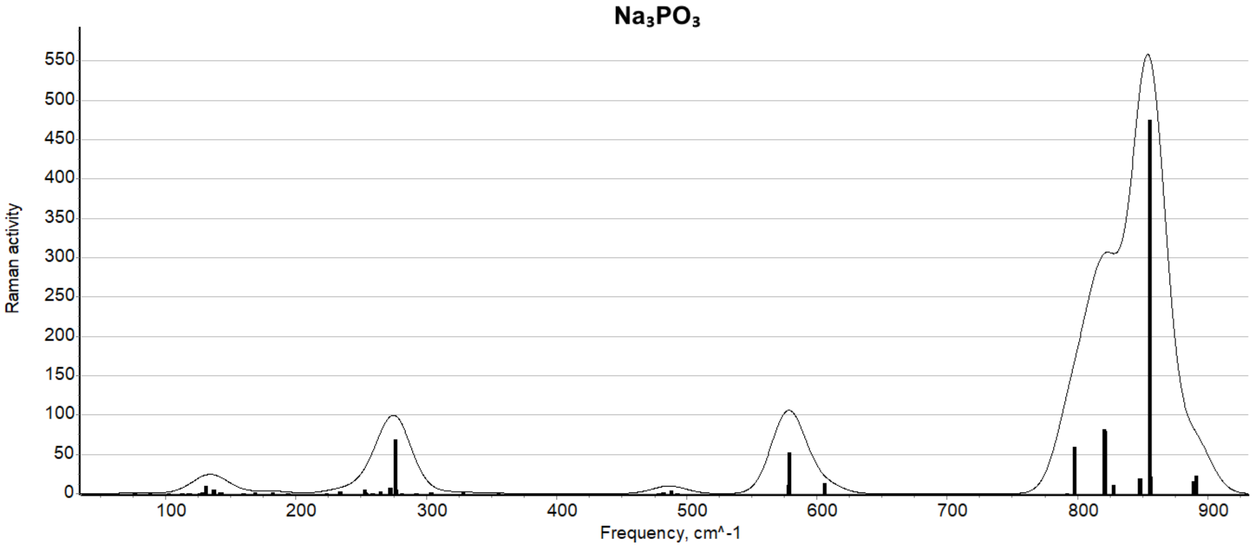

Figure S.74: Raman spectrum of the computed Na_3PO_3 model compound.

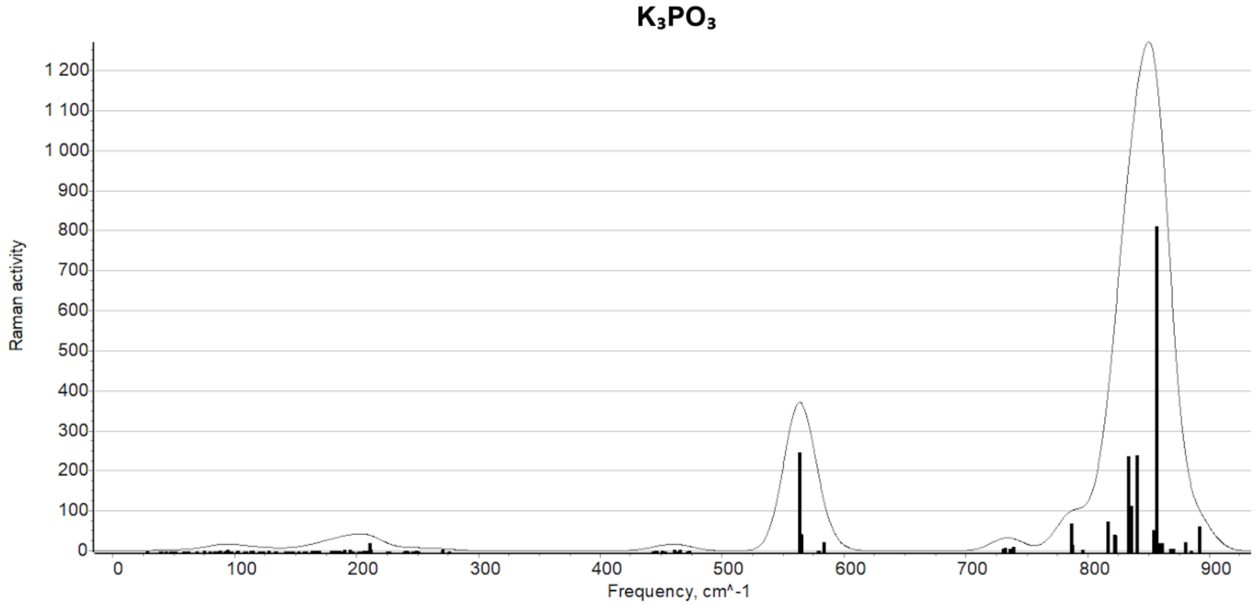

Figure S.75: Raman spectrum of the computed K_3PO_3 model compound.

S.13.4 XYZ-files

Table S.6: Atomic coordinates of the Na_3PO_3 model.

Element	X	Y	Z
Na	4.14286218962629	8.33740597578196	1.22636297251676
O	3.38733390339825	6.17833613716609	1.56839055427591
Na	1.22541154147330	8.33768245420650	4.14124650661021
O	3.38121237292410	8.00217410201662	3.38172453555337
Na	1.23150633001578	5.42309864390014	1.22566757788278
P	3.17837991470325	6.38885829604518	3.17973304285721
O	1.56652246892724	6.17945317304314	3.38462442208501
Na	8.33649929867295	8.33797166315227	5.42131444858260
O	6.17883912907962	7.99617916576098	6.17750375545974
Na	8.33875109423199	5.42146207384562	8.33810124897324
O	8.00148754564958	6.18133974366309	6.18142960470972
Na	5.42231203646113	8.33733992400528	8.33473266049764
P	6.38871655609667	6.38476398544278	6.38608345962199
O	6.18080618078437	6.17990714659593	7.99820994992009
Na	8.33906181071181	4.14349528330282	1.22512545635557
O	7.99888644625252	3.38607563326369	3.38344101252317
Na	5.42166730907959	1.22712913154653	1.22745045071752
O	6.18365038978199	3.38169789665497	1.56109012122374
Na	8.33773502419217	1.23070885228189	4.14128097264306
P	6.38701229312061	3.18028655131175	3.17436799041472
O	6.17873498419859	1.56915084880006	3.38581205224650
Na	4.14349767922910	1.22534880690996	8.33775052797665
O	3.38505821983913	3.38294375208865	7.99891672984833
Na	1.22561269249664	1.22646779561854	5.42116607078102
O	3.38201971884885	1.56262898749004	6.18194462792353
Na	1.23077747038535	4.14267353381145	8.34103990661784
P	3.17674351820550	3.17497748282460	6.38721033997955
O	1.56503502333744	3.38346607145746	6.18143437253896
Na	8.17004752552295	3.76562362634424	5.79847539370874
O	8.00942159720472	1.55413492671309	6.22835346892339
Na	5.79603172178591	3.76584732170461	8.16713041256433
O	8.00801848941751	3.33541119424556	8.00874222063828
Na	5.79831120019321	1.38823429752230	5.79818459407243
P	7.85759435364434	1.70573816878778	7.85797340134586
O	6.22788732700533	1.55449022613917	8.00763570641934
Na	3.76658581402915	3.76506255218317	1.39390260319242
O	1.55477666465103	3.33642162347701	1.55472681715058
Na	3.76647188752198	1.40120757830178	3.76901556642096
O	3.33550826325523	1.55565287095620	1.55682628286622
Na	1.38912649612791	3.76750228360206	3.76596054596426

P	1.70583672280936	1.70688679715083	1.70733036142581
O	1.55468511476093	1.55794146084878	3.33704682018339
Na	3.76425219123373	8.17082942818511	5.79643850770758
O	3.33494961756816	8.01059922296761	8.00755224432327
Na	1.38925715490383	5.79588628092873	5.79855203578377
O	1.55344513713901	8.00731961270481	6.22758380040437
Na	3.76764032399170	5.79844006611194	8.16331942418983
P	1.70542536700745	7.85754150819770	7.85731168407376
O	1.55618331274912	6.22778419885035	8.00869914320508
Na	8.17053038971370	5.79713439090918	3.76824660567048
O	8.00774970193909	8.00851747009628	3.33664467400021
Na	5.79771278377007	5.79535640941213	1.39523750229555
O	8.00883974595523	6.22763198522578	1.55698784030308
Na	5.79571897457198	8.16799173821333	3.76736558069062
P	7.85677972971320	7.85709049638509	1.70722240018390
O	6.22707925008991	8.00669915184693	1.55637899095560

Table S.7: Atomic coordinates of the K_3PO_3 model.

Element	X	Y	Z
K	4.11286805687187	8.53383465139200	0.61309929113418
O	2.86943136484023	6.30501466505439	1.19609994511898
K	1.04763388987722	8.92663149371720	4.15804631193540
O	3.25765698976053	7.87381134939826	3.17969489511438
K	0.48408799419857	5.41614241005377	0.64035491783871
P	2.68263976399583	6.40402215921233	2.80081067951350
O	1.08112855878299	6.45453638807024	3.06491907230445
K	9.08858658634546	8.92142164428523	5.41356541989962
O	6.70618725173950	8.37978620916945	6.31677004458965
K	8.50308459516465	5.41303586983133	8.93379170408574
O	8.47938025166198	6.49729790516279	6.46100445647639
K	5.44138430545807	8.95597279190375	8.53207935048917
P	6.87996510990418	6.77373453063995	6.40679138838836
O	6.29725413907756	6.38788257469667	7.87159123484112
K	8.51407949358163	4.15851294376278	0.63626835539222
O	8.47443175277136	3.07126226695143	3.11108330215117
K	5.43981349448095	0.60586658772314	1.03912295670780
O	6.30076869338353	3.17936695913276	1.68598630769972
K	9.07625391724105	0.64411244534356	4.14258075978360
P	6.87329334702877	2.80235243160606	3.15763652348545
O	6.69239014006719	1.19729363721261	3.25676005023321
K	4.13300651758903	1.03865559550918	8.96398425055616
O	2.85985145006774	3.24318804069013	8.37981100734112
K	1.06514234277275	0.63096206259656	5.41099884494486
O	3.27360035554435	1.68884677295338	6.38836073994518
K	0.47884156066273	4.14243795606707	8.91899282539270
P	2.69320575273746	3.15524646289629	6.77327123472042
O	1.09436962081898	3.10541676929644	6.49038406498364
K	9.02474486716113	3.62821097700998	5.84405588570018
O	8.31943062929884	1.22980811886420	6.44161584041376
K	5.82451824746040	3.69045777845930	7.05479113195665
O	8.20908110085672	3.09570483399617	8.16712378918491
K	5.87104027978623	0.50162394947313	5.75717796277926
P	8.04382654477618	1.47219232174709	8.05130390239927
O	6.42630802893112	1.28333836470013	8.13453256983261
K	3.69131918641024	3.80436004742444	0.49455418006401
O	1.24735641362958	3.11800544155190	1.22437199297535
K	3.74989240379603	2.49084921536923	3.68929195384744
O	3.14311691661346	1.42784670715165	1.27804333016828
K	0.56249553549020	3.72301279058628	3.62786324778598
P	1.52508511823439	1.50885857699133	1.46822810912085
O	1.36196621362559	1.39376533928110	3.09197638548186

K	3.75969160151458	7.08288098558780	5.87618642106537
O	3.14652963939944	8.12965482701499	8.28954793585143
K	0.59155409163862	5.82927779958859	5.93015778231653
O	1.36739369054389	8.16575965382809	6.47371740971078
K	3.69855579253864	5.74449993436823	9.05688153174637
P	1.52876434762385	8.04752747843254	8.09776653906204
O	1.25187514791199	6.43820349641924	8.33811588730927
K	8.94621190329135	5.92720083981338	3.73466725011254
O	8.30846067293948	8.33990439357545	3.11924304886720
K	5.79407913792672	5.87784873403656	2.46080122096023
O	8.18809887744260	6.47589738582050	1.39114553077777
K	5.86836483531646	9.06376393487369	3.81487180950834
P	8.03000567447103	8.10008625324148	1.51013599856478
O	6.41189580493965	8.29481424645942	1.42997141736465

S.14 ^{31}P NMR Shielding Calculations

The approach taken here is along the lines recommended by Latypov et al.[21] These authors make a convincing case to practitioners of ^{31}P NMR methods that simple GIAO quantum chemical calculations should be regarded as a routine structural tool.

A predicted chemical shift (δ) is obtained from a calculated chemical shielding (σ) according to equation 1:

$$\delta = \sigma_{ref} - \sigma + \delta_{ref} \quad (1)$$

Here, "ref" refers to a suitable reference substance of known chemical shift. It is also common to generate a correlation using many (σ, δ) data pairs from a collection of reference compounds, and to perform a linear regression. Good correlations can be obtained in this manner using gas phase chemical shielding calculations together with chemical shift observations in the liquid phase.[21] Gas-phase cluster calculations have also been used for the prediction of ^{31}P NMR chemical shifts for solid-state orthophosphate salts.[22]

For the quantum chemical calculations, geometries were fully optimized using the PBE0 functional[23] together with the def2-TZVP basis set[24] provided by ORCA 6.0,[25] and Grimme's D3BJ dispersion correction was employed.[26] The PBE0 D3BJ method was used similarly for the NMR shielding calculations, but for these the locally dense pcSseg-3 basis set was employed.[27] The NMR shielding calculations utilize gauge-including atomic orbitals and approximate two-electron integrals.[28]

A structural model of Na_3PO_3 was derived from the reported crystal structure of Na_3SbO_3.[29] The neutral cluster $[Na_3PO_3]_8$, upon geometry optimization, retained the essential structural features of the bulk phase on which it was modeled. It should be noted that the structural model has two phosphorus positions that are chemically distinct, resulting in two calculated values of the ^{31}P NMR shielding (σ): 189.085 and 182.352 ppm. Both of those are averages of four values for almost identical sites in the cluster; no symmetry was imposed in the geometry optimization of the structure.

For our reference, we used a simple model of sodium orthophosphate wherein the PO_4^{3-} ion is surrounded by six sodium cations which, upon geometry optimization, adopted a symmetrical disposition with one sodium ion approaching each edge of the tetrahedral orthophosphate ion. This reference system gave a computed chemical shielding (σ) for the ^{31}P nucleus of 276.522 ppm. The ^{31}P NMR chemical shift (δ) of sodium orthophosphate in the solid state is known experimentally to be 14.0 ppm.[30]

Using Eq. 1 above, the calculations predict ^{31}P NMR chemical shifts (δ) for $[Na_3PO_3]_8$ of 101.4 and 108.2 ppm, in quite good agreement with the experimentally observed value. Furthermore, the small value of the calculated magnetic shielding anisotropy (ca. 69 and 146 ppm for the two positions) is also a good match, qualitatively, with the experimental data.

It is noted that a simpler model system for Na_3PO_3, namely the gas-phase geometry optimized monomer with three edge-located sodium ions gives a poorer fit to the experimental data than does the cluster model $[Na_3PO_3]_8$ discussed above, that was based upon the solid-state crystal structure of sodium antimonite. The Na_3PO_3 gas-phase monomer yields a calculated $\sigma = 219.331$ ppm, corresponding to a calculated ^{31}P NMR chemical shift $\delta = 71.2$ ppm. It is seen that the calculated chemical shift can easily change more than 20 ppm using

this gas-phase computational approach to estimating a solid-state NMR chemical shift for PO_3^{3-} depending upon the specifics of the chosen model. However, the calculated chemical shifts are still very much in the correct range given that the full ^{31}P NMR chemical shift window is greater than 1800 ppm in width.

S.14.1 XYZ-files

Table S.8: Atomic coordinates of the Na_3PO_3 monomer.

Element	X	Y	Z
O	-0.07083276826489	0.16154471600395	0.10136771715339
P	1.52971508418946	0.04235884450305	0.02486830659012
O	2.00272590588211	0.17628450919432	1.55456981784983
O	1.98965999952119	1.42816569376517	-0.64546328028620
Na	3.23693399412774	1.87945662822910	1.05505677636158
Na	-0.02086101150105	-0.12323686477442	2.24173977190943
Na	-0.04080120395457	1.85131647307884	-1.24204910957817

Table S.9: Atomic coordinates of the Na_3PO_3 octamer.

Element	X	Y	Z
Na	4.15124881069640	8.31522773655169	1.24840805548716
O	3.39831123148464	6.16588743858788	1.60045333053526
Na	1.24775272547333	8.31623882102632	4.15130245108476
O	3.39640886359230	7.96639065997459	3.39687952927532
Na	1.24889821769352	5.41282537280943	1.24891461769640
P	3.18331019696588	6.38147493017861	3.18448438261556
O	1.59844763434782	6.16688347172494	3.39774282444454
Na	8.31532178107892	8.31565181456582	5.41263939208491
O	6.16589075521928	7.96414695808221	6.16585276525820
Na	8.31601195378369	5.41227346014416	8.31594352862655
O	7.96594732838248	6.16690542227788	6.16695554509805
Na	5.41241824674644	8.31498357255987	8.31570337705167
P	6.38123629790017	6.37986204214341	6.38076342361021
O	6.16721478592597	6.16645499874250	7.96566025297197
Na	8.31640928200894	4.15164905415072	1.24809178161819
O	7.96477731953860	3.39856262259133	3.39746334493650
Na	5.41260312254070	1.24740898395696	1.24915705124748
O	6.16758562458483	3.39646802032288	1.59714341084073
Na	8.31558613632442	1.24938327212948	4.15119730866689
P	6.38035757011088	3.18434976414027	3.18225211818769
O	6.16623084575591	1.59976595758145	3.39812818976120
Na	4.15167664125273	1.24854363802838	8.31670108222215
O	3.39792902764784	3.39743324016546	7.96504730379638

Element	X	Y	Z
Na	1.24896676632971	1.24738009759131	5.41275465850123
O	3.39763683036108	1.59806754420641	6.16736311415519
Na	1.24904075328614	4.15127906310253	8.31605863954450
P	3.18388346782747	3.18280275062294	6.38062652511828
O	1.59935014442314	3.39725760717693	6.16645408197461
Na	8.09036034723616	3.78350418811647	5.78039067531267
O	7.98769393221462	1.57628213402539	6.22312993671470
Na	5.78028706847323	3.78303142935426	8.08990560366843
O	7.98782939626984	3.34044164323877	7.98829719734256
Na	5.78069781777636	1.46963843323670	5.78066086849685
P	7.82700242631728	1.73685506478966	7.82663980174666
O	6.22351855945220	1.57489504554695	7.98819090562769
Na	3.78380873900661	3.78350943449990	1.47162765724732
O	1.57677083207726	3.34110838950166	1.57623282184763
Na	3.78356785624069	1.47804897332691	3.78457636496006
O	3.34077771583096	1.57577635626747	1.57680058929951
Na	1.47177758851057	3.78419889542616	3.78337965124485
P	1.73717495461617	1.73761147003095	1.73759550900194
O	1.57524896166965	1.57681599055304	3.34111507967180
Na	3.78286341402417	8.09228751883897	5.77971046106054
O	3.34064849404200	7.98759751510730	7.98656982081849
Na	1.47226145734131	5.78017288668579	5.78045553255872
O	1.57473575632348	7.98781656516858	6.22313016665623
Na	3.78397923854085	5.78037994671538	8.08782009247257
P	1.73713501261475	7.82664059942557	7.82664382276069
O	1.57576980368994	6.22308341136183	7.98799939922209
Na	8.09124437141035	5.78086974958149	3.78382539994203
O	7.98748681642477	7.98835644598963	3.34077201463257
Na	5.78090924302564	5.77997117203059	1.47378617981130
O	7.98836389337733	6.22333465326100	1.57630487329057
Na	5.78011104190531	8.08978444676203	3.78332619912063
P	7.82648061964982	7.82678484910511	1.73729451361438
O	6.22304228065482	7.98764447691538	1.57567677544428

Table S.10: Atomic coordinates of the Na_6PO_4 monomer.

Element	X	Y	Z
O	-0.00229861513121	0.07464319795825	0.16588290808581
P	1.54357289816207	0.05130236737517	0.04833666455312
O	2.16770131417212	0.04521410704308	1.46784257203068
O	2.02582480298264	1.30802733571388	-0.72059299877586
O	1.98286503161772	-1.22284649006490	-0.71723545272629
Na	3.28708182670032	-2.02719224854526	1.14734898052473

Element	X	Y	Z
Na	3.05876546560645	0.00499345144883	-2.46054828749677
Na	-0.25287383347816	-2.00847411891924	-1.01417392517969
Na	-0.20230048545929	2.13309668874203	-1.05013176225016
Na	0.01656475712657	0.09875074568824	2.55524873709861
Na	3.33593683770075	2.11597496355990	1.10791256413581

S.15 References

(1) Pangborn, A. B.; Giardello, M. A.; Grubbs, R. H.; Rosen, R. K.; Timmers, F. J. Safe and Convenient Procedure for Solvent Purification. *Organometallics* **1996**, *15*, 1518–1520.

(2) Williams, D. B. G.; Lawton, M. Drying of Organic Solvents: Quantitative Evaluation of the Efficiency of Several Desiccants. *J. Org. Chem.* **2010**, *75*, 8351–8354.

(3) Fulmer, G. R.; Miller, A. J. M.; Sherden, N. H.; Gottlieb, H. E.; Nudelman, A.; Stoltz, B. M.; Bercaw, J. E.; Goldberg, K. I. NMR Chemical Shifts of Trace Impurities: Common Laboratory Solvents, Organics, and Gases in Deuterated Solvents Relevant to the Organometallic Chemist. *Organometallics* **2010**, *29*, 2176–2179.

(4) Zhai, F.; Xin, T.; Geeson, M. B.; Cummins, C. C. Sustainable Production of Reduced Phosphorus Compounds: Mechanochemical Hydride Phosphorylation Using Condensed Phosphates as a Route to Phosphite. *ACS Cent. Sci.* **2022**, *8*, 332–339.

(5) Clegg, W.; Conway, B.; Kennedy, A. R.; Klett, J.; Mulvey, R. E.; Russo, L. Synthesis and Structures of [(Trimethylsilyl)methyl]sodium and -potassium with Bi- and Tridentate N-Donor Ligands. *Eur. J. Inorg. Chem.* **2011**, *2011*, 721–726.

(6) Schlosser, M.; Hartmann, J. Transmetalation and Double Metal Exchange: A Convenient Route to Organolithium Compounds of the Benzyl and Allyl Type. *Angew. Chem., Int. Ed.* **1973**, *12*, 508–509.

(7) Ropp, R. In *Encyclopedia of the Alkaline Earth Compounds*; Ropp, R., Ed.; Elsevier: Amsterdam, 2013; pp 199–350, Procedural details for isolating $BaHPO_3 \cdot H_2O$.

(8) Xiang, H.; Xu, H.; Wang, Z.; Chen, C. Dimethyl methylphosphonate (DMMP) as an efficient flame retardant additive for the lithium-ion battery electrolytes. *J. Power Sources* **2007**, *173*, 562–564.

(9) Velencoso, M. M.; Battig, A.; Markwart, J. C.; Schartel, B.; Wurm, F. R. Molecular Firefighting—How Modern Phosphorus Chemistry Can Help Solve the Challenge of Flame Retardancy. *Angew. Chem. Int. Ed.* **2018**, *57*, 10450–10467.

(10) Neese, F. Software Update: The ORCA Program System—Version 6.0. *Wiley Interdiscip. Rev. Comput. Mol. Sci.* **2025**, *15*, e70019, e70019 CMS-1186.R1.

(11) Müller, M.; Hansen, A.; Grimme, S. ωB97X-3c: A composite range-separated hybrid DFT method with a molecule-optimized polarized valence double-ζ basis set. *J. Chem. Phys.* **2023**, *158*, 014103.

(12) Neese, F. An improvement of the resolution of the identity approximation for the formation of the Coulomb matrix. *J. Comput. Chem.* **2003**, *24*, 1740–1747.

(13) Neese, F.; Wennmohs, F.; Hansen, A.; Becker, U. Efficient, approximate and parallel Hartree–Fock and hybrid DFT calculations. A 'chain-of-spheres' algorithm for the Hartree–Fock exchange. *Chem. Phys.* **2009**, *356*, 98–109.

(14) Helmich-Paris, B.; de Souza, B.; Neese, F.; Izsák, R. An improved chain of spheres for exchange algorithm. *J. Chem. Phys.* **2021**, *155*, 104109.

(15) Neese, F. The SHARK integral generation and digestion system. *J. Comput. Chem.* **2023**, *44*, 381–396.

(16) Caldeweyher, E.; Bannwarth, C.; Grimme, S. Extension of the D3 dispersion coefficient model. *J. Chem. Phys.* **2017**, *147*, 034112.

(17) Caldeweyher, E.; Ehlert, S.; Hansen, A.; Neugebauer, H.; Spicher, S.; Bannwarth, C.; Grimme, S. A generally applicable atomic-charge dependent London dispersion correction. *J. Chem. Phys.* **2019**, *150*, 154122.

(18) Caldeweyher, E.; Mewes, J.-M.; Ehlert, S.; Grimme, S. Extension and evaluation of the D4 London-dispersion model for periodic systems. *Phys. Chem. Chem. Phys.* **2020**, *22*, 8499–8512.

(19) Stöver, H.-D.; Hoppe, R. Über Oxoantimonate(III) und Oxobismutate(III) Zur Kenntnis von Na_3SbO_3 und Na_3BiO_3 [1]. *Z. Anorg. Allg. Chem.* **1980**, *468*, 137–147.

(20) Chemcraft - graphical software for visualization of quantum chemistry computations. Version 1.8, build 682. https://www.chemcraftprog.com.

(21) Latypov, S. K.; Polyancev, F. M.; Yakhvarov, D. G.; Sinyashin, O. G. Quantum chemical calculations of ^{31}P NMR chemical shifts: scopes and limitations. *Phys. Chem. Chem. Phys.* **2015**, *17*, 6976–6987.

(22) Martel, L.; Kovács, A.; Popa, K.; Bregiroux, D.; Charpentier, T. ^{31}P MAS NMR and DFT study of crystalline phosphate matrices. *Solid State Nucl. Magn. Reson.* **2020**, *105*, 101638.

(23) Adamo, C.; Barone, V. Toward reliable density functional methods without adjustable parameters: The PBE0 model. *J. Chem. Phys.* **1999**, *110*, 6158–6170.

(24) Weigend, F.; Ahlrichs, R. Balanced basis sets of split valence, triple zeta valence and quadruple zeta valence quality for H to Rn: Design and assessment of accuracy. *Phys. Chem. Chem. Phys.* **2005**, *7*, 3297–3305.

(25) Neese, F. Software update: the ORCA program system, version 5.0. *WIRES Comput. Molec. Sci.* **2022**, *12*, e1606.

(26) Grimme, S.; Antony, J.; Ehrlich, S.; Krieg, H. A consistent and accurate ab initio parametrization of density functional dispersion correction (DFT-D) for the 94 elements H-Pu. *J. Chem. Phys.* **2010**, *132*, 154104.

(27) Jensen, F. Segmented Contracted Basis Sets Optimized for Nuclear Magnetic Shielding. *J. Chem. Theory Comput.* **2015**, *11*, 132–138, PMID: 26574211.

(28) Stoychev, G.; Auer, A.; Izsak, R.; Neese, F. Self-Consistent Field Calculation of Nuclear Magnetic Resonance Chemical Shielding Constants Using Gauge-Including Atomic Orbitals and Approximate Two-Electron Integrals. *J. Chem. Theory Comput.* **2018**, *14*, 619–637.

(29) Stöver, H.-D.; Hoppe, R. Über Oxoantimonate(III) und Oxobismutate(III) Zur Kenntnis von Na_3SbO_3 und Na_3BiO_3 [1]. *Z. Anorg. Allg. Chem.* **1980**, *468*, 137–147.

(30) Hayashi, S.; Hayamizu, K. High-Resolution Solid-State ^{31}P NMR of Alkali Phosphates. *Bull. Chem. Soc. Jpn.* **1989**, *62*, 3061–3068.
